# Benchmarking precision matrix estimation methods for differential co-expression network analysis

**DOI:** 10.1186/s12859-026-06731-6

**Published:** 2026-11-11

**Authors:** Matthias Overmann, Gordon Grabert, Tim Kacprowski

**Affiliations:** 1 Institute of Data Science in Biomedicine, Technische Universität Braunschweig, Braunschweig, Germany; 2 Braunschweig Integrated Centre of Systems Biology (BRICS), Technische Universität Braunschweig, Braunschweig, Germany

**Keywords:** Differential co-expression, Gaussian graphical model, Precision matrix estimation, Partial correlation, Differential network analysis, High-dimensional data

## Abstract

**Background:**

Gene expression profiling is widely used to investigate disease mechanisms, but classical approaches such as differential expression or pairwise correlation analyses provide limited interpretability. Network-based differential co-expression methods that model conditional dependencies through partial correlations offer richer insights, yet their application in high-dimensional settings requires estimation of precision matrices. Numerous precision matrix estimation methods (PMEMs) have been proposed, but these are typically validated in a narrow range of simulation settings and benchmarked against only a small number of competing methods, so their relative performance under diverse conditions remains unclear.

**Results:**

Simulated gene expression datasets with known ground truth correlation structures were used to benchmark a broad set of PMEMs for their suitability in differential co-expression network analysis of transcriptomic data. Performance was strongly affected by data characteristics, including covariance structure, matrix density, covariance values, sample size-to-dimension ratio, and sampling distribution. Among the evaluated methods, *GLassoElnetFast* generally achieved high accuracy in recovering differential edges across the simulated scenarios, although high signal-to-noise ratios and sufficient sample sizes remain essential for reliable inference. Applying a subset of these methods to a real transcriptomic dataset reproduced this estimator-dependent variability, with limited overlap between the differential edges recovered by different methods.

**Conclusions:**

Evaluation across diverse simulation conditions demonstrated that no single metric or condition was sufficient to assess PMEM performance. Therefore, less extensive evaluations may only capture part of this variability and limit the generalizability of their conclusions. Our simulation and benchmarking framework supports future method development and ensures reproducible evaluation of newly developed approaches.

**Supplementary information:**

The online version contains supplementary material available at https://doi.org/10.1186/s12859-026-06731-6.

## Introduction

The transcriptome constantly responds to changing conditions, allowing cells to functionally adapt [​^1^]. This variability is commonly profiled using microarrays or RNA sequencing (RNA-seq) [2]. The standard analysis of such data, differential gene expression (DGE) analysis, aims to identify differentially expressed (DE) genes associated with a condition or predictor of interest [3–7], using established pipelines such as DESeq2 [​^8^], edgeR [9], and limma/voom [10; 11]. The resulting gene lists serve as the basis for downstream interpretation, including pathway analysis and biomarker discovery [4; 12; 13].

Beyond individual genes, pairwise relationships are commonly summarized using Pearson’s correlation [6; 14–16]. However, analysing genes or relationships in isolation neglects information that could be gained by considering the entirety of the expression profile [5]. Differential co-expression analysis addresses this by constructing condition-specific networks that capture changes in gene-gene relationships, using strategies such as module and hub identification, regulatory network construction, and guilt-by-association analysis [17]. Using Gaussian graphical models (GGMs), partial correlations obtained from the precision matrix highlight direct dependencies while filtering out indirect effects, yielding sparser and more interpretable networks [18]. However, incorporating the entire expression profile also introduces new challenges. Gene expression studies typically profile more than 20,000 genes in only a few hundred samples [19; 20], resulting in a high-dimensional setting [21] in which the sample covariance matrix becomes singular and cannot be inverted to obtain the precision matrix. Precision matrix estimation methods address this by imposing regularization or structural assumptions, enabling the recovery of stable and interpretable partial correlation networks. When new PMEMs are proposed, they are commonly validated in standard simulation settings and compared against only a limited number of competing methods. Additionally, the relative performance of existing precision matrix estimation methods for differential co-expression analysis has not been systematically evaluated across a variety of high-dimensional data scenarios.

In this study, a simulation framework is introduced to evaluate a wide range of PMEMs across diverse data scenarios that reflect properties of real gene expression data, with the aim of assessing their suitability for differential co-expression network analysis of transcriptomic data. Their performance is compared using multiple evaluation metrics to identify strengths, weaknesses, and practical considerations for this application. To examine whether these simulation-based findings extend to empirical data, a subset of the evaluated methods is further applied to a real-world transcriptomic dataset.

## Background

### Networks

A network, or graph, specifies relationships between components (nodes) via interactions (edges) and can be represented mathematically as an adjacency matrix [22; 23]. Real-world networks, including biological ones, commonly display non-random, scale-free topologies with a power-law degree distribution, *p*(*k*) ≈ *k*^−*γ*^, resulting in a few highly connected hub nodes and many sparsely connected ones [22–26]. In contrast, Erdős-Rényi random networks exhibit uniform degree distributions and lack hubs [26].

While DGE analysis has revealed insights into transcriptional regulation and disease biomarkers [27–30], it tends to highlight generic, well-characterized genes rather than the network-level disruptions that underlie many diseases [31; 32]. As biological networks change in response to development, external stimuli, or disease [33], comparing network topologies across conditions can reveal rewiring patterns and molecular mechanisms relevant to disease processes and therapeutic targets [34–36]. Differential network analysis addresses this directly by evaluating changes in interactions rather than individual components, allowing the detection of disease-related modules even when gene expression itself remains unchanged [33–35].

To obtain condition-specific networks for comparison, associations between variables must first be inferred within each condition, and a range of differential network analysis tools have been proposed for this purpose. These include the boosted-tree-based BoostDiff [37], the pathway-focused DINGO [38], the widely used WGCNA, which builds weighted co-expression networks from pairwise correlations under a scale-free topology assumption [17; 39], and GGM-based methods such as chNet, which tests for changes in partial correlations and expression levels between conditions [34]. Many of these approaches, including WGCNA and chNet, rely on correlation-based metrics to quantify associations between node pairs [34; 38; 39], which are introduced in more detail below.

### Correlation-based metrics

Empirical data are commonly summarized using descriptive statistics such as the mean, variance, covariance, and the Pearson correlation coefficient, defined in the standard way [40]. In the following, uppercase letters (e.g., *X*) denote random variables and lowercase letters (e.g., *x*_*ij*_) their observed realizations. Population quantities are estimated from a sample of size *n*.

For a *p*-dimensional random vector $X={({X}_{1},\ldots ,{X}_{p})}^{T}\in {{\mathbb{R}}}^{p}$ with mean vector $\mu ={\mathbb{E}}[X]$, the covariance matrix

1 $$\Sigma ={\rm{Cov}}(X)={\mathbb{E}}[(X-\mu ){(X-\mu )}^{T}]$$

has entries *σ*_*ij*_ = Cov(*X*_*i*_, *X*_*j*_). Given *n* i.i.d. realizations ${X}_{1},\ldots ,{X}_{n} \sim {{\mathcal{N}}}_{p}(\mu ,\Sigma )$, stored row-wise in a data matrix ${\bf{X}}\in {{\mathbb{R}}}^{n\times p}$, Σ is estimated by the sample covariance matrix ​

$$S=\dfrac{1}{n-1}\mathop{\sum }\limits_{i=1}^{n}({X}_{i}-\bar{X}){({X}_{i}-\bar{X})}^{T},$$

and the correlation matrix R, containing pairwise correlations ${\rho }_{ij}={\sigma }_{ij}/\sqrt{{\sigma }_{ii}{\sigma }_{jj}}$, represents its normalized counterpart.

The inverse of Σ, Σ^−1^, is known as the precision or concentration matrix Θ = (*θ*_*ij*_). Under $X \sim {{\mathcal{N}}}_{p}(\mu ,\Sigma )$, *θ*_*ij*_ = 0 implies that *X*_*i*_ and *X*_*j*_ are conditionally independent given all remaining variables, whereas *θ*_*ij*_ ≠ 0 indicates a direct dependency [41]. This allows Θ to be interpreted as the adjacency structure of an undirected graphical model, with edges corresponding to its non-zero off-diagonal entries.

In classical (low-dimensional) settings, where the sample size *n* grows large relative to a fixed number of variables *p*, S is invertible with probability tending to one and thus provides a consistent estimator of Θ via S^−1^ [42; 43]. In high-dimensional low sample size (HDLSS) settings (*p* ≫ *n*), however, S satisfies rank(S) ≤* n* − 1 and is therefore singular whenever *p *≥ *n*, so that S^−1^ cannot be computed and cannot serve as an estimator of Θ [43]. This has motivated alternative estimators for large covariance or precision matrices [44; 45], including banding [46], tapering [47], thresholding [45], modified Cholesky decomposition with regularization [48–50], and sparse precision matrix estimation using the graphical lasso [51]. Sparse precision matrices are particularly relevant for graphical models, as zero partial correlations correspond to absent edges in the inferred network [41; 45], enabling the identification of subnetworks of variables that are conditionally independent of others.

### Gaussian graphical models

Probabilistic graphical models (PGMs) represent joint probability distributions through graphs and encode conditional independence structure to reduce computational complexity in inference. They are widely used for modeling uncertainty in fields such as computer vision, time-series analysis, and bioinformatics [52]. Gaussian graphical models (GGMs), a PGM class assuming multivariate Gaussian data, are commonly used to infer statistical dependencies among biological variables from high-throughput omics data [34; 53–55]. Unlike correlation-based approaches, which capture only marginal relationships and may conflate direct and indirect effects, GGMs use full-order partial correlations to distinguish between them, adjusting for the influence of all other variables and yielding more accurate network representations [53; 56; 57]. Since biological variables rarely lack both direct and indirect associations, correlation networks are typically dense. Therefore, sparsity must be imposed through thresholding, which introduces arbitrary cut-offs [56]. GGMs avoid this by modeling conditional independence directly. As noted above, zero entries of the precision matrix Θ correspond to conditionally independent variable pairs and thus to absent edges, so the resulting partial correlation networks are naturally sparse [34; 53; 55].

### Precision matrix estimation

Partial correlations are used by GGMs to quantify linear associations between two variables after removing the effect of all others [58]. For a *p*-dimensional random vector $X={({X}_{1},\ldots ,{X}_{p})}^{T}$, the partial correlation between *X*_*i*_ and *X*_*j*_, given all remaining variables {*X*_*k*_: *k* ≠ *i*, *j*}, is defined as

2 $${\rho }_{ij\cdot -ij}=-\dfrac{{\theta }_{ij}}{\sqrt{{\theta }_{ii}{\theta }_{jj}}}.$$

The partial correlation matrix Ψ = (*ρ*_*ij* ⋅ −*ij*_) thus contains the normalized off-diagonal entries of Θ and can be written in matrix form as

$$\Psi =-{D}_{\Theta }^{-1/2}\,\Theta \,{D}_{\Theta }^{-1/2},$$

where *D*_*Θ*_ = diag(*θ*_11_, …, *θ*_*pp*_) [59; 60].

As established above, S^−1^ cannot be computed in high-dimensional settings, so Θ, and consequently Ψ, must be estimated directly [42; 61–63]. Precision matrix estimation is therefore a central problem in high-dimensional statistics [43; 64; 65], commonly addressed through regularized fitting methods, most notably *ℓ*_1_- and *ℓ*_2_-penalized regression such as Lasso [66], ridge regression [67], and the elastic net [68; 69], which underlie many of the precision matrix estimation methods benchmarked in the analyses presented here (Table 1).

**Table 1:** Overview over the used precision matrix estimation methods

Abbreviation	Name	Publication	Year	Version	Internal PT
glasso	Graphical Lasso	[51]	2008	1.11	-
CLIME	Constrained *ℓ*_1_-Minimization for Inverse Matrix Estimation	[90]	2011	0.5.0	+
TIGER	Tuning-Insensitive Graph Estimation and Regression	[71]	2012	(flare 1.8)	+
SCIO	Sparse Column-wise Inverse Operator	[91; 105]	2012	0.9.0	+
GLASSOFAST	Graphical Lasso Fast	[106]	2012	1.0.1	-
QUIC	Quadratic Approximate Inverse Covariance	[55; 95]	2013	1.1.1	-
BIGQUIC	Big Quadratic Approximate Inverse Covariance	[107]	2013	1.1-13	+
fastclime	Fast Constrained *ℓ*_1_-Minimization for Inverse Matrix Estimation	[72]	2014	1.4.1.1	-
ROPE	Ridge-type Operator for the Precision matrix Estimation	[108]	2017	-	-
SQUIC	Sparse Quadratic Approximate Inverse Covariance	[18; 63]	2019	1.0	-
EQUAL	Efficient ADMM algorithm via the QUAdratic Loss	[92]	2020	1.2	+
GLassoElnetFast	Graphical Elastic Net	[93]	2021	1.1.0	+
GDTrace	Generalized D-Trace estimator	[94]	2022	-	-
rags2ridges	rags2ridges	[109]	2022	2.2.7	+

Internal PT: Internal Parameter-Tuning. Methods marked “+" perform parameter tuning internally; for methods marked “-", the optimal regularization parameter was selected via K-fold cross-validation to ensure comparability across methods, with the exception of *fastclime*, for which an existing cross-validation implementation using a different selection criterion was used.

## Methods

### Data description

**Fig. 1:**
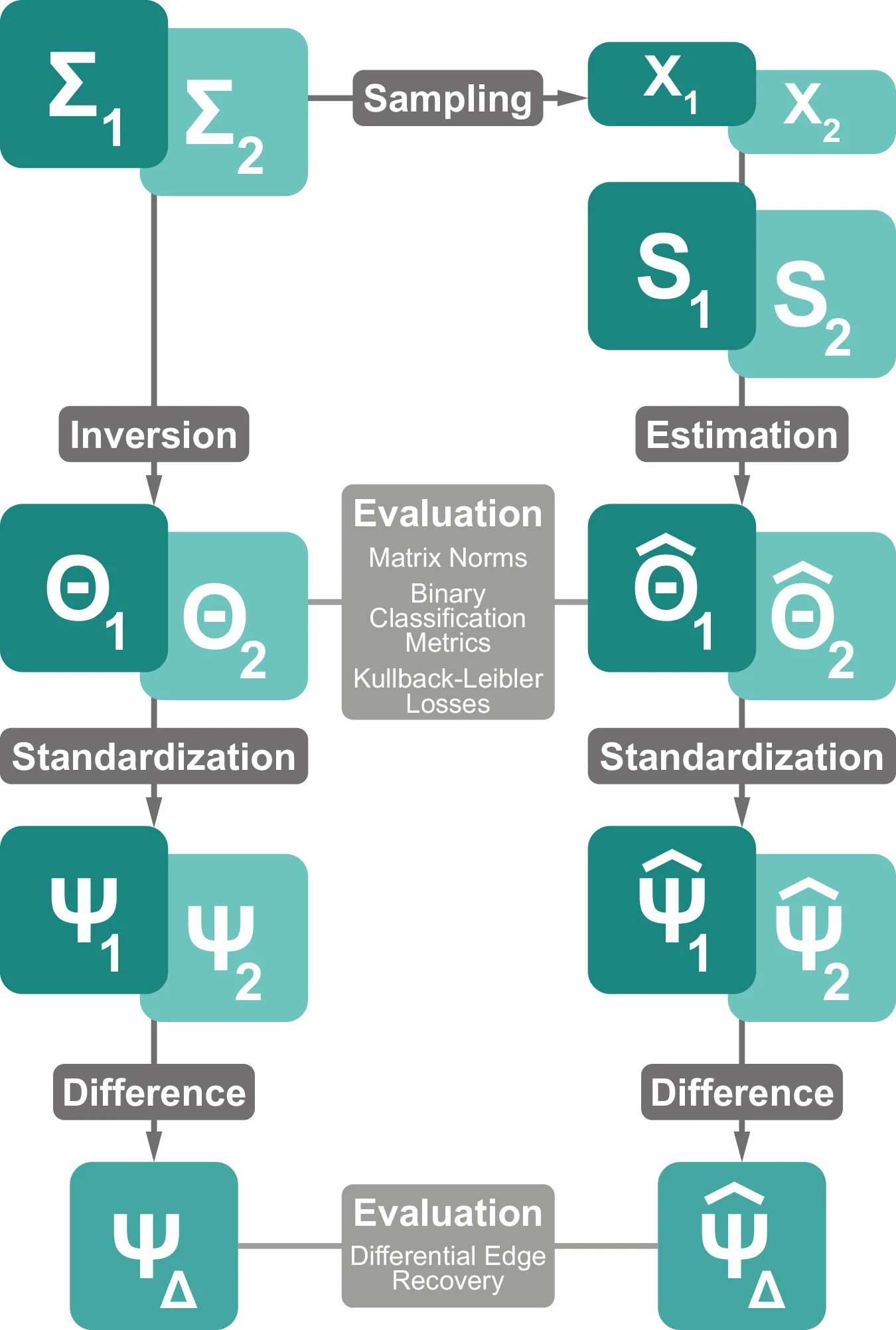
Flowchart, illustrating the general procedure of the simulation studies. Σ: covariance matrix, Θ: precision matrix, **X**: simulated data set, S: sample covariance matrix, $\widehat{\Theta }$: estimated precision matrix, Ψ: partial correlation matrix, $\widehat{\Psi }$: estimated partial correlation matrix, Ψ_*Δ*_: differential partial correlation matrix, ${\widehat{\Psi }}_{\Delta }$: estimated differential partial correlation matrix.

In this study, datasets were simulated to allow evaluation against a known ground truth, in order to provide a reliable benchmark for comparing precision matrix estimation methods. The simulation design follows the intuition of differential gene co-expression. Specifically, two conditions were generated with the same marginal distributions of variables but differing in their underlying correlation structures. In this way, variation between conditions was not reflected in mean differences but in the structure of the condition-specific networks. This setup provides a suitable setting for assessing whether precision matrix estimation methods can recover the predefined structures and, more generally, whether they are suitable to infer partial correlations for differential co-expression analysis. In addition, the simulation design allows systematic variation of key data characteristics, including sample size, dimensionality, covariance structure, and sampling distribution. These factors are used in the subsequent analyses to examine how data properties influence the performance of different estimation methods. Accordingly, the simulation pipeline is designed to generate condition-specific covariance structures, simulate data, and evaluate estimation performance (see Fig. 1 for an overview). In a single simulation run the simulation pipeline starts by generating a covariance matrix for the first condition Σ_1_, using a desired dimension *p* and one of the following covariance 1 methods: single block, multiple block, band network, scale free 1, scale free 2, single block icf, multiple block icf, random graph icf, and random hubs icf (see Covariance Matrix Generation). Furthermore, the methods: scale free 2, single block icf, multiple block icf, random graph icf, and random hubs icf were developed to generate the covariance matrix for the second condition Σ_2_ internally. Otherwise, Σ_2_ is created based on Σ_1_ using one of the two covariance 2 methods: *knockout* and *mutate* (see Covariance Alteration). Σ_1_ and Σ_2_ are generated to be positive definite. Thus, the inverse covariance matrices, ${\Sigma }_{1}^{-1}={\Theta }_{1}$ and ${\Sigma }_{2}^{-1}={\Theta }_{2}$, can be computed and used as ground truth in the subsequent analysis. Additionally, the precision matrices are used to calculate the differential partial correlation matrix Ψ_Δ_. Furthermore, Σ_1_ and Σ_2_ are used to generate the simulated gene expression data sets **X**_1_ and **X**_2_ with a specified sample count *n* (see Sampling). These data sets reflect the respective covariance structure between the variables. Either a multivariate normal, negative binomial, or Poisson distribution of the individual variable counts is simulated. The sample covariance matrices S_1_and S_2_ are calculated based on the simulated data sets. **X**_1_ and **X**_2_ as well as S_1_and S_2_ are used as input for the precision matrix estimation methods (Table 1). These estimate the precision matrices for both conditions ${\widehat{\Theta }}_{1}$ and ${\widehat{\Theta }}_{2}$. The performance of the PMEMs is evaluated by comparing the respective ${\widehat{\Theta }}_{1}$ and ${\widehat{\Theta }}_{2}$ with Θ_1_and Θ_2_ (see Evaluation). Additionally, the estimated differential partial correlation matrix ${\widehat{\Psi }}_{\Delta }$ is compared with Ψ_Δ_.

### Covariance matrix generation

We define the density of a matrix ${\bf{A}}\in {{\mathbb{R}}}^{p\times p}$ by the proportion of non-zero entries in **A**

$${\rm{dens}}({\bf{A}})=\dfrac{1}{{p}^{2}}\mathop{\sum }\limits_{j=1}^{p}\mathop{\sum }\limits_{k=1}^{p}{\bf{1}}\left\{{A}_{jk}\ne 0\right\}.$$

#### Block covariance structures

The *single block* covariance method was developed based on the simulation approaches from Kuismin and Sillanpää (2017) and Fop et al. (2017) [64; 70]. With the standard settings, this approach generates a symmetric 100 × 100 matrix with one 50 × 50 sized non-zero cluster. All non-zero off-diagonal values (covariances) within this cluster are set to 1, while all diagonal values (variances) of the entire matrix are set to 2 (Figure S1A). The specified settings result in a positive definite covariance matrix with a density of 0.255. The *multiple block* covariance structure is based on the fourth simulation setting from Fop et al. (2017) [70]. The default settings result in the generation of a symmetric 100 × 100 matrix comprising ten clusters, each of size *p*/10. Similar to the single block covariance matrix, all non-zero off-diagonal values within these clusters are set to 1, while all diagonal values of the entire matrix are set to 2 (Figure S1B). The aforementioned settings result in a positive definite covariance matrix with a density of 0.1. The *band network* structure approach is based on the covariance and precision matrix generation from Liu and Wang (2012) as well as Pang et al. (2014) [71; 72]. Both utilize the band network structure to directly generate a dense precision matrix. In contrast, the band network structure is used in this work to generate a sparse covariance matrix with a density of 0.0494. Using the standard settings, the matrix is generated with diagonal values of 2. The first off-diagonal values are set to 1, and the second off-diagonal values are set to be 0.5 times the values of the first off-diagonal (Figure S1C). This guarantees a positive definite matrix.

#### Scale free network structures

The *scale free 1* method aims to generate a scale free network, in which the distribution of links to nodes follows a power law. For this purpose,​ a network is generated using the generate_BA() function from the R package **PAFit** (version: 1.2.11, [73]). The parameter num_seed is set to 4, while the multiple_node parameter is set to 2. This function generates networks from the generalized Barabási-Albert model [26]. In this model, the preferential attachment function is power-law and node fitnesses are all equal to 1 (Figure S2). The generated network’s adjacency matrix is used as covariance matrix where non-zero off-diagonal values are set to 1 and diagonal values are set to 2 (Figure S3). Since the covariance matrix generated in this way is not positive definite, the function nearPD() from the R Package **Matrix** (version: 1.7-6, [74]) was used to find the nearest positive definite matrix. While this approach preserves the underlying links in the network and guarantees a positive definite matrix, all edges are changed in their weighting. As a result, the matrix also loses its sparse property.

The *scale free 2* method, developed by Zhao and Shojaie (2022) to simulate changes in biological networks for their qualitative hypothesis testing framework [75], generates the edge set *E*_1_ from a power-law distribution (power parameter 5) using sample_fitness_pl() from the R package igraph (version 2.3.3, [76]), with ∣*E*_1_∣ = *p*(*p* − 1)/100 corresponding to an edge density of 0.02. For *p* = 200, *E*_2_ is obtained by removing all edges connected to 20 randomly selected nodes among the 100 most connected nodes in *G*_1_, then adding random edges back until ∣*E*_2_∣ = ∣*E*_1_∣. Edge weights for Θ_1_ and Θ_2_ are assigned at random ( ± 0.5 with equal probability), with shared edges in *E*_1_ ∩ *E*_2_ retaining the same weight in both matrices. Diagonal entries are set to ensure a minimum eigenvalue of 0.1, and the resulting precision matrices are normalized so that the corresponding covariance matrices have unit variances. The full construction, including the case-by-case definitions of Θ_1_ and Θ_2_, is provided in the Supplementary Information (Section 1.1.1). Examples of the generated networks and covariance matrices are shown in Supplementary Figures 4 and 5.

#### Iterative conditional fitting algorithm based structures

The following methods: *single block icf*, *multiple block icf*, *random graph icf*, and *random hubs icf* are based on Fop et al. (2017) [70] and the R package mixggm (version: 1.0, [77]). The included fitGGM() function is able to estimate a graph structure constrained GGM using the Iterative Conditional Fitting (ICF) algorithm by Chaudhuri et al. (2007) [78], ultimately yielding a positive definite covariance matrix. This approach was incorporated like the other covariance generation methods, by providing two specified adjacency matrices and a simulated mixture of *K* = 3 multivariate Gaussian distributions with mixing proportions of *τ* = (0.2, 0.5, 0.3). The means of the Gaussian distributions were randomly selected within specific ranges: (-1, 1), (-2, 2), and (-3, 3). In the *single block icf* and *multiple block icf* methods the first adjacency matrices are generated using the previously described *single block* or *multiple block* method, respectively. In the *random graph icf* method random graphs are generated according to the G(n,p) Erdős-Rényi model [79] using the sample_gnp() function from the R package igraph and used as the first adjacency matrix. For the *random hubs icf* method the first adjacency matrix is based on a hub structure generated by the HubNetwork() function from the R package hglasso (version: 1.0, [80]). For all these methods, the second adjacency matrix is generated using the covariance alteration method knockout. The final covariance matrices for both conditions were generated using the respective adjacency matrices and the same simulated data set (Figures S6, S7, S8, and S9).

#### Covariance alteration

In differential network analysis, the performance of precision matrix estimation methods depends on their ability to detect differences between networks across conditions. Accordingly, when simulating data from covariance matrices, these differences are introduced directly at the level of the condition-specific covariance structures. Therefore, a second covariance matrix Σ_2_ with predefined edge-level differences relative to the first condition, based on Σ_1_, is required. To introduce controlled differences between conditions, two covariance alteration strategies were considered: *knockout* and *mutate*.

The *knockout* strategy is motivated by biological scenarios in which mutations in regulatory genes lead to the loss of downstream interactions, and is used here to model the removal of network connections between conditions. Specifically, it reflects scenarios in which mutations in a set of genes lead to structural alterations in corresponding regulatory hub proteins. These alterations affect the protein’s binding properties, ultimately resulting in a loss of regulatory influence and co-expression with downstream genes. Technically, *knockout* is implemented by deleting all connections of selected nodes in Σ_2_, modifying the correlation structure without modifying the marginal distributions (Figure S10A). In line with the biological intuition, this means that the expression levels and variances of the affected genes are preserved across conditions. Analogously, the *mutate* strategy is motivated by biological scenarios involving both loss- and gain-of-function effects and is used to model simultaneous removal and introduction of network interactions between conditions. Thus, it simulates scenarios in which mutations in regulatory hub genes lead to structural changes in the corresponding proteins, resulting in both the removal of existing interactions and the formation of new ones with downstream targets. Technically, this is implemented by deleting selected edges and introducing novel connections in Σ_2_, thereby altering the correlation structure between conditions. To ensure positive definiteness of Σ_2_, the variances of the affected genes are also doubled (Figure S10B).

### Sampling

A total of six methods were used to generate data sets for the respective simulation approaches. With a defined number of samples *n* and a given covariance matrix (Σ_1_ or Σ_2_) of the dimension *p* × *p*, these methods generate random samples from a multivariate Gaussian, Poisson, or negative binomial distribution, thus creating a data set (X) with the dimension *n* × *p* for each condition. In this step the high-dimensional characteristics and noise of real data sets are simulated by always selecting *p*≥*n*. In the majority of the analyses conducted in this study, the mvrnorm function was utilized to generate random samples from a multivariate normal distribution. This function is based on Ripley (1987) [81] and is available in the R package MASS (version: 7.3-66, [82]). An example for S can be seen in Supplementary Figure 11. Similarly, the function rmvnorm() from the R package SimDesign (version: 2.27, [83; 84]) was used to generate random samples based on a normal distribution. For both of these methods, the mean value of every variable in generated data sets was set by default to zero. Alternatively, another mean value or range can be set. In addition to drawing samples from a multivariate normal (Gaussian) distribution, a Poisson distribution was used. Three methods were utilized for this, which in the following are referred to as PoisNor, PoisBinOrdNonNor, and Poisson. PoisNor is based on the function genPoisNor() included in the R package PoisNor (version: 1.3.3, [85]). PoisBinOrdNonNor is based on the function genPBONN() from the R package PoisBinOrdNonNor (version: 1.5.3, [86]). These Poisson distribution generation methods rely on a provided correlation matrix. For this, the previously generated covariance matrix was transformed into a correlation matrix using the cov2cor() function from the base R package stats [87]. Furthermore, all Poisson distribution-based sample generation methods used a rate parameter (*λ* = 0.9) for all Poisson variables. The sample generation method, previously introduced as Poisson, was developed by Yahav and Shmueli (2007) [88]. A detailed description of the algorithm and implementation is provided in the Supplementary Information (Section 11). To account for overdispersion commonly observed in RNA-seq count data, which the Poisson distribution’s equal mean-variance assumption cannot capture, an additional sampling method, referred to as NegBin, was implemented. This method extends the Gaussian-copula approach described for the Poisson method to the negative binomial distribution, following the general framework of Cario and Nelson (1997) [89] for generating random vectors with arbitrary marginal distributions and a specified correlation matrix. As with the Poisson-based methods, the previously generated covariance matrix was transformed into a correlation matrix using cov2cor(). In addition to the correlation matrix, NegBin requires a mean (*μ*) and dispersion (*size*) parameter for each variable, with variance given by *Var*(*X*) = *μ* + *μ*^2^/size; smaller values of *size* correspond to greater overdispersion, and as *size* → *∞* the negative binomial distribution converges to the Poisson distribution. For the analyses conducted here, *μ* = 10 and size = 10 were used. The algorithm and implementation is provided in the Supplementary Information (Section 11).

#### Normalization

Additionally after sampling, two optional normalization strategies can be applied to the generated data sets. In the first approach, variables are standardized to have both zero mean and unit variance, thereby removing differences in scale across features. In the second approach, variables are mean-centered only, retaining their original variances.

### Precision matrix estimation methods

A list of all methods included in the analysis is provided in Table 1. Methods were considered for inclusion if they (i) directly estimate the precision matrix, (ii) were described in a publication or cited in related work, (iii) provided an implementation in R as a package or through publicly available source code, and (iv) were released after *glasso* and *clime*, which have become widely used reference methods in the field. Faster implementations of earlier approaches were also included. Methods were excluded if they lacked sufficient documentation to be executed or if no working implementation was available.

#### Cross-validation

Since PMEMs differ in whether they provide an internal parameter-tuning procedure (see Table 1, column “Internal PT"), a consistent tuning approach was needed across all methods. For methods with internal PT, the optimal regularization parameter could be determined automatically as part of their implementation as an R package, whereas for all remaining methods it was selected via K-fold cross-validation as described below, ensuring comparable optimization conditions across both groups. A K-fold cross-validation procedure was used to select the optimal regularization parameter for *glasso*, *quic*, *gdtrace*, *rope*, *glassofast*, and *squic*. The simulated data set (with *n* samples and *p* variables) was split into K disjoint folds of approximately equal size, with each observation appearing in the validation set exactly once and the remaining K-1 folds serving as the training set. Using the training data, the sample covariance matrix (S_*train*_) was computed and a precision matrix (Θ_*train*_) estimated. The model was then validated on the corresponding test data by computing the test-set sample covariance matrix (S_*test*_) and evaluating performance via the negative log-likelihood

3 $${\mathcal{L}}({\Theta }_{train},{S}_{test})=-\log \det ({\Theta }_{train})+\,\mathrm{tr}\,({S}_{test}\cdot {\Theta }_{train}), $$

where Θ_*train*_ denotes the precision matrix estimated from the training data and S_*test*_ the empirical covariance matrix of the test set. For each value of the regularization parameter (*ρ*, *α*), the negative log-likelihood scores were averaged across the K folds to produce a cross-validation score. This was repeated across the full parameter list, and the value minimizing the average score was selected as the optimal regularization parameter (*ρ*_*opt*_, *α*_*opt*_). For *fastclime*, which also lacks internal parameter tuning, the regularization parameter was instead selected using an existing K-fold cross-validation implementation https://github.com/meca7653/HDtest-2018-8/blob/master/R/cv_fastclime.R that minimizes the Frobenius norm between the estimated precision matrix and the inverse test-set covariance matrix, rather than the negative log-likelihood used for the other methods.

### Evaluation

To evaluate the performance of the precision matrix estimation methods, a set of established metrics was used, which are briefly summarized below and described in detail in the following subsections. These include matrix norms, specifically the matrix 1-norm, Frobenius norm, and spectral norm, as applied in previous studies [64; 71; 90–94]. Kullback-Leibler losses (KLL and reverse KLL) were also considered, consistent with their use in [64; 93; 94]. Binary classification metrics, namely F1 Score, Accuracy, and Matthews Correlation Coefficient (MCC), were used to summarize overall classification performance in identifying non-zero entries of the precision matrix, corresponding to the presence or absence of edges in the inferred network [63; 90; 93]. Additionally, a differential edge recovery metric was used to capture graph reconstruction performance, similar to approaches in [93]. In this study, the binary classification metrics (F1 Score, Accuracy, and MCC) are used to concisely summarize PMEM performance. However, the underlying evaluation pipeline also supports more detailed assessments using receiver operating characteristic (ROC) curves and Precision-Recall curves, enabling the analysis of sensitivity, specificity, precision, and recall. These metrics are likewise reported in related works [71; 90; 91; 95]. The ROC and precision-recall curves were not used in this work because they would need to be generated for every precision matrix estimation method in every simulation condition, resulting in a large number of curves that are difficult to summarize and compare. Instead, the aforementioned binary classification metrics were used to provide an interpretable summary of overall performance. ROC and precision-recall curves are nevertheless useful for detailed method-specific analyses. Two examples are shown in Figures S12 and S13.

#### Matrix norms

The following matrix norms quantify the discrepancy between the true precision matrices Θ_1,2_ and the estimated precision matrices ${\widehat{\Theta }}_{1,2}$, based on the difference matrix $\Delta =\widehat{\Theta }-\Theta$. Lower values indicate more accurate estimation. The matrix 1-norm, Frobenius norm, and spectral norm are defined as

4 $$\begin{array}{l}\parallel A{\parallel }_{1}={\mathop{\max }\limits_{1\le j\le p}}\mathop{\sum }\limits_{i=1}^{p}| {a}_{ij}| ,\quad \parallel A{\parallel }_{F}=\sqrt{{\rm{tr}}(A{A}^{\top })},\quad \parallel A{\parallel }_{2}={\sigma }_{\max }(A),\end{array} $$

respectively, where ${\sigma }_{\max }(A)$ denotes the largest singular value of *A *[96]. In addition, estimation accuracy was evaluated using the Kullback-Leibler Loss (KLL) and Reverse KLL (RKLL),

5 $$\,\mathrm{KLL}\,(\widehat{\Theta },\Theta )=\,\mathrm{tr}\,({\Theta }^{-1}\widehat{\Theta })-\log \det ({\Theta }^{-1}\widehat{\Theta })-p,$$

6 $$\,\mathrm{RKLL}\,(\widehat{\Theta },\Theta )=\,\mathrm{tr}\,(\Theta {\widehat{\Theta }}^{-1})-\log \det (\Theta {\widehat{\Theta }}^{-1})-p,$$

where *p* is the matrix dimension. Both are minimized when $\widehat{\Theta }=\Theta$, with lower values indicating more accurate estimation.

#### Binary classification metrics

Performance in recovering the underlying graph structure was further assessed using standard binary classification metrics: F1 score, accuracy, and the Matthews correlation coefficient (MCC), as defined in [97; 98]. All three are computed from the entries of the confusion matrix (true/false positives and negatives, based on agreement between estimated and true edges). To improve interpretability, a normalized version of the MCC (normMCC), rescaled to [0, 1] with 0 corresponding to the worst and 1 to the best performance, is reported throughout this study, with undefined cases handled following the convention of Chicco and Jurman (2020) [99]. Full definitions, formulas, and worked examples are provided in the Supplementary Information (Section 1.4.1).

#### Differential edge recovery

To overcome the limitations of binary classification metrics, which are highly sensitive to the density of either the estimated or the true precision matrix, and of matrix norms, which do not directly assess edge recovery, we introduced a differential edge recovery (DER) metric. This metric evaluates how accurately the PMEMs capture the true structural differences between the partial correlation networks of the two simulated conditions. In addition, it provides a thresholding mechanism for PMEMs that do not inherently yield sparse matrices.

The idea of the DER score is as follows. From the ground-truth differential partial correlation matrix Ψ_Δ_, only the important differential edges are considered. Non-edges, i.e. pairs with ${\psi }_{ij}^{\Delta }=0$, are excluded by construction, giving the true edge set

$$E({\Psi }_{\Delta })=\{(i,j):\,{\psi }_{ij}^{\Delta }\ne 0,\ i < j\}.$$

Among these, less relevant edges with small absolute weights are further excluded by applying a weight threshold *τ* ≥ 0, which defines the filtered edge set

$${E}_{{\Psi }_{\Delta }}^{\star }(\tau )=\{(i,j)\in E({\Psi }_{\Delta }):\,| {\psi }_{ij}^{\Delta }| \ge \tau \}.$$

Let $k=| {E}_{{\Psi }_{\Delta }}^{\star }(\tau )|$ be the number of important ground-truth edges. From the estimated differential partial correlation matrix ${\widehat{\Psi }}_{\Delta }$, the *k* edges with the largest absolute weights are selected to form the set ${E}_{{\widehat{\Psi }}_{\Delta }}^{\,\mathrm{top}\,}$. The DER score is then defined as

7 $$\,\mathrm{DER}\,=\dfrac{| {E}_{{\widehat{\Psi }}_{\Delta }}^{\,\mathrm{top}\,}\cap {E}_{{\Psi }_{\Delta }}^{\star }(\tau )| }{| {E}_{{\Psi }_{\Delta }}^{\star }(\tau )| }\times 100 \% ,$$

where ∣ ⋅ ∣ denotes set cardinality. The DER score ranges from 0% (no correct recovery) to 100% (perfect recovery). The DER score depends on the choice of *τ*, which determines the number and identity of ground-truth edges considered relevant. Rather than fixing *τ* to a single value, we generated a grid of *m* threshold values spanning the range of ground-truth differential edge weights,

$${\tau }_{i}={\tau }_{\max }{\left(\dfrac{i-1}{m-1}\right)}^{\gamma },\qquad i=1,\ldots ,m,$$

with *γ* > 1 and ${\tau }_{\max }={\mathop{\max }\limits_{i < j}}| {\psi }_{ij}^{\Delta }|$ the largest absolute edge weight in the ground-truth differential partial correlation matrix Ψ_Δ_ of the respective simulated network. This power-law spacing samples *τ* more densely near zero, where small changes in *τ* have the largest effect on the composition of ${E}_{{\Psi }_{\Delta }}^{\star }(\tau )$, and more coarsely as *τ* approaches ${\tau }_{\max }$, where ${E}_{{\Psi }_{\Delta }}^{\star }(\tau )$ changes more gradually. We used *m* = 10 and *γ* = 2. The sensitivity of DER to *τ* is evaluated for all PMEMs in each of the subsequent analyses, which vary the covariance values, the dimension, and the sample size.

The DER score is conceptually related to recall at *k*, since it quantifies the proportion of relevant items (important ground-truth differential edges) recovered among the top-*k* ranked estimated edges. Unlike a conventional recall@*k* evaluation, however, *k* is not fixed a priori but derived directly from the thresholded ground-truth network itself, $k=| {E}_{{\Psi }_{\Delta }}^{\star }(\tau )|$. Because the retrieved and relevant sets are therefore of equal size, DER simultaneously corresponds to recall, precision, and *F*_1_ at *k*, making it a single, threshold-adaptive summary of structural recovery rather than an arbitrarily parameterized retrieval metric.

#### Runtime and memory tracking

Estimation runtime was tracked for the complete estimation step of every PMEM. For each condition’s precision matrix, this included the respective parameter tuning step, followed by estimation of matrices across the list of regularization parameters, either predefined or determined by the tuning procedure. The time for these steps was recorded for both conditions and summed to yield the estimation runtime for the respective method.

Peak memory usage was tracked for each PMEM call using two approaches. The standard approach polled the resident memory of the continuously running R session at 10 ms intervals via a background process (**ps** and **callr** R packages) and recorded the maximum value. The isolated approach involved executing each call in a newly spawned, independent R process, polling for peak memory for that process alone. Both track resident set size (RSS) rather than R’s own memory accounting, since some of the PMEMs rely on compiled C/C++ backends allocating memory outside R’s memory manager. To assess whether tracking itself affected estimation runtimes, a subset of replicates was additionally run without any memory tracking.

### Real-world data application

To assess the practical relevance of the benchmarked precision matrix estimation methods beyond simulation, we apply a subset of estimators to a real RNA-seq dataset. Raw gene counts for GSE156063 [100] are downloaded from the Gene Expression Omnibus (GEO). The dataset comprises upper airway swab samples from patients with COVID-19 (SC2, *n* = 93), non-viral acute respiratory illness (no virus, *n* = 100), and other-viral ARIs (*n* = 41). We restrict the analysis to the SC2 and no-virus groups, since both cohorts are symptomatic. Counts are filtered for expression using filterByExpr() (edgeR) and variance-stabilized with DESeq2’s vst, blind to condition. Sex effects are regressed out removeBatchEffect (limma) prior to gene selection. The top *p* = 1000 genes are retained by pooled within-group variance and expression values are standardized within each condition. The following estimators were chosen to provide a variety of options that span the sparsity spectrum: *glassofast*, *GLassoElnetFast*, *equal*, and *rags2ridges*. Regularization parameters are tuned separately per condition. Each estimator returns a precision matrix per condition, converted to a partial correlation matrix. A differential edge is defined, per method, as one of the top *k* = 200 gene pairs ranked by absolute difference in partial correlation between conditions. Cross-method agreement is assessed via the Jaccard index over these differential edge sets.

As no ground-truth network exists for real transcriptomic data, this application demonstrates practical applicability and estimator-dependent variability in recovered (differential) networks, rather than validating estimator accuracy.

## Results

Table 2 provides an overview of all analyses performed in this study. Only the settings highlighted in bold in Table 2 are discussed in detail in the following sections. Results for all other settings are provided in the Supplementary Data. A baseline scenario was defined using block-structured covariance matrices, with differences between conditions introduced at the level of the condition-specific covariance matrices through the knockout procedure (see Methods: Covariance Alteration). The dimensionality was set to *p* = 100 and the sample size to *n* = 50, with data sampled from a multivariate Gaussian distribution with a zero mean vector. Subsequent analyses systematically varied one parameter of this baseline scenario at a time, in order to assess the influence of individual data properties on the performance of precision matrix estimation methods. Further detailed figures and additional results can be found in the Supplementary Data. The analyses are presented in the following order: covariance structures, covariance densities, covariance values, dimensionality, sample size, sampling distributions, and runtimes.

**Table 2:** Overview of the analyzed simulation settings.

Approach	p	n	Sampling	Covariance Generation	Covariance Alteration	Means	Normalization	Replicates	Additional Variation
Covariance Alteration	100	50	Gaussian	multiple block	knockout, mutate	0	0	10	
**Covariance** **Structures**	100	50	Gaussian	single block, multiple block, band network, scale free 1, scale free 2, single block icf, multiple block icf, random graph icf, random hubs icf	knockout	0	0	-	
**Covariances**	100	50	Gaussian	multiple block	knockout	0	0	-	*
​**Densities**	100	50	Gaussian	multiple block	knockout	0	0	-	**
**Dimensions**	100, 200, 500, 1000, 2000	50	Gaussian	multiple block	knockout	0	0	-	
Mean Distribution	100	50	Gaussian	multiple block	knockout	0, 10, 5-10	0	10	
Normalization	100	50	Gaussian	multiple block	knockout	5-10	0, 1, 2	10	
Replicates	100	50	Gaussian	single block	knockout	0	0	50	
**Sample Size**	100	20, 25, ... , 100	Gaussian	multiple block	knockout	0	0	-	
**Sampling** **Distributions**	100, 200, 500	50	Gaussian, Poisson, Negative Binomial	multiple block	knockout	0	0	-	

Approaches highlighted in bold are described in detail in the following sections, while results for all remaining approaches are provided in the Supplementary Data. *p* denotes the dimensionality and *n* the sample size. Sampling indicates the distribution used for data generation. Covariance Generation specifies the methods used to construct Σ_1_, while Covariance Alteration describes how Σ_2_ is derived from Σ_1_. Means indicate the mean vectors used for sampling from Σ_1_ and Σ_2_. Normalization specifies whether no normalization (0), normalization of means and variances (1), or normalization of means only (2) was applied. Replicates denote the number of simulation replicates. Additional variation indicates settings with modified covariance structures: * indicates settings in which the off-diagonal entries were fixed to *c* ∈ {1, …, 10} and the diagonal entries to *c* + 1 ∈ {2, …, 11}, with *c* varied across settings; ** corresponds to *k* non-zero blocks in Σ_1,2_ with *k* ∈ {3, …, 20}, resulting in precision matrix densities ranging from 0.05 to 0.33.

### Covariance structures

To test whether the underlying covariance structures of the simulated datasets influence the performance of PMEMs, nine covariance generation methods, or covariance 1 methods, were developed and tested, namely single block, multiple block, band network, scale free 1, scale free 2, single block icf, multiple block icf, random graph icf and random hubs icf (see Methods: Covariance Matrix Generation). The results are presented in the Supplementary Figures 15, 16, and 17. For most PMEMs, performance across all three metrics varies considerably depending on the covariance generation method used. In the binary classification metrics, the PMEMs *bigquic*, *scio*, and *tiger* show similar results (Figure S15). For almost all PMEMs a bad performance in band network, scale free 1, random graph icf, and random hubs icf approaches can be seen. *Clime*, *rags2ridges*, and *rope* show identical results, and in contrast to the other PMEMs result in high F1 score and accuracy in the band network, scale free 1, random graph icf, and random hubs icf approaches. However, the normalized MCC reveals that all PMEMs perform equally poorly in these approaches, with values consistently at 0.5 across all methods. These patterns highlight that the structure induced by different covariance 1 methods can significantly affect binary classification metrics.

In general, the selected covariance generation methods led to different values of the matrix norms for each PMEM (Figure S16). Most notably, all analyzed PMEMs showed the highest values for the matrix 1-norm, Frobenius norm, and spectral norm in the approaches based on the scale free 1 method, consistently several orders of magnitude higher than in the other settings. Since all methods were affected, the explanation lies not in the estimation methods themselves but in the generation of the ground truth matrices Σ_1_ and Θ_1_. The scale free 1 method uses the nearPD() function to enforce positive definiteness, but the resulting Σ_1_ remained nearly singular, with eigenvalues close to zero. As a consequence, the inverse matrix Θ_1_ contained extremely large entries. In contrast, the PMEMs estimated precision matrices with more typical magnitudes, resulting in consistently large differences between the estimated precision matrix ${\widehat{\Theta }}_{1}$ and the ground truth precision matrix Θ_1_, and thereby inflating the matrix norm values across all methods. Apart from this, the Σ_1_ generation method scale free 2 results in the highest values of the Frobenius norm for all PMEMs. The lowest values for the Frobenius norm are observed in approaches based on the single block or multiple block method. The highest matrix 1-norms are seen in approaches based on scale free 2, single block icf, and random hubs icf. Overall, a significant influence of the selected covariance 1 methods on the performance of the PMEMs can be seen on the basis of the presented matrix norms. In this context, the PMEMs display comparable performance trends across settings. This suggests a similar response to differences in the underlying covariance structures.

### Covariance densities

In the previous subsection, the influence of different covariance structures on PMEM performance was examined. However, the methods used to generate Σ_1_, and consequently Θ_1_, differ not only in their non-zero patterns but also in the resulting matrix densities (see Methods: Covariance Matrix Generation). In the approaches considered, the single block and multiple block methods yielded Θ_1_ densities of 0.12 and 0.10, respectively. The band network, random graph icf, and random hubs icf methods resulted in matrices with a density of 1. The scale free 1 and scale free 2 approaches produced densities of 0.98 and 0.55, respectively. The single block icf had a density of 0.26, while the multiple block icf matched the standard multiple block method with a density of 0.10.

These differences highlight the strong dependence of PMEM performance, as measured by binary classification metrics, on the density of the underlying precision matrix. Accordingly, this section further investigates how variation in the density of the true precision matrix Θ_1_ affects the performance of different PMEMs. The density of Θ_1_ was systematically increased from 0.05 to 0.33 by adjusting the number of non-zero blocks generated using the multiple block covariance method (Figure S18). Since the resulting precision matrices derived from this method consistently match the density of their corresponding covariance matrices, only the density of the true and estimated precision matrices is considered in the following analysis. An important property of the sample covariance matrix S is that, regardless of the density of the underlying true covariance matrix or the number of samples, S is always fully dense. Thus, PMEMs receive no explicit information about the true sparsity of the underlying structure. They must infer it entirely through their internal regularization, based solely on the signal present in S.

**Fig. 2:**
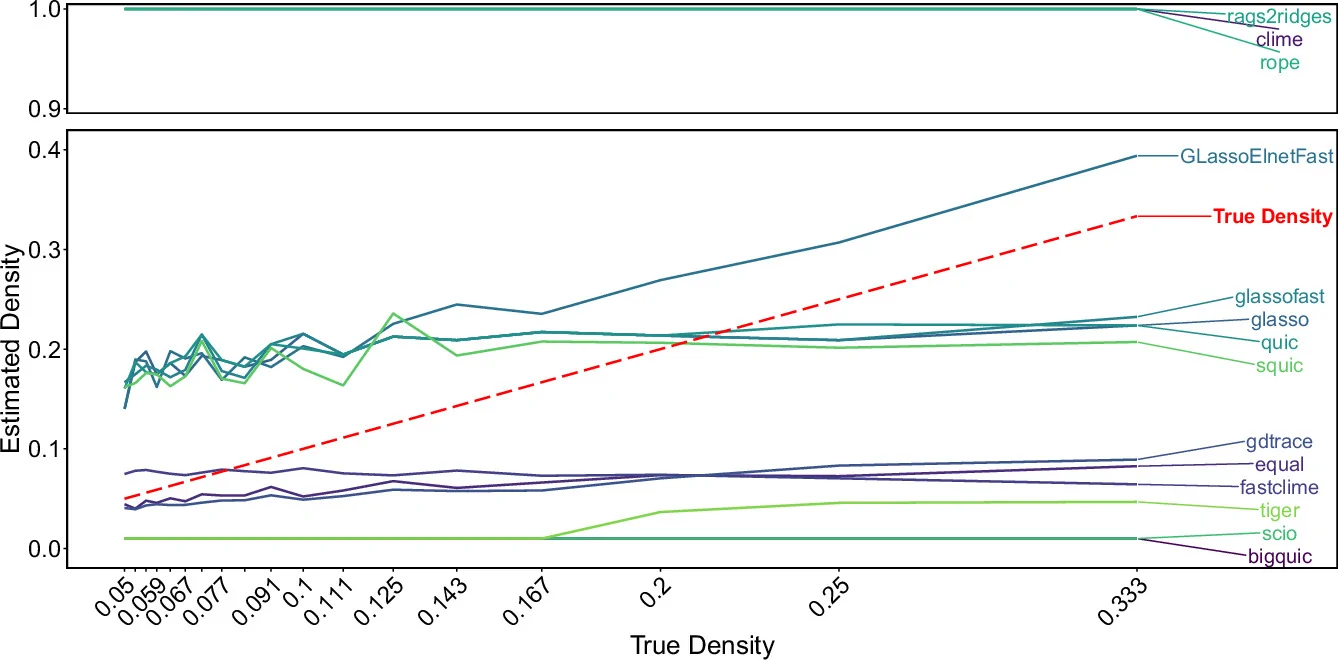
Line plot illustrating the influence of varying true densities of Θ_1_ on the densities of the estimated precision matrices for condition one (${\widehat{\Theta }}_{1}$). The PMEMs *clime*, *rags2ridges*, and *rope* consistently estimated the matrices with a density of 1 across all settings. In contrast, *bigquic*, *scio*, and *tiger* predominantly recovered only diagonal elements, resulting in an estimated density of 0.01. For densities greater than 0.2 in Θ_1_, *tiger* showed a slight increase in density in ${\widehat{\Theta }}_{1}$. *Gdtrace* and *equal* exhibited similar behavior, with only minimal increases in estimated density as the true density increased, while *fastclime* showed a slight decrease in the density of ${\widehat{\Theta }}_{1}$ with increasing density of Θ_1_. Comparable patterns were observed for *glasso*, *glassofast*, *quic*, and *squic*, which displayed only modest sensitivity to changes in true density, generally overestimating density at low true densities (≤0.2) and underestimating density at higher true densities (0.25 and 0.333). *GLassoElnetFast* consistently overestimated the density of ${\widehat{\Theta }}_{1}$, but was the only PMEM to exhibit a clear and systematic increase in estimated density with increasing true density of Θ_1_.

The binary classification metrics F1 score, accuracy, and normalized MCC declined for most PMEMs as the density of Θ_1_ increased (Figure S19). *GLassoElnetFast* was the exception, showing an increase in F1 score, a smaller decrease in accuracy compared to other PMEMs, and normalized MCC values that increased at lower densities and remained stable at higher ones. *Clime*, *rags2ridges*, and *rope* also showed increased F1 scores and accuracy, though their normalized MCC remained constant at 0.5. The matrix norm values generally increased with density for all methods except *rags2ridges*, which showed decreasing norm values across all density levels (Figure S20). Figure 2 reveals an interesting relationship between the density of $\widehat{\Theta }$ and the true density of Θ_1_. For most PMEMs, the estimated density remained largely unchanged regardless of the ground truth density, suggesting a lack of adaptivity in sparsity estimation. *GLassoElnetFast* was the only method to adjust its estimated density in response to the true structure, though it tended to overshoot.

As a result, binary classification metrics are most meaningfully interpretable for *GLassoElnetFast* in this setting, since performance for the other methods depends strongly on the mismatch between estimated and true density. Finally, it is worth noting that this was the only scenario in which *tiger* did not produce an empty matrix, beginning to yield non-zero estimates at densities of Θ_1_ ≥ 0.2.

### Covariance values

**Fig. 3:**
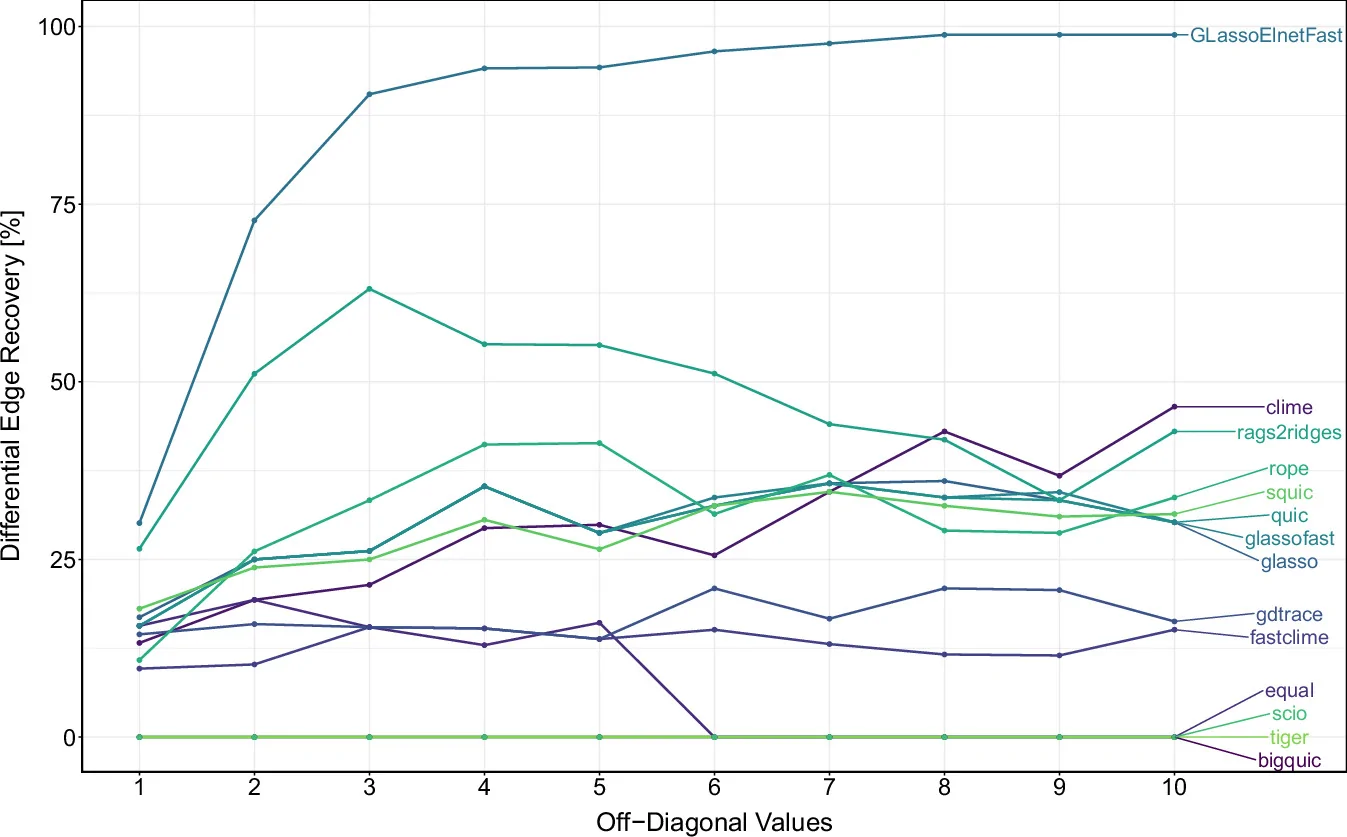
Line plot illustrating the influence of varying off-diagonal entry values (covariances) on the performance of precision matrix estimation methods, assessed by differential edge recovery rates. The PMEMs *scio*, *tiger*, and *bigquic* did not recover any differential edges across the considered settings. For *equal*, differential edge recovery decreased with increasing off-diagonal entry values and dropped to zero for values of 6 and higher. Recovery rates for *gdtrace* and *fastclime* were largely unaffected by variations in off-diagonal entry values. In contrast, *rags2ridges* initially recovered an increasing number of differential edges as off-diagonal entry values increased, followed by a decline in recovery rates for values greater than 3. Comparable increasing trends in differential edge recovery with increasing off-diagonal entry values were observed for *rope*, *squic*, *quic*, *glassofast*, *glasso*, and *clime*. Among all methods, *GLassoElnetFast* demonstrated the most pronounced increase in differential edge recovery as off-diagonal entry values increased.

Additionally, we analyzed whether the values of the covariances in the underlying covariance matrices (Σ_1_ and Σ_2_) have an influence on the performance of the PMEMs. This was approached by varying the off-diagonal values or covariances in Σ_1_ and thus also Σ_2_. In each approach, the diagonal values or variances were always set to the used covariance value plus one. This essentially increased the signal-to-noise ratio, while also increasing the variances (Figure S26). Overall, increasing the covariances had little to no effect on the performance of most analyzed PMEMs in terms of F1 score, accuracy, and normalized MCC. The only notable exception was the PMEM *equal*, which showed a clear decline across all binary classification metrics as covariance values increased (Figure S27). Similarly, the performances of most PMEMs measured by the matrix norms were unaffected by varying covariance values (Figure S28). However, the PMEMs *clime*, *equal*, *fastclime*, *scio* and *tiger* result in higher values for Frobenius norm with an increase in the covariance values. *GLassoElnetFast* is shown to achieve the lowest values for all matrix norms in these settings. Additionally, a visible decrease in all matrix norms can be seen when the covariances increase from 2 to 3. In terms of differential edge recovery (see Methods: Differential Edge Recovery), most PMEMs did not substantially benefit from the increased signal-to-noise ratio associated with higher covariance values (Fig. 3). *GLassoElnetFast* was the method that benefited most from this effect, achieving the highest recovery rates across the tested covariance levels, with performance improving as covariances increased. *Rags2ridges* achieved the second-highest recovery rates overall but showed a decline at higher covariance values. A minimal increase in differential edge recovery was observed for *clime*, *rope*, *glasso*, *squic*, *quic*, and *glassofast*. *Gdtrace* and *fastclime* displayed largely stable recovery rates across all conditions, with only small variations. Again, *bigquic*, *scio*, and *tiger* failed to recover any differential edges. The performance of *equal* declined markedly, with recovery rates dropping to zero. These patterns were largely robust to the choice of the weight threshold *τ* (see Figure S29). Although the recovery rate of *GLassoElnetFast* varied with *τ*, it remained higher than that of the other methods across the tested thresholds. The decline of *rags2ridges* at higher covariance values, as well as the failure of *bigquic*, *scio*, and *tiger* to recover any differential edges, were likewise consistent across all thresholds tested. Threshold choice did, however, determine the direction of the effect for several methods. Recovery rates for *clime*, *equal*, *fastclime*, *gdtrace*, *glasso*, *glassofast*, *quic*, and *squic* were higher at lower thresholds, whereas recovery rates for *GLassoElnetFast*, *rags2ridges*, and *rope* were higher at higher thresholds.

### Dimensions

**Fig. 4:**
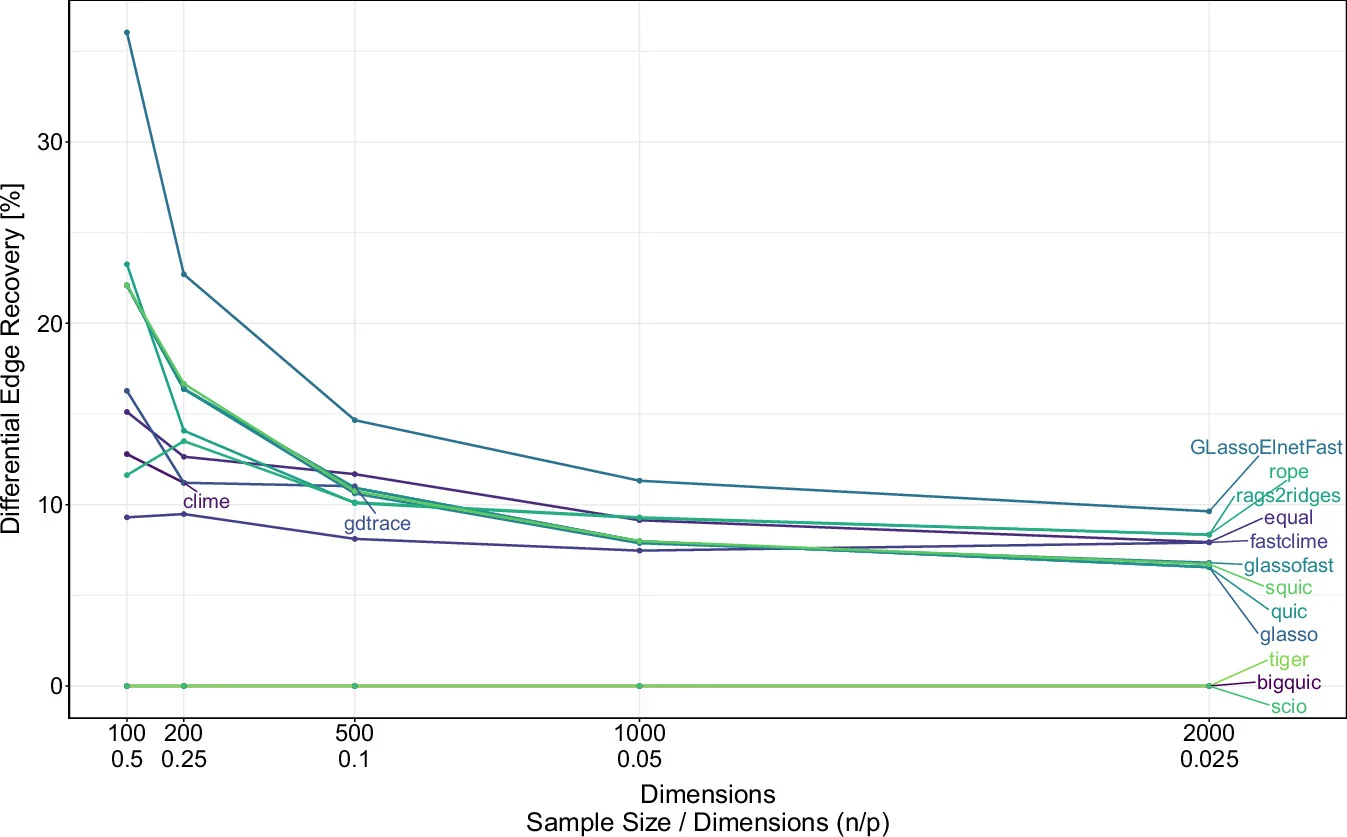
Line plot illustrating the impact of increasing the dimensions on the performances of precision matrix estimation methods, assessed by differential edge recovery rates. The PMEMs *scio*, *tiger*, and *bigquic* did not recover any differential edges across the considered dimensions. Differential edge recovery rates decreased with increasing dimensionality for the remaining PMEMs. However, *GLassoElnetFast* achieved the highest recovery rates across all settings, although the differences between PMEMs became less distinct at higher dimensions.

This section examines how increasing dimensionality affects the performance of different PMEMs. This was analyzed by increasing the number of variables while keeping the sample size constant, effectively decreasing the *n*/*p* ratio. The results are presented in Supplementary Figures 30, 31, and 32. The accuracy of all PMEMs remained largely unaffected by dimensionality. However, the F1 score and normalized MCC decreased with increasing *p* for most methods (Figure S30). *GLassoElnetFast* generally outperformed the other PMEMs across binary classification metrics and was less affected by increasing dimensionality. Matrix norm values and Kullback-Leibler losses increased with higher dimensionality for nearly all PMEMs (Figures S31 and S32). *GLassoElnetFast* mostly achieved the lowest values in matrix norms and KL losses across the tested range of dimensions. Occasionally, lower norm values were observed for *bigquic*, *scio*, and *tiger*. However, these methods again estimated empty precision matrices in all settings. Differential edge recovery rates decreased with increasing dimensionality for most PMEMs. *GLassoElnetFast* again achieved the highest recovery rates across all settings, although differences between methods became less distinct at higher dimensions (Fig. 4).

The sensitivity of differential edge recovery to the weight threshold *τ* was also assessed across dimensions (Figure S33). *Bigquic*, *scio*, and *tiger* did not recover any differential edges at any threshold, and *rags2ridges* and *rope* showed little variation in recovery rates across thresholds. For all other methods, including *GLassoElnetFast*, recovery rates increased at lower threshold values.

### Sample size

**Fig. 5:**
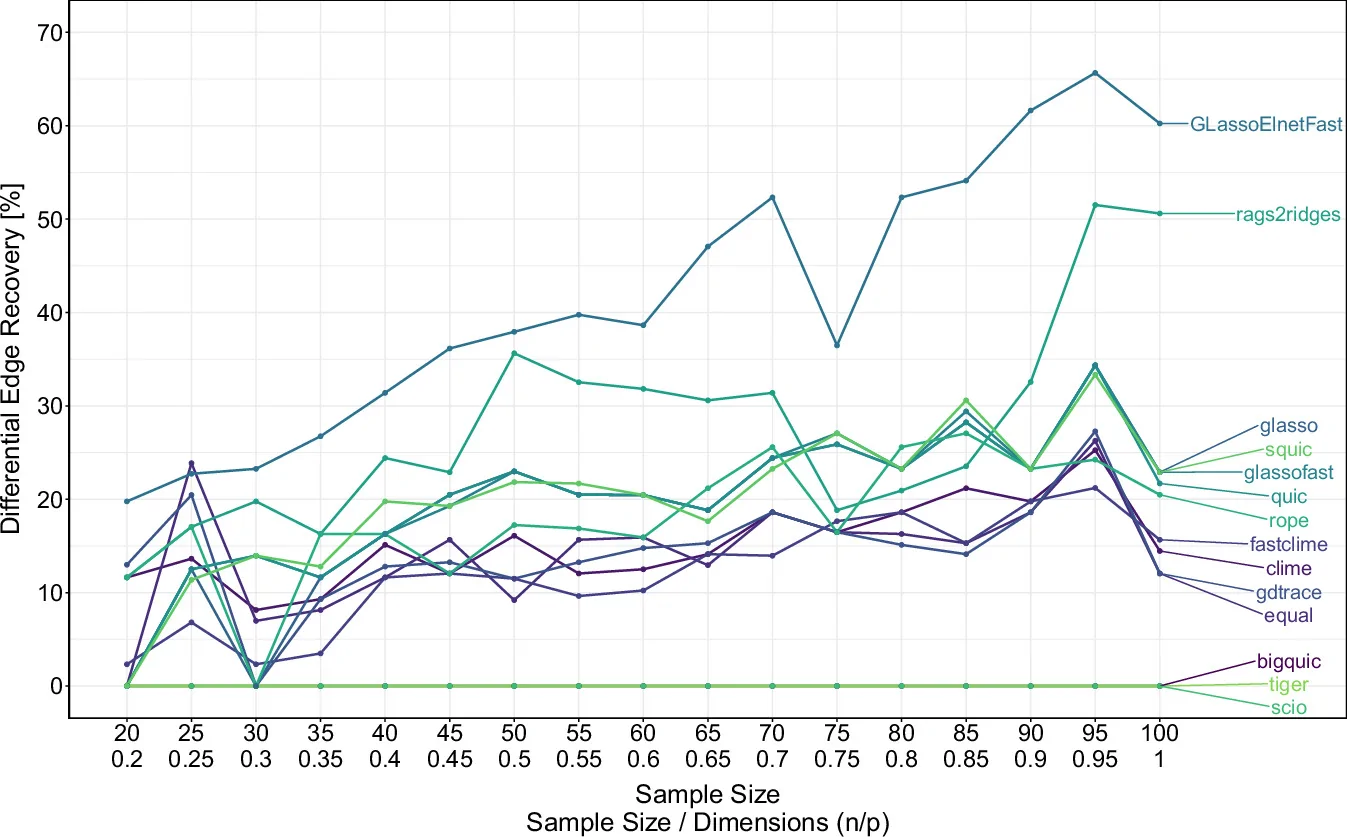
Line plot illustrating the influence of increasing sample size-to-dimension ratios on the performance of precision matrix estimation methods, assessed by differential edge recovery rates. The PMEMs *scio*, *tiger*, and *bigquic* did not recover any differential edges across the considered ratios. All remaining methods exhibited increasing differential edge recovery rates as the sample size-to-dimension ratio was increased. Comparable recovery patterns were observed for *fastclime*, *clime*, *equal*, and *gdtrace*, which showed similar, steadily increasing recovery rates. Slightly higher recovery rates, with otherwise comparable trends, were observed for *glasso*, *squic*, *glassofast*, and *rope*. *Rags2ridges* generally achieved higher recovery rates than these methods across ratios. Among the PMEMs, *GLassoElnetFast* generally achieved higher differential edge recovery than the other approaches.

This section examines how the sample size or *n*/*p* ratio influences the performance of different PMEMs. This was done by increasing the sample size while keeping the dimensionality fixed, thus increasing the *n*/*p* ratio (Figure S40). The results are presented in Fig. 5 and Figures S41-S45. The F1 score and normalized MCC improved for most PMEMs as the sample size increased. *Equal* and *gdtrace* showed the strongest gains, outperforming all other methods in F1 score, accuracy, and normalized MCC as the *n*/*p* ratio increased. For *tiger*, *scio*, *rope*, *rags2ridges*, *bigquic*, and *clime* the F1 score remained constant at 0.18. The accuracy of all methods remained largely unchanged by different *n*/*p* ratios. The normalized MCC values for *tiger*, *scio*, and *bigquic* stayed constant at 0.65, and for *clime*, *rags2ridges*, and *rope* at 0.5. Matrix norm values generally decreased with increasing sample size, though high fluctuations were observed across most PMEMs. In contrast, the norm values increased for *rags2ridges*, reaching a matrix 1-norm of 37.5, Frobenius norm of 103, and spectral norm of 12.5 at *n* = 100. *GLassoElnetFast* initially showed decreasing norm values with larger sample sizes, but these increased again at *n* > 60 (Figures S42, S43). Kullback-Leibler losses followed a similar trend, decreasing for most PMEMs as sample size increased. *GLassoElnetFast* showed an initial decrease until *n* = 85, followed by an increase at higher sample sizes. As before, unlike all other methods, *rags2ridges* exhibited increasing KL losses. These losses reached values as high as 876 for KLL and 150 for reverse KLL at n = 100 (Figures S44 and S45). Differential edge recovery improved with increasing sample size for almost all PMEMs (Fig. 5). Despite their strong performance in binary classification metrics, *equal* and *gdtrace* showed poor results in the differential edge recovery metric, with recovery rates among the lowest at *p* = 100. *GLassoElnetFast* and *rags2ridges* achieved the highest recovery rates. Overall, matrix norm values were not well-aligned with edge recovery performance in this setting. *Rags2ridges*, despite extremely high norm values, and *GLassoElnetFast*, with average norm values, both performed well in recovering the underlying differential structure. The sensitivity of differential edge recovery to the weight threshold *τ* is displayed in Figure S46. *Bigquic* and *scio* did not recover any differential edges at any threshold. *Tiger* showed a similar pattern, except at the lowest thresholds, where non-zero recovery was observed at higher sample sizes. For *clime*, *equal*, *fastclime*, *gdtrace*, *glasso*, *glassofast*, *quic*, and *squic*, recovery rates were higher at lower thresholds. Recovery rates for *GLassoElnetFast*, *rags2ridges*, and *rope* remained comparatively stable across thresholds.

### Sampling distributions

Until this point, all simulated datasets were generated using Gaussian-distributed samples, typically with a zero mean vector. While this reflects the assumptions underlying most estimation methods, RNA-seq data is count-based and, without transformation, is often more appropriately described by a Poisson or negative binomial distribution. Unlike the Poisson distribution, which assumes equal mean and variance, the negative binomial distribution allows for overdispersion, a property commonly observed in real RNA-seq count data and accounted for in widely used differential expression frameworks such as DESeq2 and edgeR. This approach therefore examined how different sampling distributions affect PMEM performance. Datasets were generated from predefined covariance matrices using two Gaussian-based methods (mvrnorm and rmvnorm), a negative binomial-based method (NegBin), and three Poisson-based methods (PoisNor, PoisBinOrdNonNor, and the approach proposed by Yahav and Shmueli [88]). The resulting distributions are shown in Supplementary Figure 47. The performance results are presented in Figures S48-S50. Additionally, Figure S51 shows the runtimes of the different sampling methods. Binary classification metrics were largely unaffected by the move from Gaussian to Poisson-based sampling, with the exception of *GLassoElnetFast*, which showed a slight decrease in F1 score, accuracy, and normalized MCC when applied to Poisson-sampled datasets. Under NegBin-based sampling, however, *equal*, *glasso*, *GLassoElnetFast*, *glassofast*, *quic*, and *squic* all showed a visible performance decrease. Frobenius norm values showed the greatest increase under NegBin-based sampling for *clime*, *equal*, *fastclime*, *glasso*, *glassofast*, *quic*, *rags2ridges*, *scio*, and *squic*. *Gdtrace*, *GLassoElnetFast*, and *tiger* were also affected by NegBin-based sampling in terms of Frobenius norm, though more strongly by Poisson-based sampling. The reverse Kullback-Leibler loss increased substantially under NegBin-based sampling for almost all estimation methods. Overall, Poisson-distributed data noticeably affected PMEM performance, while untransformed negative binomial-distributed data had the biggest impact, particularly on binary classification metrics and Kullback-Leibler losses.

### Runtimes and memory usage

**Fig. 6:**
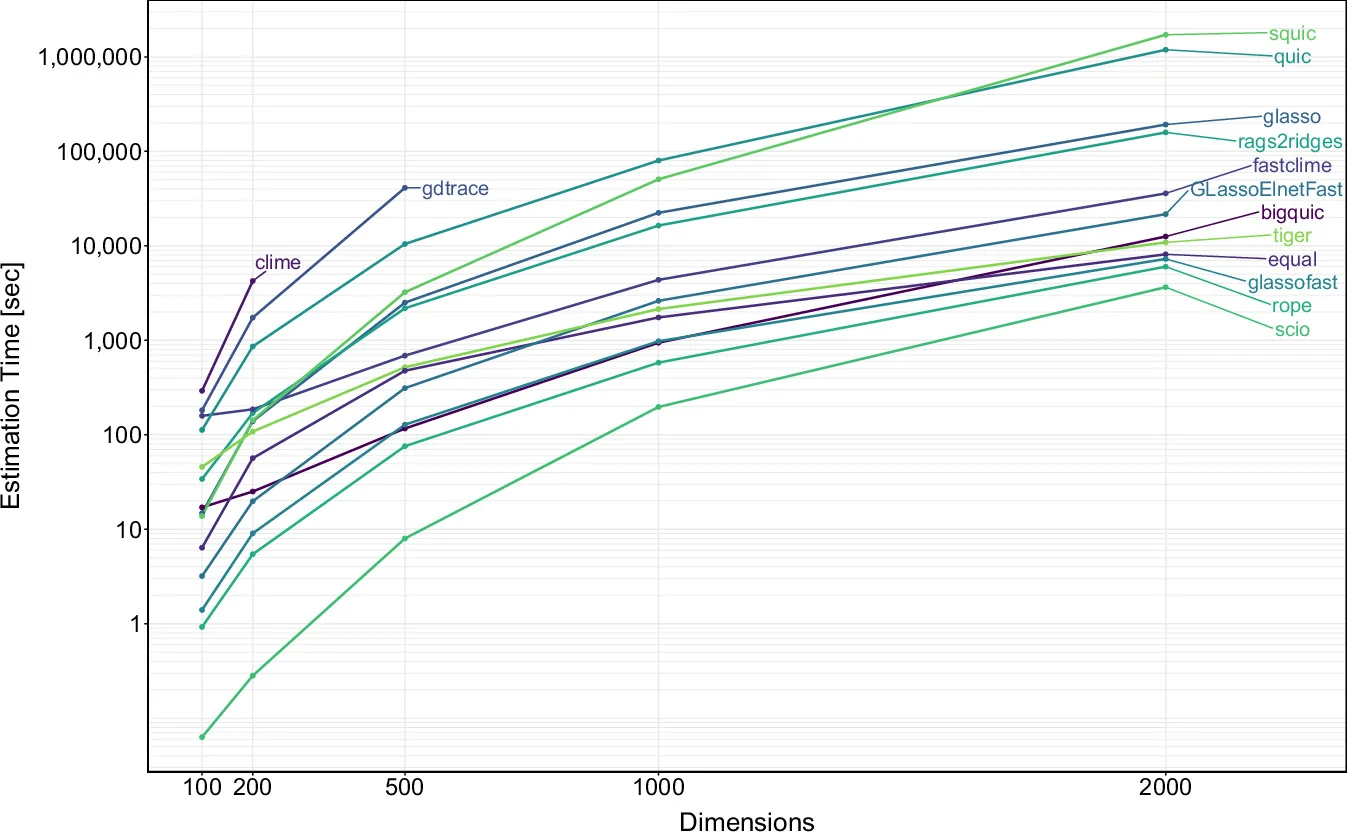
Line plot illustrating the impact of increasing the dimensions on the estimation times of precision matrix estimation methods.

In addition to evaluating PMEM performance across different scenarios, estimation runtimes and peak memory usage were recorded for each method in every approach. A representative subset of these results stems from the dimension increase scenario, in which PMEMs were applied to datasets of increasing dimensionality (*p* = 100, 200, 500, 1000, and 2000). Figure 6 visualizes the corresponding runtimes. It should be noted that *clime* and *gdtrace* were only included up to *p* = 200 and *p* = 500, respectively, due to their substantially longer runtimes compared to the other methods. Overall, runtime progression was consistent across dimensions, with the relative ranking of PMEMs from slowest to fastest remaining largely unchanged. At *p* = 2000, *scio* achieved the shortest runtime, followed by *rope*, *glassofast*, and *equal*. In high-dimensional settings, *glassofast* and *fastclime* showed clearly reduced runtimes compared to their original counterparts, *glasso* and *clime*, respectively, confirming their intended runtime advantages. Similarly, *squic* consistently outperformed *quic* up to *p* = 1000, but at *p* = 2000 it demonstrated the longest runtime among the remaining PMEMs, exceeding 1,710,000 seconds, or roughly 20 days. Finally, estimation times were highly consistent across replicates for all methods, with the largest variation observed in *quic* (Figure S52).

Peak memory usage was similarly consistent across methods and replicates (Figure S53). Standard tracking yielded consistently higher values across methods (1,200-1,300 MB) than isolated tracking (approximately 500 MB), reflecting the additional memory footprint of the continuously running simulation session captured by standard tracking. Within each tracking approach, peak memory usage varied little between methods. Additionally, tracking memory usage had a significant impact on (estimation) runtimes (Figure S54). The estimation runtimes were observed to be longer for both standard and isolated memory tracking than for no memory tracking.

### Real-world datasets

To determine whether the estimator-dependent variability observed in simulation persists in real transcriptomic data, we applied *glassofast*, *GLassoElnetFast*, *equal*, and *rags2ridges* to the COVID-19 (SC2) vs. non-viral ARI (no virus) contrast in GSE156063. For each estimator, we examined the density of the complete estimated partial correlation matrix (*p* = 1000) in both conditions, the structural properties of the differential network derived from the top *k* = 200 differential edges, and the concordance of these differential edges across estimators.

Density of the full 1000 × 1000 partial correlation matrix varies widely across estimators. The ordering is consistent between conditions (control / case): *equal*, 1.6% / 1.4%; *glassofast*, 5.4% / 6.0%; *GLassoElnetFast*, 15.8% / 10.9%. *rags2ridges*, as a ridge-type estimator without explicit sparsification, produces a dense network. This general ordering mirrors the relative sparsity behavior of these four methods observed across the simulation study​.

**Fig. 7:**
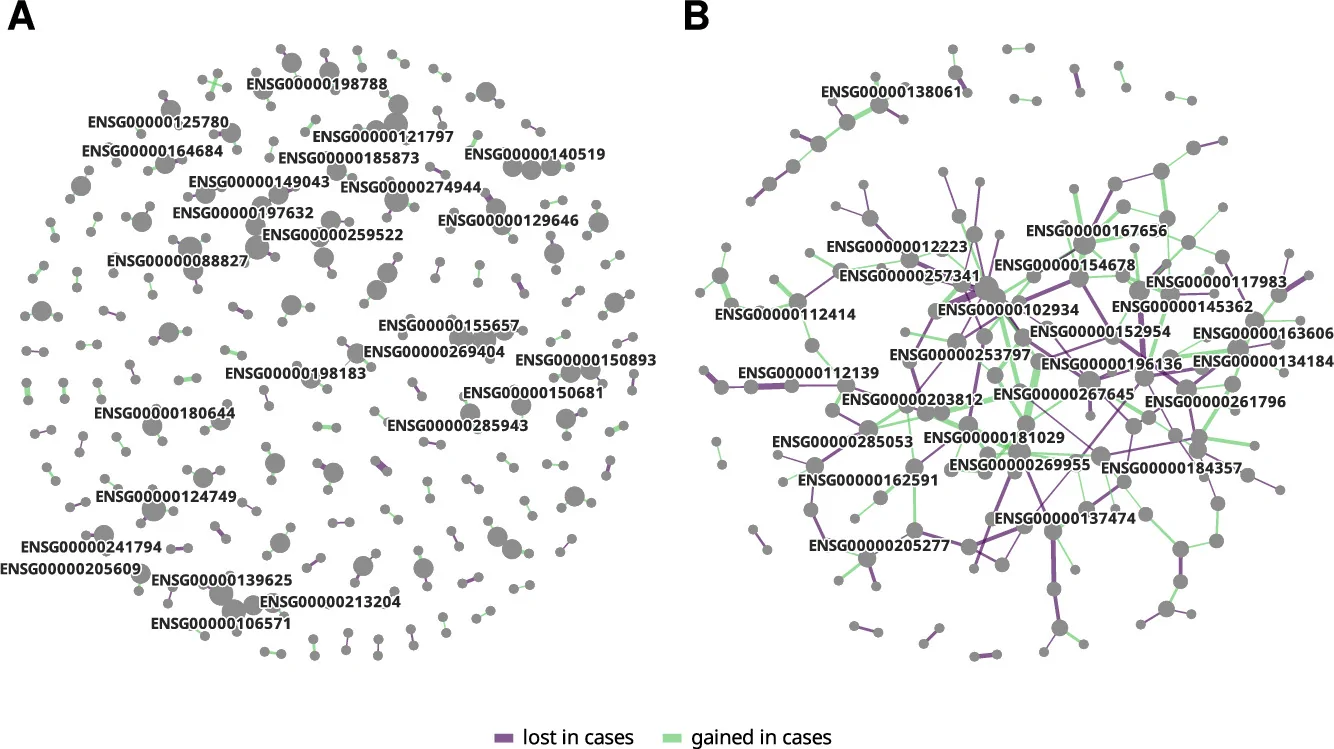
Differential partial correlation networks for the COVID-19 (SC2, *n* = 93, case) vs. non-viral acute respiratory illness (no virus, *n* = 100, control) contrast in GSE156063, estimated using *glassofast* (A) and *GLassoElnetFast* (B) on the top *p* = 1000 genes ranked by pooled within-group variance. Each panel shows the top *k* = 200 gene pairs ranked by absolute difference in partial correlation between conditions.

Restricting to the top *k* = 200 differential edges gives a different, complementary view of the same four estimates. Figure 7 shows the resulting differential networks for *glassofast* (A) and *GLassoElnetFast* (B). Despite being derived from the same expression data, the two networks differ visibly. *glassofast* recovers a markedly less connected network, while *GLassoElnetFast* recovers a denser, more connected network with a small number of genes attaining considerably higher degree than the rest. The corresponding networks for *equal* and *rags2ridges* are shown in Supplementary Figure 55. Table 3 summarizes structural properties of the four differential networks. *GLassoElnetFast* and *rags2ridges* attain the highest mean degree and maximum degree. Hub count is higher for *glassofast* and *equal* than for *GLassoElnetFast* and *rags2ridges*. Transitivity is low across all four methods (≤​ 0.064) and is zero for *glassofast*. Cross-method concordance of the top *k* = 200 differential edges is low overall (Table 4). No pair of estimators shares more than a fifth of its top differential edges, and some pairs share almost none. This indicates that the specific gene pairs identified as being differentially connected depend substantially on the estimator used, consistent with the estimator-dependent variability observed in the simulation study.

**Table 3:** Network properties of the differential partial correlation networks estimated by each PMEM on the COVID-19 vs. non-viral ARI contrast (GSE156063, *p* = 1000).

Method	Mean degree	Max degree	Hub count	Transitivity
glassofast	1.227	3	65	0.000
GLassoElnetFast	2.299	10	31	0.031
equal	1.254	3	68	0.064
rags2ridges	2.260	13	31	0.005

Hub count: number of genes at or above the 90th percentile of degree within the network

**Table 4:** Cross-method concordance of differential edges on GSE156063.

	glassofast	GLassoElnetFast	equal	rags2ridges
glassofast	1.00	0.067	0.187	0.047
GLassoElnetFast	0.067	1.00	0.003	0.034
equal	0.187	0.003	1.00	0.000
rags2ridges	0.047	0.034	0.000	1.00

Pairwise Jaccard index between the top *k* = 200 differential edge sets recovered by each precision matrix estimation method on GSE156063. A value of 1 indicates identical edge sets; 0 indicates no overlap.

## Discussion

In this work, a simulation pipeline was developed that enables the systematic comparison of a large number of precision matrix estimation methods. Both established methods, which often serve as a basis for comparison, and newer methods were analyzed and compared with each other in a variety of simulation approaches. The precision matrix estimation methods were applied under consistent, untuned conditions and were not specifically adjusted to the different simulation settings. Consequently, the results presented here reflect a lower-bound estimate of each method’s performance. Parameter tuning was performed in each scenario, but the setup for this tuning, such as the range of regularization parameters and the cross-validation loss function, was defined once during the development of the simulation pipeline and not modified afterwards. This was mainly done to avoid overfitting the methods to individual scenarios. Furthermore, this also better mimics a case in which the ground truth is not known, such as when using real gene expression data. However, this approach also meant that the best performances of the methods may not have been observed, which might otherwise have been achieved with other settings, such as a different selection of regularization parameters or adapted parameter tuning methods.

Overall, the PMEM performance strongly depended on the simulation approaches, particularly on variations in individual parameters. In addition, the performance of the PMEMs could not be determined solely on the basis of individual evaluation metrics. This large number of influencing factors complicates the evaluation of the PMEMs. Accordingly, simulation strategies with a narrow scope may capture PMEM performance only one-sidedly and can therefore be difficult to generalize. This also has a particular impact on the comparability of results between different publications.

By incorporating various approaches and evaluation metrics, this study enabled a more comprehensive evaluation of the analyzed PMEMs. Across the majority of settings, most PMEMs achieved only moderate to poor performance in recovering the differential network structure, while a smaller number of methods performed comparatively better, as detailed below. It was observed that the densities of the estimated matrices did not change over the different densities of the ground truth matrices. The exception was *GLassoElnetFast*, which always slightly overestimated the density, but more importantly resulted in an adjusted density of $\widehat{\Theta }$ as the density of Θ increased. This points to inherent weaknesses of both *ℓ*_1_- and *ℓ*_2_-regularized PMEMs. The *ℓ*_1_ regularization introduces a structural bias towards sparse solutions. This is often preferred, since zero partial correlations directly impose interpretable graph structures [45]. However, this potentially introduces an issue, if, for example, the networks are estimated too sparsely, clusters or hub nodes in the estimated network may remain undetected. These hub nodes, in particular, could hold significant biological relevance [64]. This problem is only amplified when the precision matrices or partial correlation networks of two conditions are estimated too sparsely and the differential edges are to be identified. This was observed, for example, with the PMEMs *equal*, *fastclime*, and *gdtrace*, which usually estimated the matrix too sparsely and also exhibited below-average differential edge recovery. Even if other regularization parameters can lead to denser estimates, the respective parameter tuning method only selects the parameters that, for example, reduce the likelihood loss, regardless of the density of the matrices. As for the *ℓ*_2_-regularized PMEMs, or overall the PMEMs that estimate dense matrices, *rags2ridges* demonstrated the poorest performance in terms of the matrix norms of the unfiltered estimated matrices. Furthermore, the F1 score and accuracy fail to evaluate the real performance if $\widehat{\Theta }$ is fully dense. The MCC identifies these as random.

Nevertheless, *rags2ridges* outperformed all other PMEMs, except *GLassoElnetFast*, in most simulation approaches in the differential edge recovery metric. This means that neither the matrix norms nor the binary classification metrics values provide any direct indication of the PMEMs’ performance in differential network analysis. It also became evident that estimating a dense matrix alone is not sufficient if the weights of the individual edges are not accurately recovered. Methods such as *rope* and *clime* generally performed similarly to the sparse estimators in terms of differential edge recovery. In contrast, *rags2ridges* and, most notably, *GLassoElnetFast* stood out with generally better edge recovery.

For the DER-based comparisons presented in the main text, a comparatively high threshold *τ* was used to filter the ground-truth differential edges. As DER only requires an edge to be present in both the thresholded ground-truth set and the top-*k* set of the estimated network, without the corresponding edge weights needing to agree in magnitude or rank, a low value of *τ* makes this criterion easier to satisfy. A smaller *τ* retains a larger number of ground-truth edges, which increases *k* and thus the size of both edge sets being compared. As both sets grow, the likelihood of incidental overlap increases as well, inflating DER independently of whether the estimated edge weights truly reflect the ground-truth differential structure. A high *τ*, in contrast, restricts the ground-truth set to only the most important differential edges and correspondingly reduces *k*, yielding a stricter and more informative evaluation of whether a PMEM’s largest estimated differential effects coincide with the true strongest ones. A method for which DER is high under a low-threshold but lower under a high-threshold recovers many differential edges when the criterion is loose, but fails to prioritize the true strongest differential edges among its own largest estimated weights. This indicates that its estimated edge magnitudes are not well aligned with the true differential structure. This motivated the use of a comparatively high threshold for the presented comparisons, as it provides a more discriminating evaluation of whether the PMEMs correctly recover the most important differential edges, rather than crediting the recovery of large numbers of weaker, less distinguishable edges. Additionally, across the threshold sensitivity analyses, the DER values for most thresholds were found to cluster closely together. Clear separation was mainly observed at the lowest and highest tested thresholds. This indicates that the exact value of *τ* has limited influence on the evaluation for most PMEMs. The deciding factor is whether a low, intermediate or high threshold is chosen.

The simulated analyses presented here demonstrate that the performance of the PMEMs is substantially impacted by most of the analyzed parameters. Notably, covariance structure, matrix density and sample size had a substantial impact on estimator performance. This shows that less extensive evaluations risk testing and evaluating PMEMs too selectively, which can limit the comparability of findings across studies. Given the influence of numerous factors, including covariance structure, density, covariance values, sample size-to-dimension ratio, sampling distribution, normalization, and the choice of regularization parameters, reproducing findings across evaluation studies in this field may be challenging. A comprehensive evaluation that systematically varies all relevant parameters and incorporates diverse evaluation metrics is essential for a robust comparison of precision matrix estimation methods. Overall, many weaknesses of the various PMEMs were identified, such as excessive regularization, scaling problems and low consistency. Notably, some PMEMs are only poorly documented, further limiting their usefulness. In the differential network analysis, *GLassoElnetFast* was identified as the most accurate method for recovering the simulated differences between conditions, among the scenarios evaluated in this study. Additionally, *GLassoElnetFast* benefited more from optimal conditions, such as a high signal-to-noise ratio and a high sample-to-dimension ratio.

As a separate observation, the Poisson method proposed by Yahav and Shmueli [88] is recommended for future simulations, as it produces results comparable to the other Poisson-based approaches while being significantly faster. The method is described in the Supplementary Information (Section 11), and runtime comparisons are provided in Figure S51.

Applying the four PMEMs *glassofast*, *GLassoElnetFast*, *equal*, and *rags2ridges* to the same real dataset shows a comparable pattern to the simulation study. Estimator choice affects the recovered (differential) network, and overlap among the differential edges recovered by different estimators remains small. The specific genes flagged as important therefore appear to depend, at least in part, on which PMEM is used. This estimator-dependent variability observed on real transcriptomic data mirrors the inconsistencies identified across the simulation study and indicates that network inference for differential co-expression analysis remains challenging in practice. Different PMEMs are likely to recover overlapping but non-identical sets of biological signal, and estimator choice should not be treated as a minor implementation detail.

Additionally, this has a practical implication beyond benchmarking. An estimator that is too sparse risks under-recovering true structure, so estimator choice should be guided by expected network density and by an estimator’s known performance in the relevant (*n*, *p*) setting, rather than convention or convenience.

## Conclusions

Precision matrix estimation is commonly applied to gene expression datasets to infer underlying network structures [64; 101–104].

We show that different estimators vary substantially both in their overall performance and in their sensitivity to data properties. Furthermore, comparable variability was observed in our application to real transcriptomic data. Consequently, the choice of estimator can strongly influence the resulting networks, with the risk of missing biologically relevant interactions or recovering spurious ones. This reduces confidence in the stability and interpretability of real-world analyses and highlights the importance of careful method selection and benchmarking.

Within this context, the comparative analyses provide several methodological insights. Dense estimators apply global shrinkage without creating exact zeros in the precision matrix. Consequently, they generate results that are challenging to interpret and typically necessitate post-hoc edge selection to derive a meaningful network structure. Very sparse estimators, which enforce hard sparsity through zero-valued entries, can be useful for recovering single networks directly. However, their utility in differential network analysis is limited, as strong regularization risks eliminating too many true edges. Elastic net-based approaches combine the advantages of sparse and dense estimators by applying a more flexible form of regularization. This makes the inferred networks directly interpretable and suitable for differential analysis, since excessive sparsity is avoided and the risk of eliminating important edges is reduced. These results suggest that further methodological development of elastic net-based precision matrix estimation could be especially valuable. Beyond differential gene co-expression analysis, the results of this study also provide insights into how the estimation of individual precision matrices depends on factors such as covariance structure, density, and sample size. These findings may therefore be of relevance in other domains where precision matrix estimation is applied. These include single-cell transcriptomics, proteomics, and metabolomics. Beyond biology, similar challenges arise in fields like neuroscience and finance, where reliable precision matrix estimation is equally essential for uncovering underlying dependency structures.

Taken together, and within the scope of the analyses and evaluation criteria we considered, these results provide a clear practical recommendation. When sparse solutions are required, *glassofast* proves to be a reliable option. Across the settings evaluated, and especially in differential network analysis, *GLassoElnetFast* represents a robust choice. *Rags2ridges* may also be considered when post-hoc thresholding is acceptable.

### List of abbreviations and notation

The abbreviations used in this manuscript are summarized in Table 5, and the mathematical notation is given in Table 6.

**Table 5:** Overview over the abbreviations

Abbreviation	Description
ARI	Acute Respiratory Illness
CDF	Cumulative Distribution Function
chNet	hierarCHical differential NETwork analysis model
DE	Differentially Expressed
DER	Differential Edge Recovery
DGE	Differential Gene Expression
GEO	Gene Expression Omnibus
GGM	Gaussian Graphical Model
HDLSS	High-Dimensional Low Sample Size
ICF	Iterative Conditional Fitting
KLL	Kullback-Leibler Loss
Lasso	Least Absolute Shrinkage and Selection Operator
MCC	Matthews Correlation Coefficient
PGM	Probabilistic Graphical Model
PMEM	Precision Matrix Estimation Method
RKLL	Reverse Kullback-Leibler Loss
RNA-seq	RNA Sequencing
ROC	Receiver Operating Characteristic
RSS	Resident Set Size
SC2	SARS-CoV-2
WGCNA	Weighted Gene Co-expression Network Analysis

**Table 6:** Overview of notation

Symbol	Description
*X*	*p*-dimensional random vector
**X**	Data matrix in ${{\mathbb{R}}}^{n\times p}$
*μ*, *μ*_*j*_	Population mean (vector / component)
${\bar{x}}_{j}$	Sample mean of variable *j*
$\bar{X}$	Sample mean vector
${\sigma }_{j}^{2},{s}_{j}^{2}$	Variance (population, sample)
*σ*_*ij*_	Covariance between *X*_*i*_ and *X*_*j*_
Cov(*X*_*i*_, *X*_*j*_), *s*_*ij*_	Covariance (population, sample)
Σ	Covariance matrix
S	Sample covariance matrix
R	Correlation matrix
*ρ*_*ij*_	Correlation between *X*_*i*_ and *X*_*j*_
*ρ*_*ij*⋅−*ij*_	Partial correlation between *X*_*i*_ and *X*_*j*_
Ψ	Partial correlation matrix
${\Psi }_{\Delta },{\widehat{\Psi }}_{\Delta }$	True / estimated differential partial correlation matrix
Θ ≡ Σ^−1^	Precision (concentration) matrix
*D*_*Θ*_	Diagonal matrix of Θ
$\Delta \equiv \widehat{\Theta }-\Theta$	Difference matrix (estimated minus true precision matrix)
**A**, **B**	General matrices
$\widehat{{\bf{A}}}$	Estimate of matrix **A**
**I**	Identity matrix
∥**A**∥	Norm of matrix **A**
*ϕ*	Eigenvalue
*p*	Number of variables
*n*, *N*	Sample size and population size
*k*	Number of top-ranked / important edges (differential edge recovery)
*K*	Number of cross-validation folds or mixture components
*G*	Graph
*V*	Set of vertices (nodes)
*E*	Set of edges
∣*V*∣	Number of vertices
∣*E*∣	Number of edges
$\tau ,{\tau }_{\max }$	Edge-weight threshold and its maximum value (differential edge recovery)
*m*, *γ*	Number of threshold grid points and power-law spacing exponent (differential edge recovery)
*λ*, *ρ*, *α*	Regularization parameters
${\mathbb{E}}$	Expectation operator
Var( ⋅ )	Variance operator
*ℓ*_1_, *ℓ*_2_	*ℓ*_1_ and *ℓ*_2_ norms / penalties

## Supplementary information

Supplementary Material 1

## Data Availability

The datasets supporting the conclusions of this article are available in the PMEM Evaluator github repository: ​https://github.com/scibiome/PMEM_Evaluator.git​​.

## Code availability

All simulations and analyses were performed on a server equipped with an AMD EPYC 745232-core/64-thread processor and 500 GB of RAM. Project name: PMEM-Evaluator. Dockerhub: https://hub.docker.com/r/matthias587/pmem-evaluator. Operating system: platform independent (docker). Programming language: R (4.6.1). Source and configuration files: ​https://github.com/scibiome/PMEM_Evaluator.git. License: GPL-3.0 license.
