# Supplementary material for: Benchmarking precision matrix estimation methods for differential co-expression network analysis
Source: BMC Bioinformatics. 2026 Nov 11;27:275. doi: 10.1186/s12859-026-06731-6 (PMC13761054; doi:10.1186/s12859-026-06731-6)
Supplement: Supplementary file 1 [file 12859_2026_6731_MOESM1_ESM.pdf]

Benchmarking precision matrix estimation methods for differential co-expression network analysis
Supplementary Information

Matthias Overmann[1, 2], Gordon Grabert[1, 2, †], and Tim Kacprowski[1, 2, †, *]

[1]Institute of Data Science in Biomedicine, Technische Universität Braunschweig
[2]Braunschweig Integrated Centre of Systems Biology (BRICS), Technische Universität Braunschweig

[*]Corresponding author, Institute of Data Science in Biomedicine, Rebenring 56, Braunschweig, Lower Saxony 38106, Germany. E-mail: t.kacprowski@tu-braunschweig.de
[†]Joint senior authorship.

Contents

1 Supplementary Information for Methods **5**

1.1 Covariance 1 Methods . . . 5

1.1.1 Scale Free 2 Construction . . . 7

1.2 Covariance 2 Methods . . . 10

1.3 Sampling . . . 11

1.4 Evaluation . . . 12

1.4.1 Binary Classification Metrics . . . 12

1.4.2 ROC Curve Examples . . . 12

2 Supplementary Information for Replicates **15**

3 Supplementary Figures for Covariance 1 Methods **17**

4 Supplementary Figures for Covariance Density **20**

5 Supplementary Information for Covariance 2 Methods **24**

6 Supplementary Figures for Covariance Increase **29**

7 Supplementary Figures for Dimensions **33**

8 Supplementary Information for Means **37**

9 Supplementary Information for Normalization **41**

10 Supplementary Figures for Sample Size **45**

11 Supplementary Information for Sampling **52**

12 Supplementary Figures for Runtimes **59**

13 Supplementary Figures for Real-World Analysis **62**

List of Figures

1 Covariance 1 methods: single block, multiple block, and band network . . . 5
2 Covariance 1 method: network of scale free 1 . . . 6
3 Covariance 1 method: scale free 1 . . . 6
4 Covariance 1 method: network of scale free 2 . . . 7
5 Covariance 1 method: scale free 2 . . . 8
6 Covariance 1 method: single block icf . . . 8
7 Covariance 1 method: multiple block icf . . . 9
8 Covariance 1 method: random graph icf . . . 9
9 Covariance 1 method: random hubs icf . . . 9
10 Covariance 2 methods. A: knockout, B: mutate. . . . 10
11 Sample covariance matrix: mvrnorm . . . 11
12 Example ROC curve for *GLassoElnetFast* . . . 13
13 Example ROC curve for *rags2ridges* . . . 14
14 Reproducibility of PMEMs . . . 16
15 Impact of different Covariance 1 Methods on Binary Classification Metrics . . . 17
16 Impact of different Covariance 1 Methods on Matrix Norms . . . 18
17 Impact of different Covariance 1 Methods on Kullback-Leibler Losses . . . 19
18 Matrices with different densities . . . 20
19 Impact of different densities of Θ_1 on Binary Classification Metrics . . . 21
20 Impact of different densities of Θ_1 on Matrix Norms . . . 22
21 Impact of different densities of Θ_1 on Kullback-Leibler Losses . . . 23
22 Impact of different Covariance 2 Methods on Binary Classification Metrics . . . 25
23 Impact of different Covariance 2 Methods on Matrix Norms . . . 26
24 Impact of different Covariance 2 Methods on Kullback-Leibler Losses . . . 27
25 Impact of different Covariance 2 Methods on differential Edge Recovery . . . 28
26 High and Low Signal-to-Noise Ratios . . . 29
27 Impact of different Covariance Values in Σ_1 on Binary Classification Metrics . . . 30
28 Impact of different Covariance Values in Σ_1 on Matrix Norms . . . 31
29 Threshold Sensitivity of Differential Edge Recovery Across different Covariance Values in Σ_1 . . . 32
30 Impact of increasing Dimensions on Binary Classification Metrics . . . 33
31 Impact of increasing Dimensions on Matrix Norms . . . 34
32 Impact of increasing Dimensions on Kullback-Leibler Losses . . . 35
33 Threshold Sensitivity of Differential Edge Recovery Across increasing Dimensions . . . 36
34 Impact of shifting Means on Binary Classification Metrics . . . 38
35 Impact of shifting Means on Matrix Norms . . . 39
36 Impact of shifting Means on Kullback-Leibler Losses . . . 40
37 Impact of different Normalization Strategies on Binary Classification Metrics . . . 42
38 Impact of different Normalization Strategies on Matrix Norms . . . 43
39 Impact of different Normalization Strategies on Kullback-Leibler Losses . . . 44
40 Matrices with different underlying dimensions to sample size ratios . . . 45
41 Impact of increasing Sample Sizes on Binary Classification Metrics . . . 46
42 Impact of increasing Sample Sizes on Matrix Norms . . . 47
43 Impact of increasing Sample Sizes on Matrix Norms, including *rags2ridges* . . . 48
44 Impact of increasing Sample Sizes on Kullback-Leibler Losses . . . 49
45 Impact of increasing Sample Sizes on KL Losses, including *rags2ridges* . . . 50
46 Threshold Sensitivity of Differential Edge Recovery Across increasing Sample Sizes . . . 51

47 Distributions of different Sampling Methods 52
48 Impact of different Sampling Methods on Binary Classification Metrics 53
49 Impact of different Sampling Methods on Matrix Norms 54
50 Impact of different Sampling Methods on Kullback-Leibler Losses 55
51 Runtimes of different Sampling Methods 58
52 Runtimes of PMEMs over Replicates 59
53 Peak Memory of PMEMs over Replicates 60
54 Estimation Runtimes across Memory Tracking Modes 61
55 Differential Partial Correlation Networks Estimated by equal and rags2ridges on GSE156063 62

1 Supplementary Information for Methods

1.1 Covariance 1 Methods

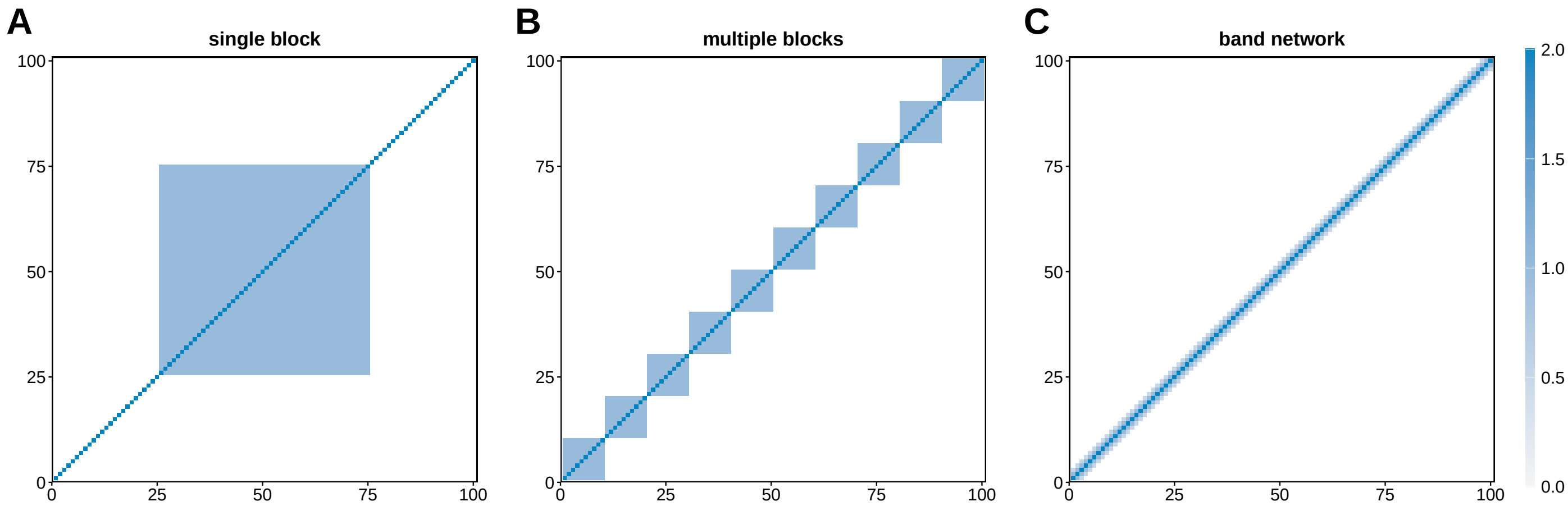

Supplementary Figure 1: Example covariance matrices generated with: single block, multiple block and band network. A: single block structure (size = 3, position = center), B: multiple blocks (cliques = 10), C: band network. All diagonal values are set to 2 and off-diagonal values in single block and multiple blocks are set to 1.

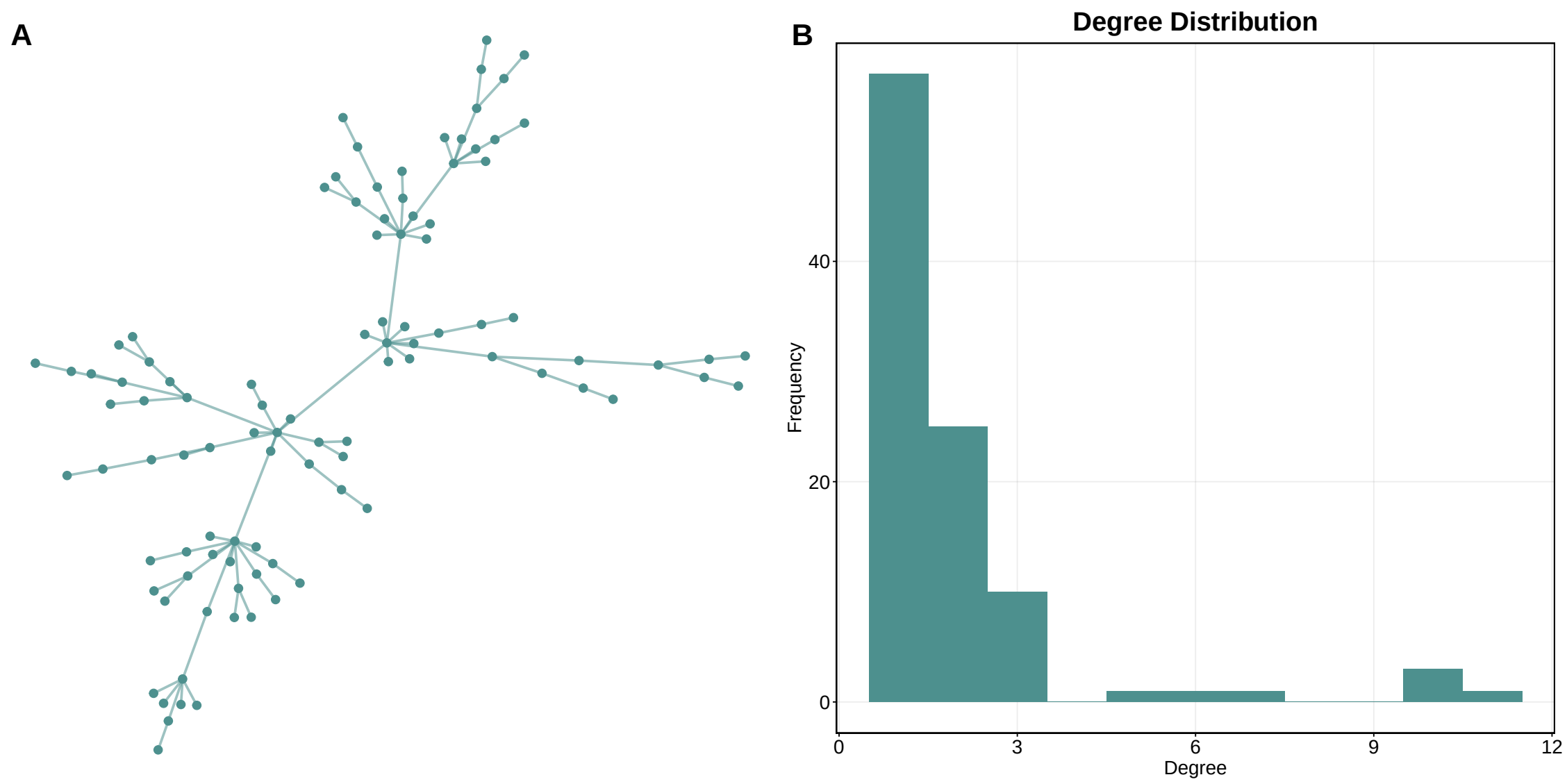

Supplementary Figure 2: Example network and degree distribution of the initial network constructed by scale free 1.

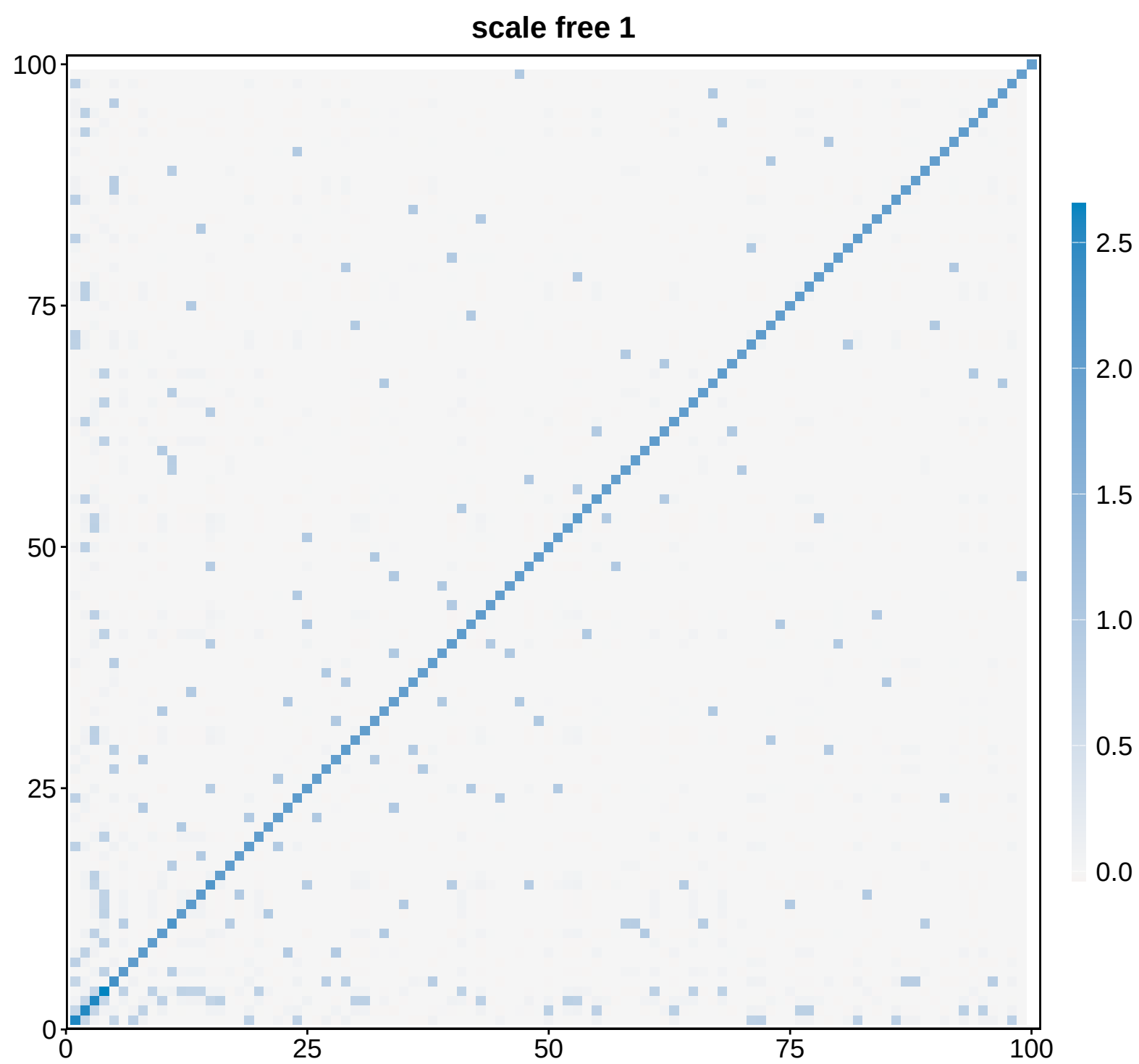

Supplementary Figure 3: Example covariance matrix generated with the scale free 1 method.

1.1.1 Scale Free 2 Construction

To simulate Θ_1, for $j \neq k$:

$$(\Theta_1)_{jk} = \begin{cases} 0, & (j,k) \notin E_1, \\ 0.5, & (j,k) \in E_1 \text{ with probability } 0.5, \\ -0.5, & (j,k) \in E_1 \text{ with probability } 0.5. \end{cases}$$

To simulate Θ_2, for $j \neq k$:

$$(\Theta_2)_{jk} = \begin{cases} (\Theta_1)_{jk}, & (j,k) \in E_1 \cap E_2, \\ 0, & (j,k) \notin E_2, \\ 0.5, & (j,k) \in E_2 \setminus E_1 \text{ with probability } 0.5, \\ -0.5, & (j,k) \in E_2 \setminus E_1 \text{ with probability } 0.5. \end{cases}$$

For $m \in \{1, 2\}$, the diagonal entries are defined as

$$(\Theta_m)_{jj} = \sum_{k \neq j} |(\Theta_m)_{jk}| + u_m, \quad j = 1, \ldots, p,$$

where u_m is chosen such that the smallest eigenvalue satisfies $\phi_{\min}(\Theta_m) = 0.1$. Afterwards, Θ_m is normalized such that the corresponding covariance matrix $\Sigma_m = \Theta_m^{-1}$ satisfies $(\Sigma_m)_{jj} = 1$, $j = 1, \ldots, p$.

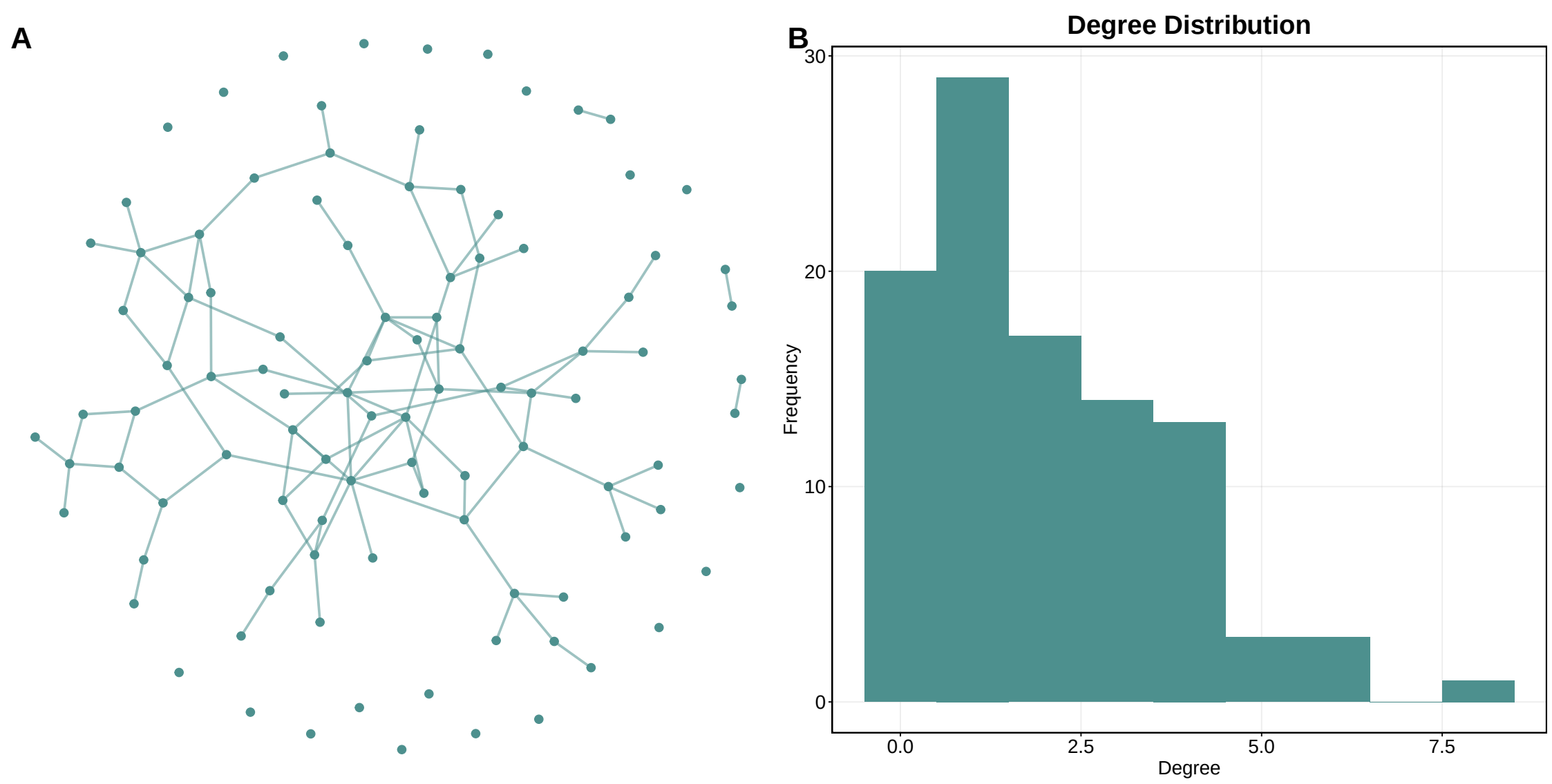

Supplementary Figure 4: Example network and degree distribution of the initial network constructed by scale free 2.

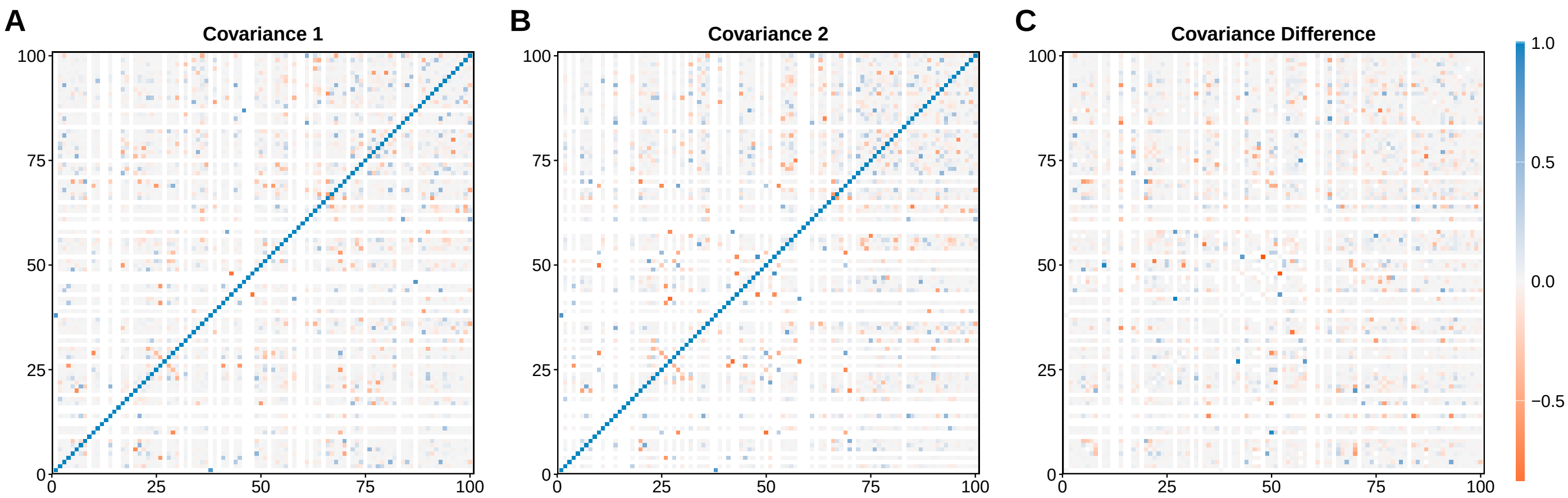

Supplementary Figure 5: Example covariance matrices ($\Sigma_1, \Sigma_2,$ and Σ_Δ) generated with the scale free 2 method.

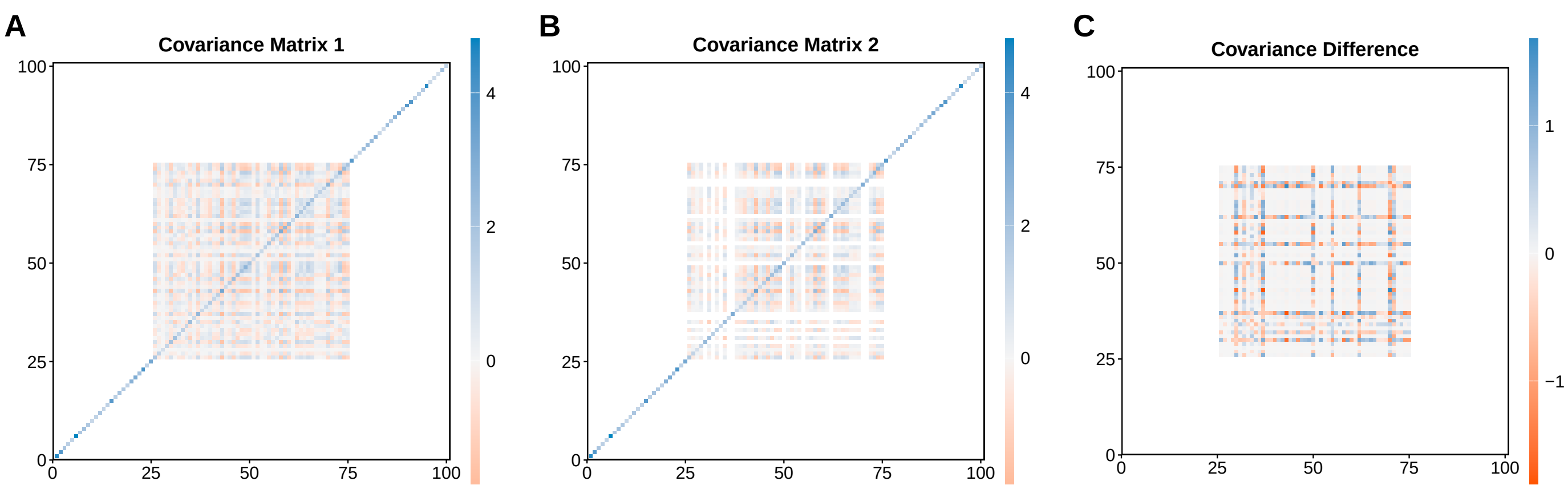

Supplementary Figure 6: Example covariance matrices generated with covariance 1 method single block icf. A: Covariance matrix for condition 1 Σ_1, B: Covariance matrix for condition 2 Σ_2, C: Differential covariance matrix Σ_Δ.

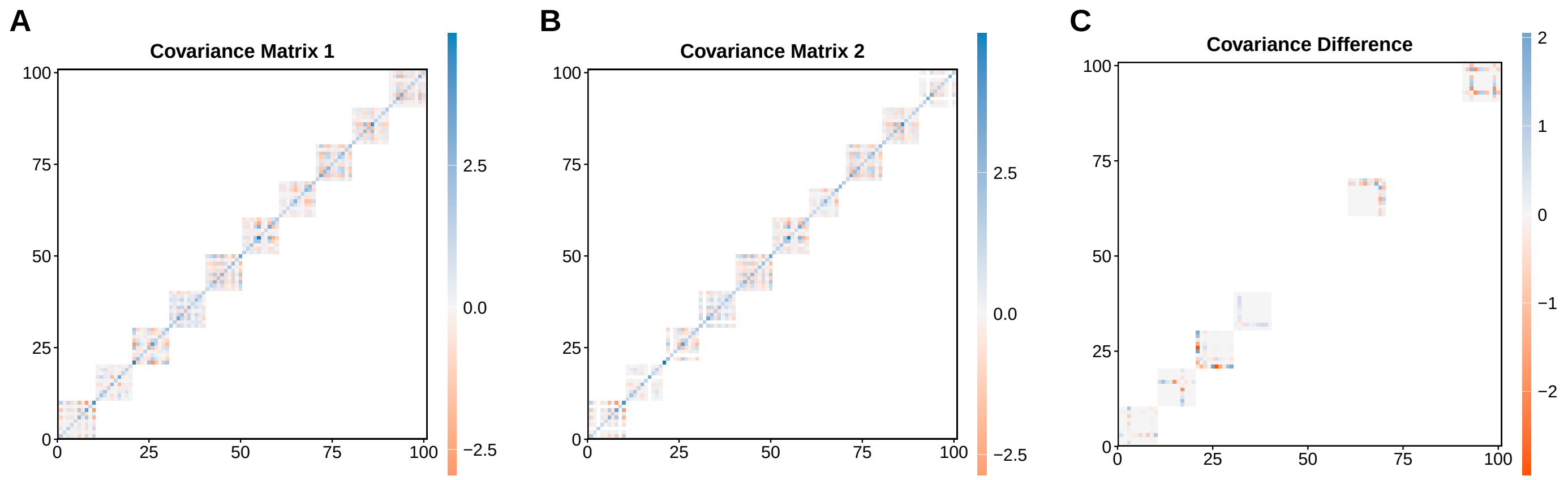

Supplementary Figure 7: Example covariance matrices generated with covariance 1 method multiple block icf. A: Covariance matrix for condition 1 Σ_1, B: Covariance matrix for condition 2 Σ_2, C: Differential covariance matrix Σ_Δ.

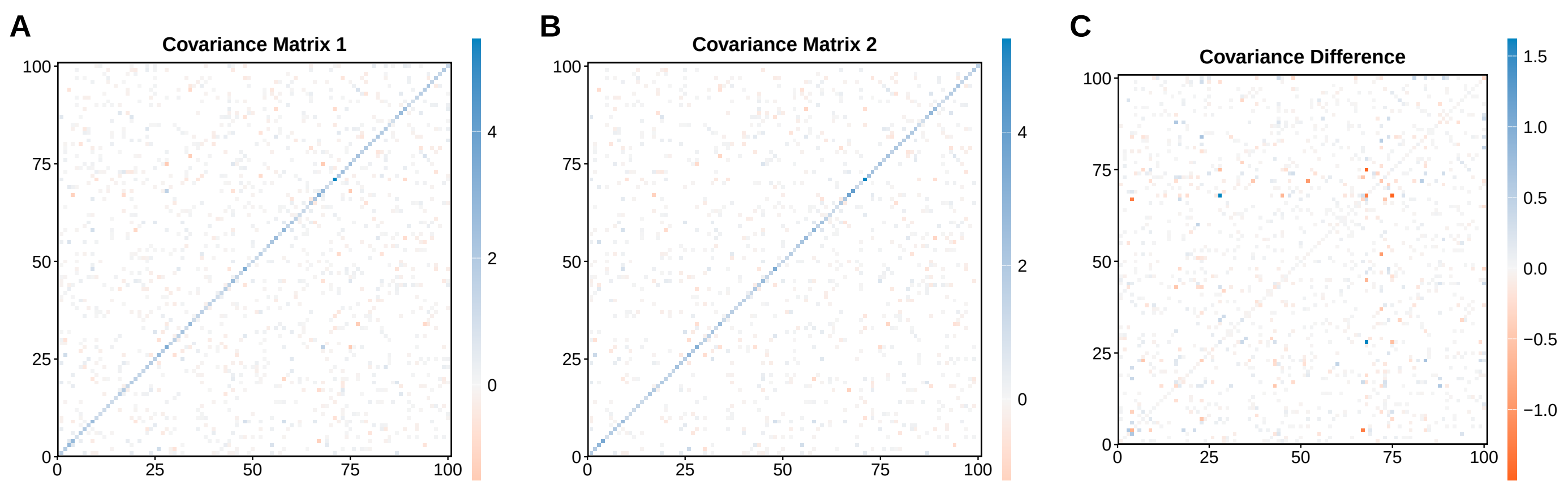

Supplementary Figure 8: Example covariance matrices generated with covariance 1 method random graph icf. A: Covariance matrix for condition 1 Σ_1, B: Covariance matrix for condition 2 Σ_2, C: Differential covariance matrix Σ_Δ.

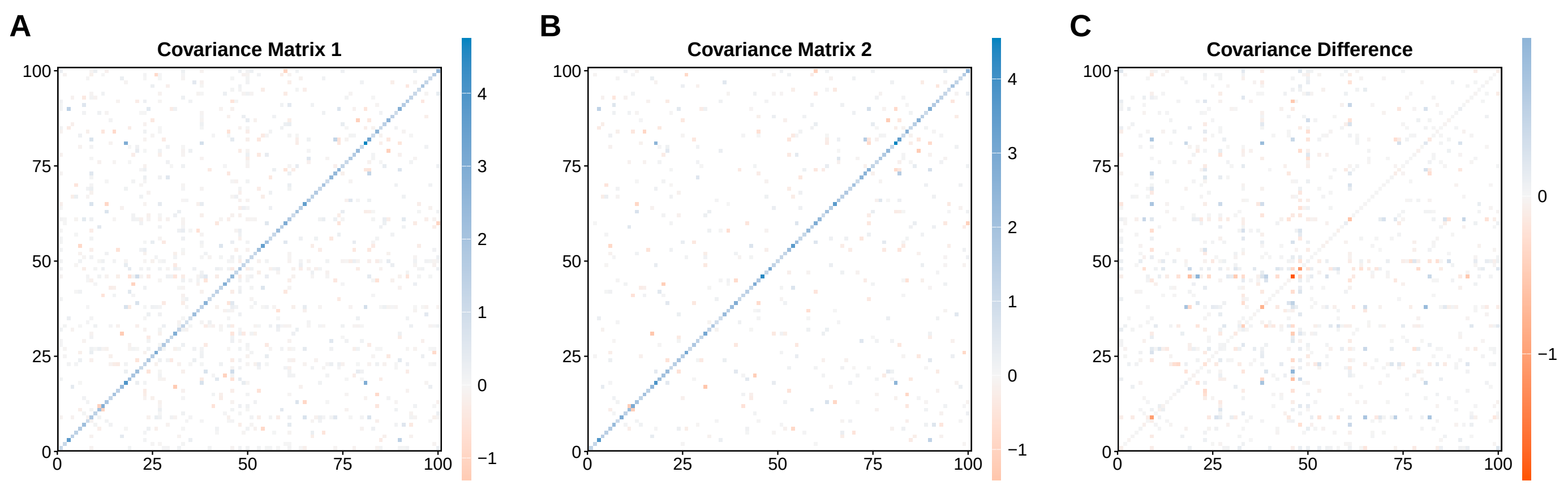

Supplementary Figure 9: Example covariance matrices generated with covariance 1 method random hubs icf. A: Covariance matrix for condition 1 Σ_1, B: Covariance matrix for condition 2 Σ_2, C: Differential covariance matrix Σ_Δ.

1.2 Covariance 2 Methods

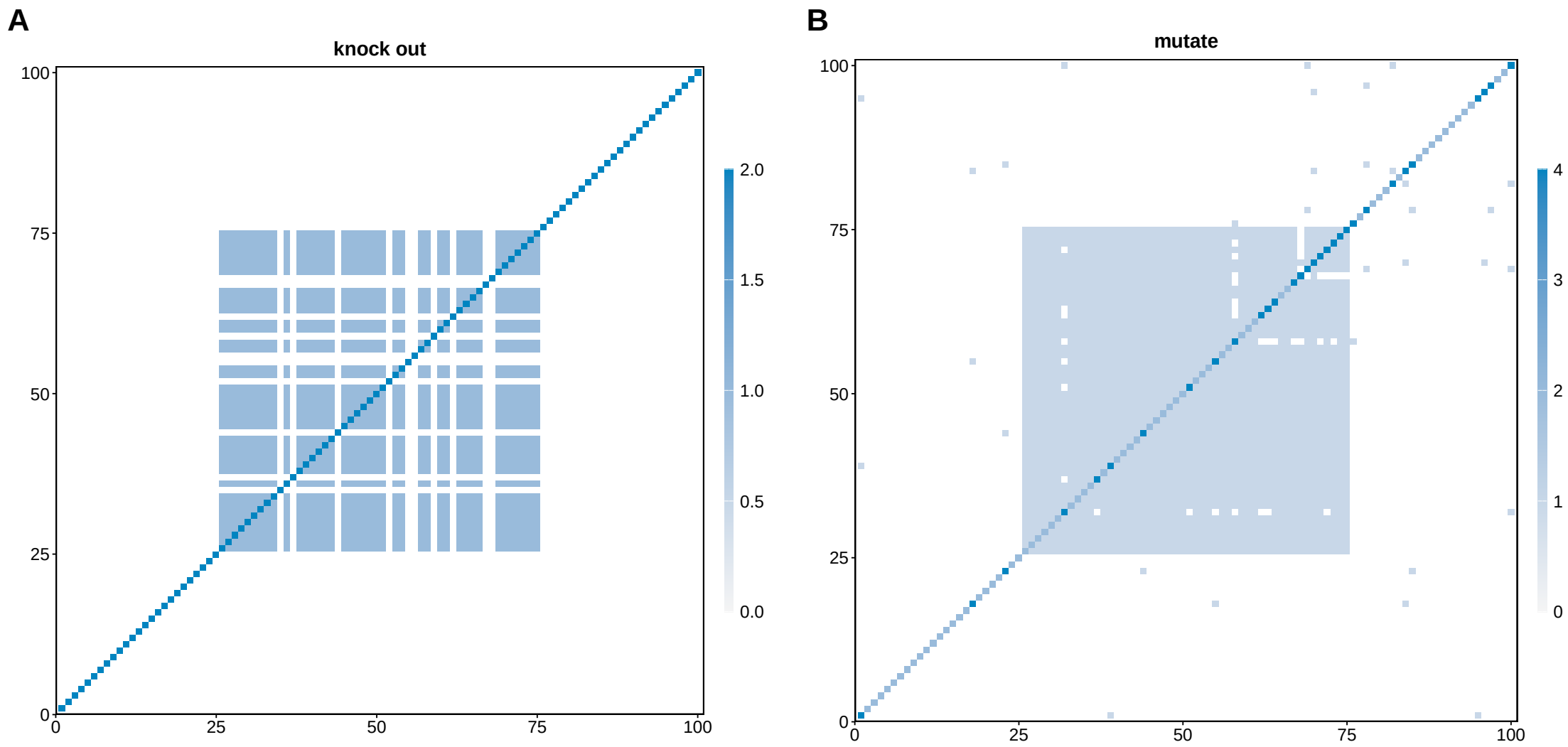

Supplementary Figure 10: Covariance 2 methods. A: knockout, B: mutate.

1.3 Sampling

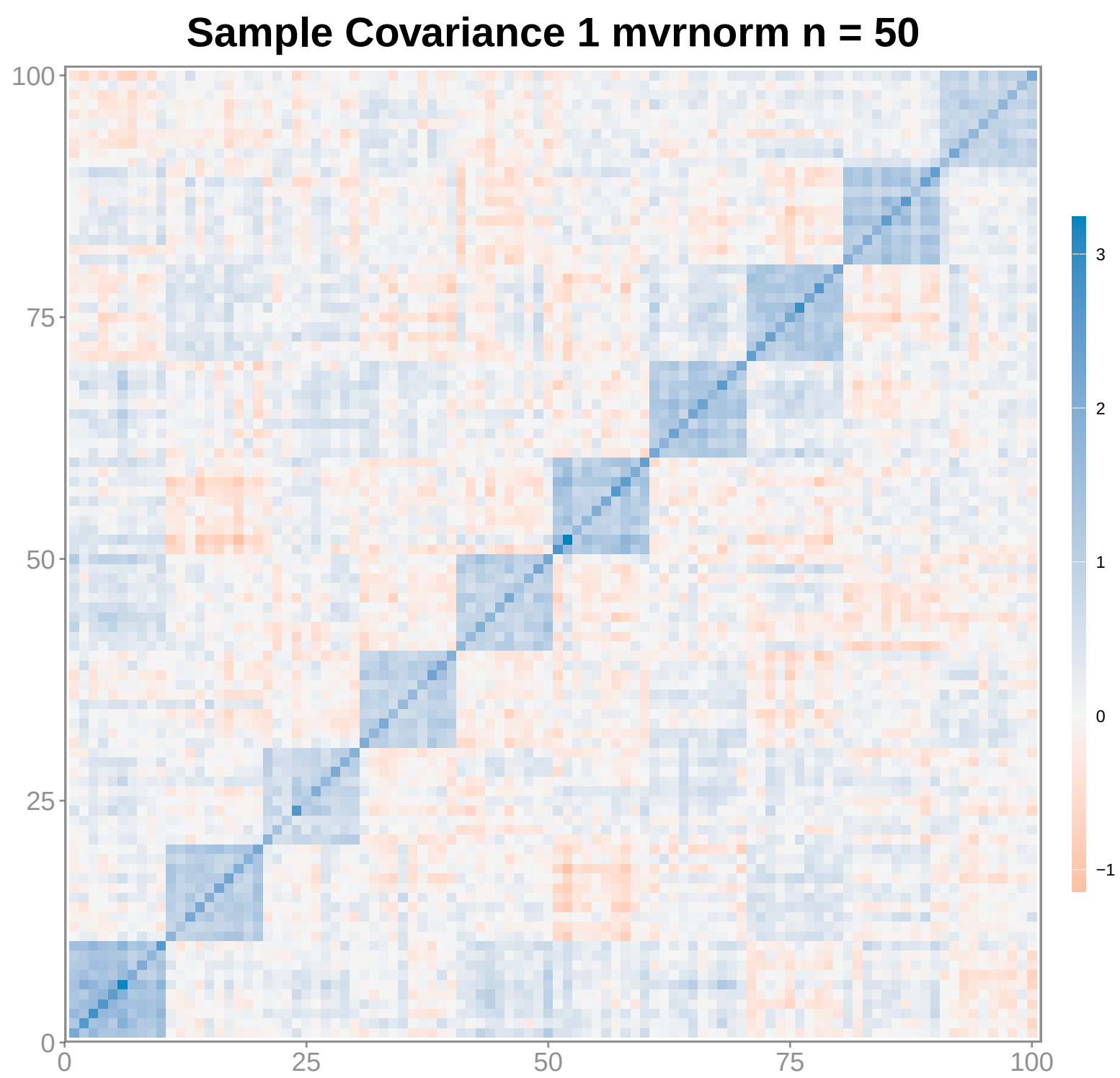

Supplementary Figure 11: Example sample covariance matrix S_1, generated with the sampling method mvrnorm with $n = 50$.

1.4 Evaluation

1.4.1 Binary Classification Metrics

$$\begin{aligned}
\mathrm{TP} &= |\{(j,k) : \Theta_{jk} \neq 0 \wedge \hat{\Theta}_{jk} \neq 0\}|, \\
\mathrm{TN} &= |\{(j,k) : \Theta_{jk} = 0 \wedge \hat{\Theta}_{jk} = 0\}|, \\
\mathrm{FP} &= |\{(j,k) : \Theta_{jk} = 0 \wedge \hat{\Theta}_{jk} \neq 0\}|, \\
\mathrm{FN} &= |\{(j,k) : \Theta_{jk} \neq 0 \wedge \hat{\Theta}_{jk} = 0\}|.
\end{aligned}$$

The F1 score takes values between 0, indicating the poorest performance, and 1, indicating perfect precision and recall, and is defined as:

$$\mathrm{F}_1 \text{ Score} = \frac{2 \cdot \mathrm{TP}}{2 \cdot \mathrm{TP} + \mathrm{FP} + \mathrm{FN}} \tag{1}$$

Accuracy measures the proportion of correctly classified instances among all predictions. It ranges from 0, indicating no correct classifications, to 1, indicating that all instances were classified correctly, and is defined as:

$$\text{Accuracy} = \frac{\mathrm{TP} + \mathrm{TN}}{\mathrm{TP} + \mathrm{FN} + \mathrm{TN} + \mathrm{FP}} \tag{2}$$

The Matthews correlation coefficient, originally proposed by B. W. Matthews (1975) [1], is a balanced measure for evaluating binary classification performance. It considers all four entries of the confusion matrix and returns a value between -1 and $+1$, where -1 indicates total disagreement between prediction and truth, 0 indicates random prediction, and $+1$ indicates perfect prediction. It is defined as:

$$\mathrm{MCC} = \frac{\mathrm{TP} \cdot \mathrm{TN} - \mathrm{FP} \cdot \mathrm{FN}}{\sqrt{(\mathrm{TP} + \mathrm{FP}) \cdot (\mathrm{TP} + \mathrm{FN}) \cdot (\mathrm{TN} + \mathrm{FP}) \cdot (\mathrm{TN} + \mathrm{FN})}} \tag{3}$$

To improve interpretability, a normalized version is used, rescaling the MCC to the interval $[0, 1]$, where 0 corresponds to the worst performance and 1 to the best:

$$\text{normMCC} = \frac{\mathrm{MCC} + 1}{2} \tag{4}$$

In cases where MCC is undefined due to division by zero, it is common to assign a value of 0 to MCC and 0.5 to normMCC, as justified by Chicco and Jurman (2020) [2].

1.4.2 ROC Curve Examples

[1] Matthews, B. W. (1975). Comparison of the predicted and observed secondary structure of T4 phage lysozyme. Biochimica et Biophysica Acta, 405(2), 442–451. `https://doi.org/10.1016/0005-2795(75)90109-9`

[2] Chicco, D., & Jurman, G. (2020). The advantages of the Matthews correlation coefficient (MCC) over F1 score and accuracy in binary classification evaluation. BMC Genomics, 21(1), 6. `https://doi.org/10.1186/s12864-019-6413-7`

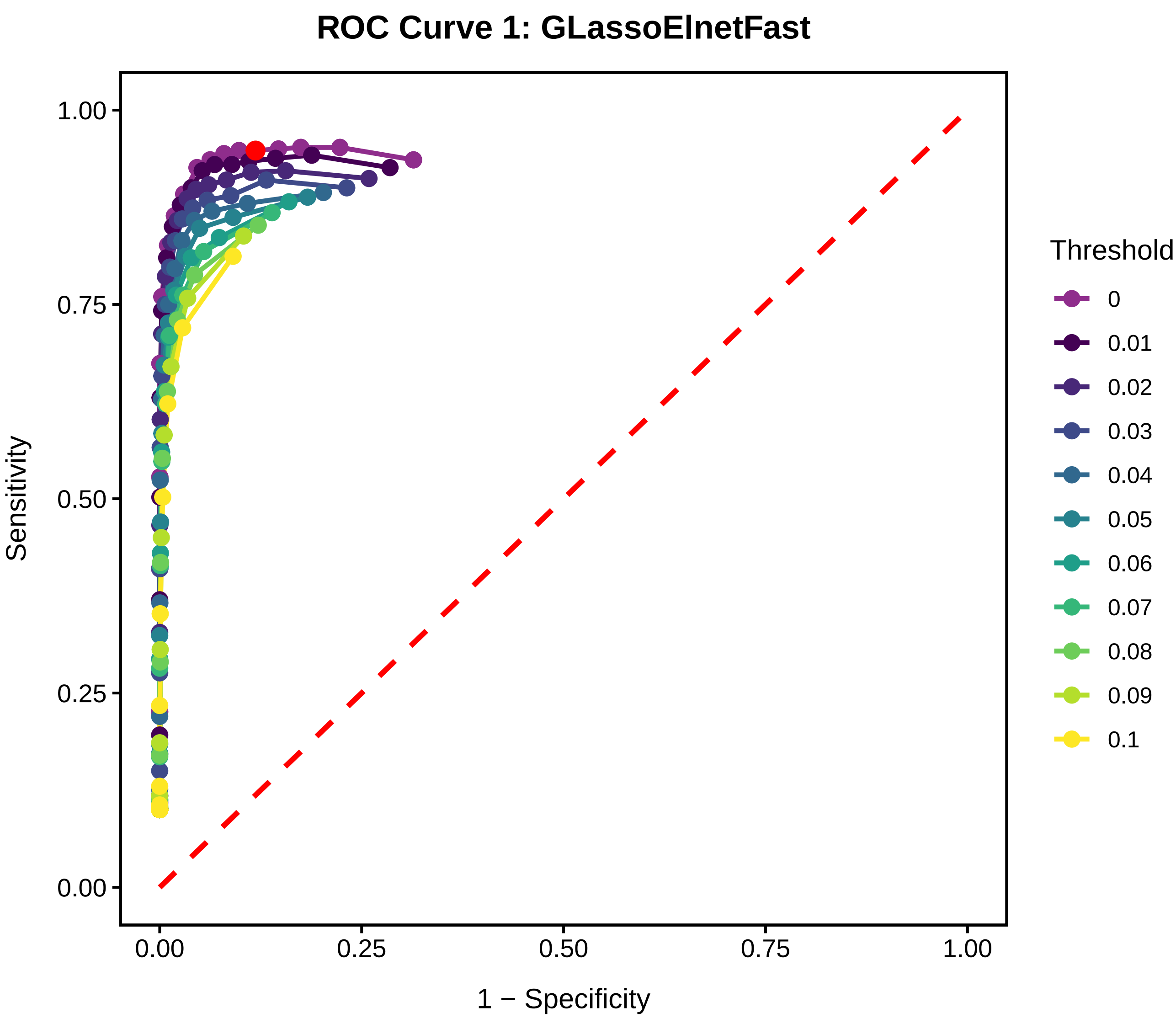

Supplementary Figure 12: Example ROC curve for estimated precision matrices obtained with *GLassoElnetFast* for condition one. Each coloured line represents one set of precision matrices, obtained by the PMEM with different regularization parameter values, with each point corresponding to one estimated precision matrix. A single precision matrix is selected as the final estimate, either via an internal regularization parameter selection or through external cross-validation. The sensitivity and 1-specificity of the selected precision matrix are indicated by a larger red dot. Different coloured lines correspond to different threshold levels applied to the estimated precision matrices. For each threshold, entries with absolute values below the threshold are set to zero, and the sensitivity and specificity of the resulting thresholded matrix are computed.

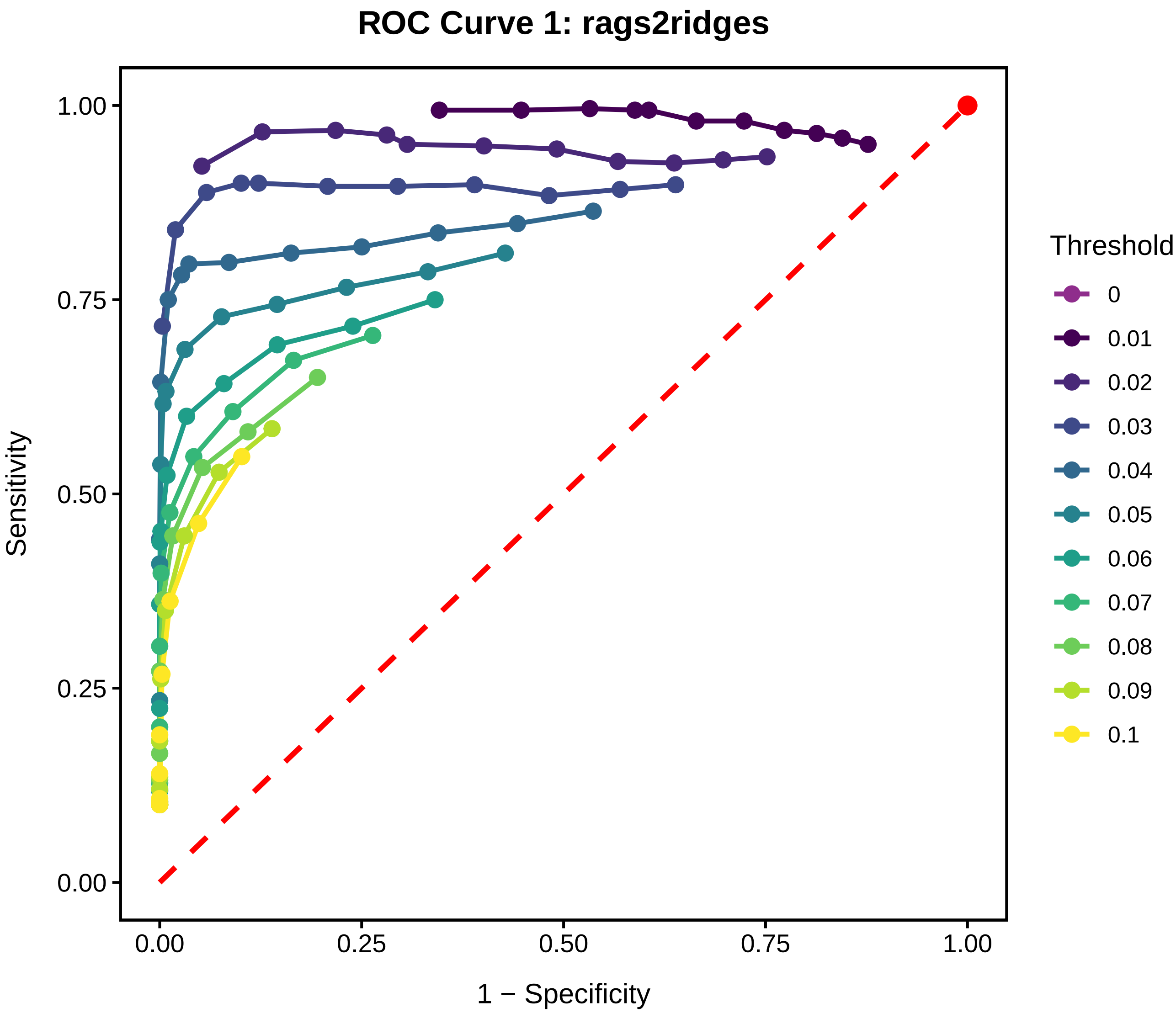

Supplementary Figure 13: Example ROC curve for estimated precision matrices obtained with *rags2ridges* for condition one. Each coloured line represents one set of precision matrices, obtained by the PMEM with different regularization parameter values, with each point corresponding to one estimated precision matrix. A single precision matrix is selected as the final estimate, either via an internal regularization parameter selection or through external cross-validation. The sensitivity and 1-specificity of the selected precision matrix are indicated by a larger red dot. Different coloured lines correspond to different threshold levels applied to the estimated precision matrices. For each threshold, entries with absolute values below the threshold are set to zero, and the sensitivity and specificity of the resulting thresholded matrix are computed.

2 Supplementary Information for Replicates

Since some methods required substantial computation time, especially when estimating high-dimensional precision matrices (e.g., several hours for $p = 500$), extensive replication was not always feasible. However, to obtain a general impression of baseline performance and consistency across methods, the PMEMs were first evaluated in 50 replicated simulations. Most PMEMs show stable performance across binary classification metrics, with the greatest variation observed in the F1 score (Figure S14 A). *GLassoElnetFast* achieves the highest values across all metrics, while *clime*, *rags2ridges*, and *rope* perform poorly, with normalized MCC values of 0.5 and reduced accuracy due to estimating fully dense precision matrices without thresholding. Matrix norm values vary only slightly for most PMEMs, with the Frobenius norm showing the largest spread (Figure S14 B). *Gdtrace* stands out with high variability across all norms, whereas *GLassoElnetFast* consistently yields the lowest values. Kullback-Leibler and reverse Kullback-Leibler losses show little variation between methods, with *GLassoElnetFast* again producing the lowest values (Figure S14 C). *Bigquic*, *scio*, and *tiger* consistently estimated empty precision matrices. *Bigquic* produced diagonal values fixed at 0.5, while *scio* and *tiger* showed varying diagonal entries ranging from 0.26 to 1.02 and 0.27 to 1.05, respectively. These outputs led to constant or near-constant results across all metrics, limiting their interpretability in this evaluation.

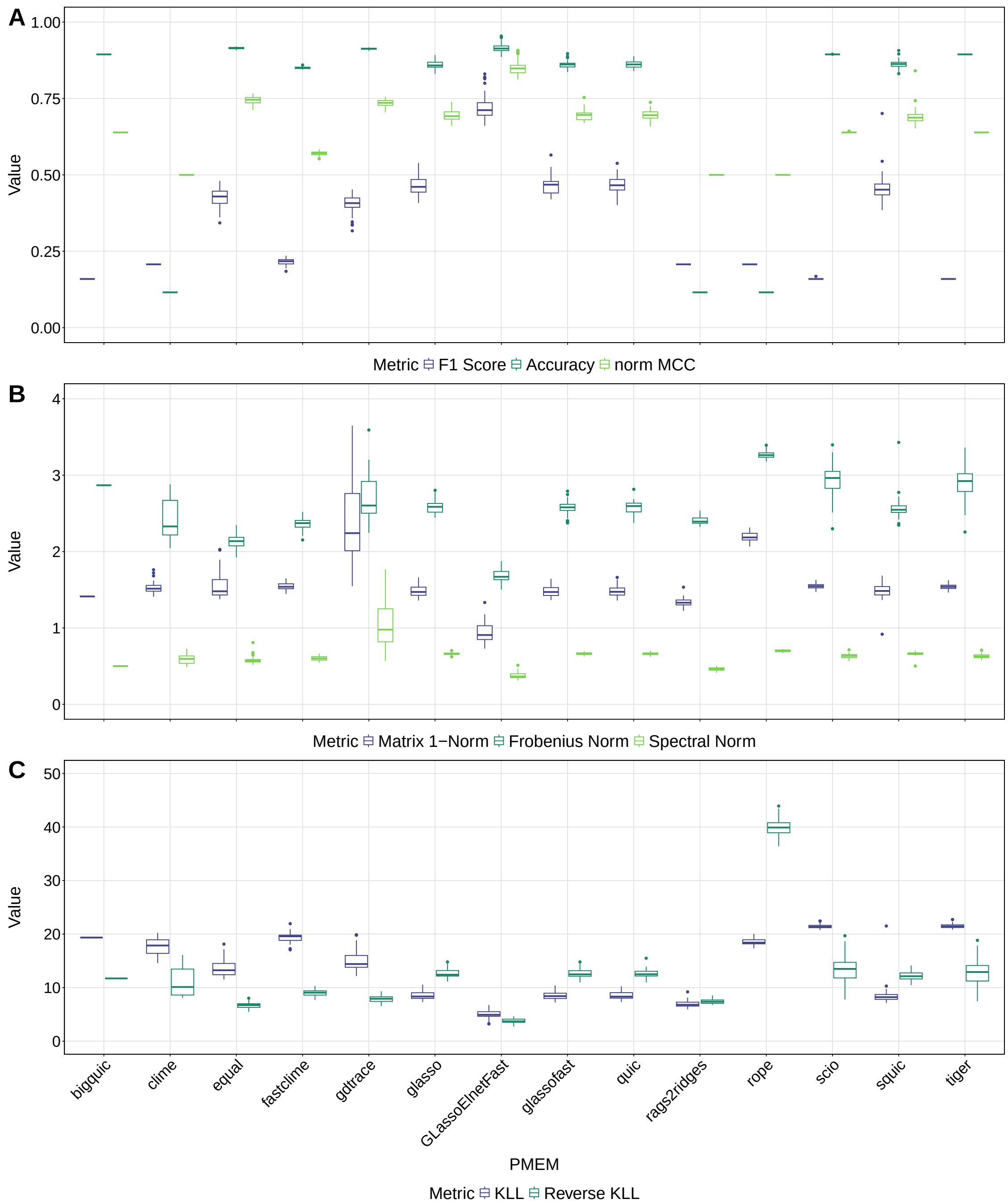

Supplementary Figure 14: Boxplot illustrating the reproducibility of different PMEMs in repeated simulations, assessed across the binary classification metrics (A): F1 score, accuracy, and normalized MCC, the matrix norms (B): matrix 1-norm, Frobenius norm, and spectral norm as well as the Kullback-Leibler Loss and the reverse Kullback-Leibler Loss (C). The performance of each method is represented by the distribution of the metric values over 50 replicates. Σ_1 was generated in each replicate with the single block method with $p = 100$. The sample data set was created using the mvrnorm sampling method with a sample size of 50. One outlier from *squic* (KLL ≈ 310) was excluded for clarity.

3 Supplementary Figures for Covariance 1 Methods

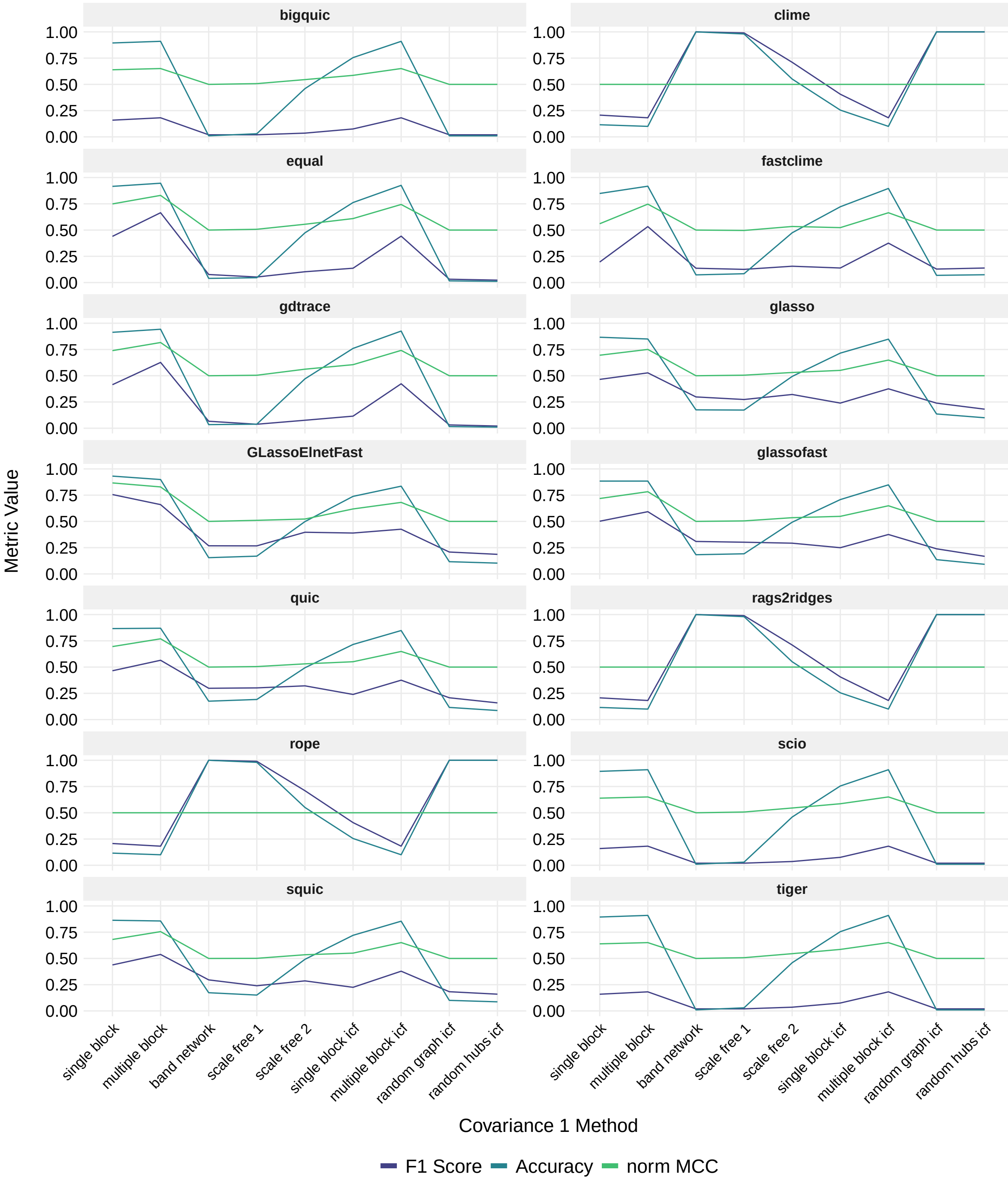

Supplementary Figure 15: Line plot illustrating the influence of different covariance 1 methods on the performances of precision matrix estimation methods, assessed across the binary classification metrics F1 score, accuracy, and normalized MCC for $\widehat{\Theta}_1$. Σ_1 was generated using the respective covariance 1 method with $p = 100$. The sample data set was created using the mvrnorm sampling method with $n = 50$.

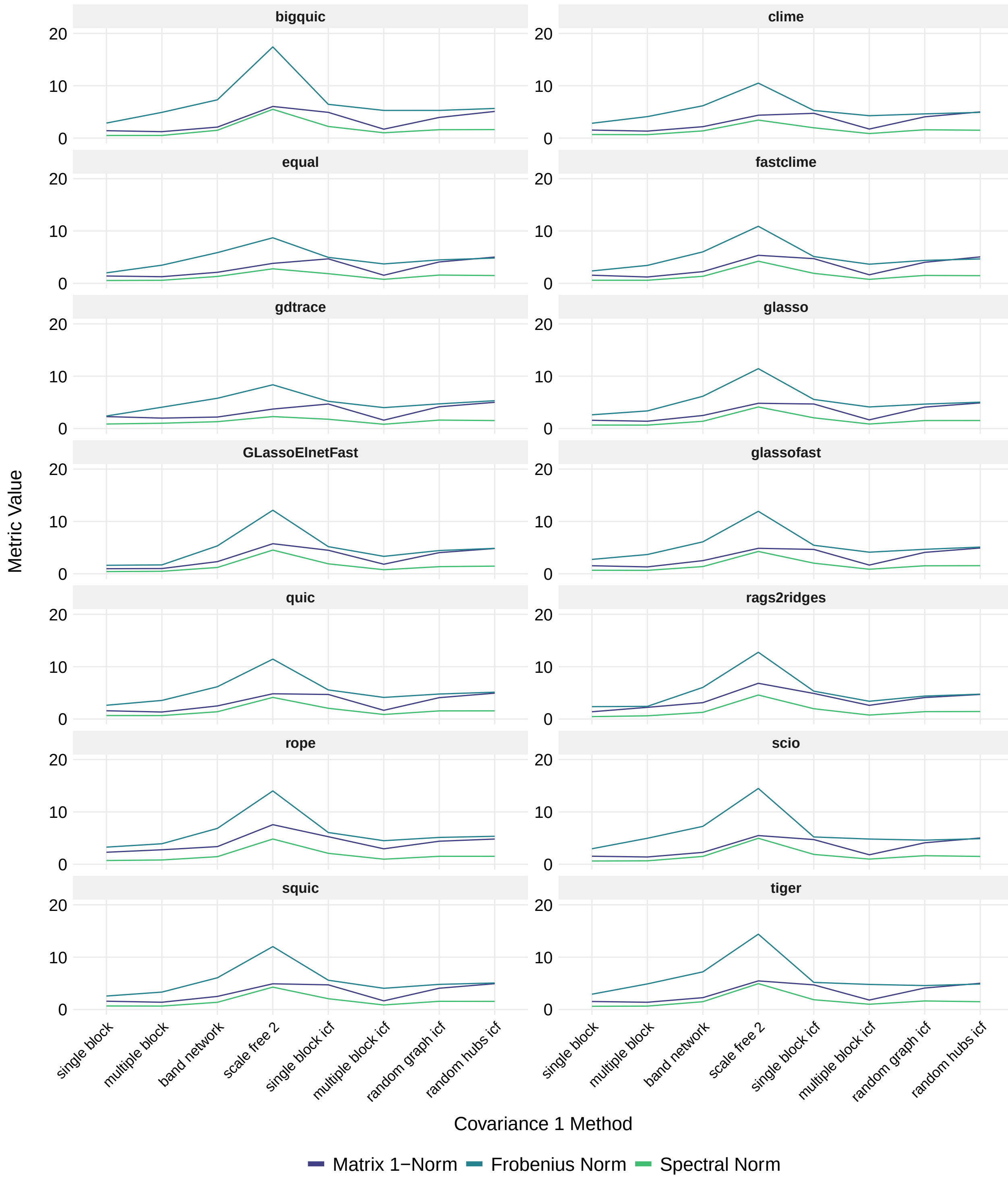

Supplementary Figure 16: Line plot illustrating the influence of different covariance 1 methods on the performances of precision matrix estimation methods, assessed across the matrix norms matrix 1-norm, Frobenius norm, and spectral norm for $\widehat{\Theta}_1$. Σ_1 was generated using the respective covariance 1 method with $p = 100$. The sample data set was created using the mvrnorm sampling method with $n = 50$. The results of the covariance 1 method scale free 1 were excluded in the figure. The matrix 1-norm was 46985054, the Frobenius norm was 33520059, and the spectral norm was 13684507 for all PMEMs in this approach.

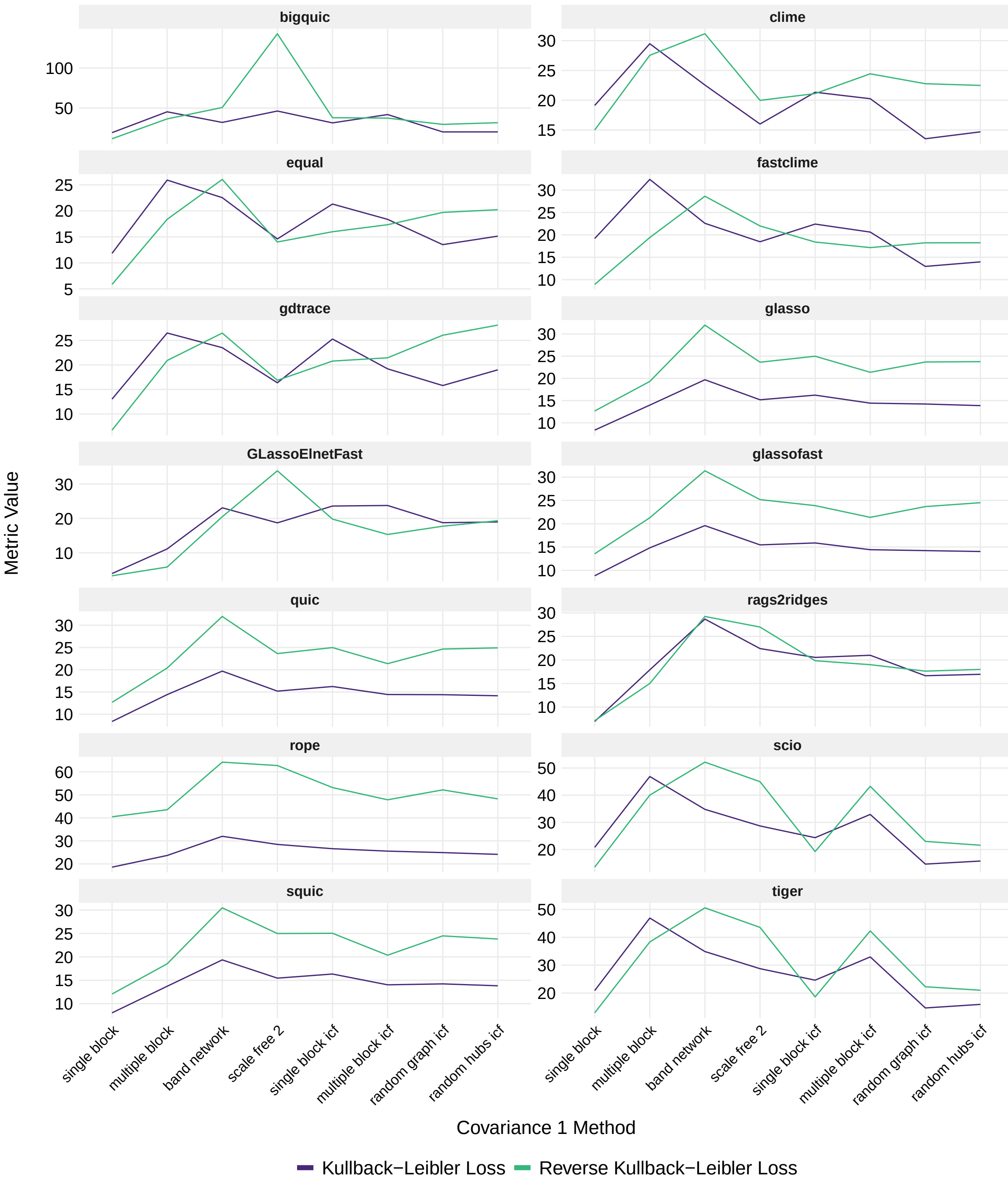

Supplementary Figure 17: Line plot illustrating the influence of different covariance 1 methods on the performances of precision matrix estimation methods, assessed across the Kullback-Leibler Loss and the reverse Kullback-Leibler Loss for $\widehat{\Theta}_1$. Σ_1 was generated using the respective covariance 1 method with $p = 100$. The sample data set was created using the mvrnorm sampling method with $n = 50$.

4 Supplementary Figures for Covariance Density

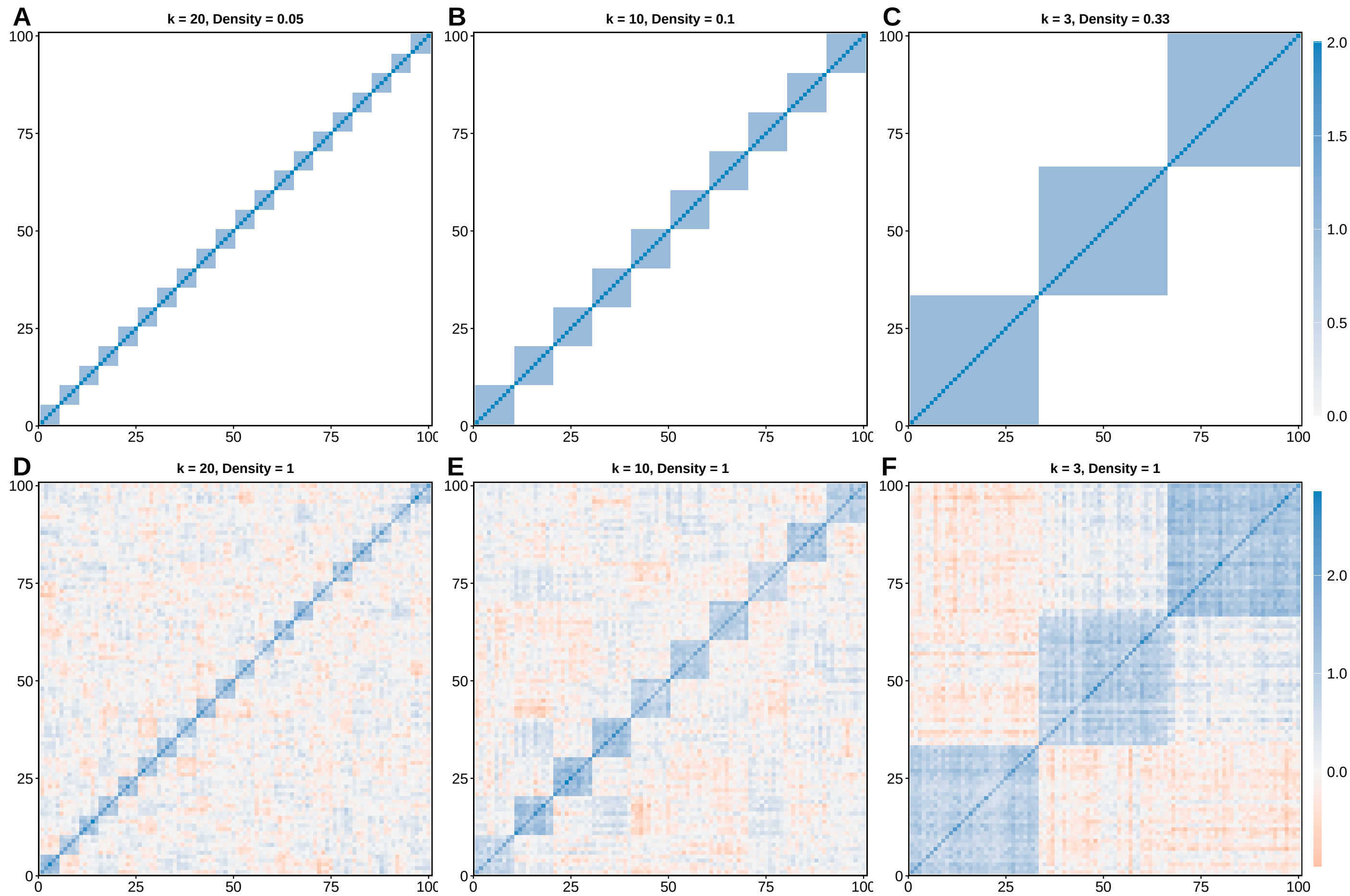

Supplementary Figure 18: A-C: Σ_1 generated using covariance 1 method multiple block with k blocks of non-zero off-diagonal values. D-F: S_1 generated by the mvrnorm method based on the respective Σ_1 in A-C.

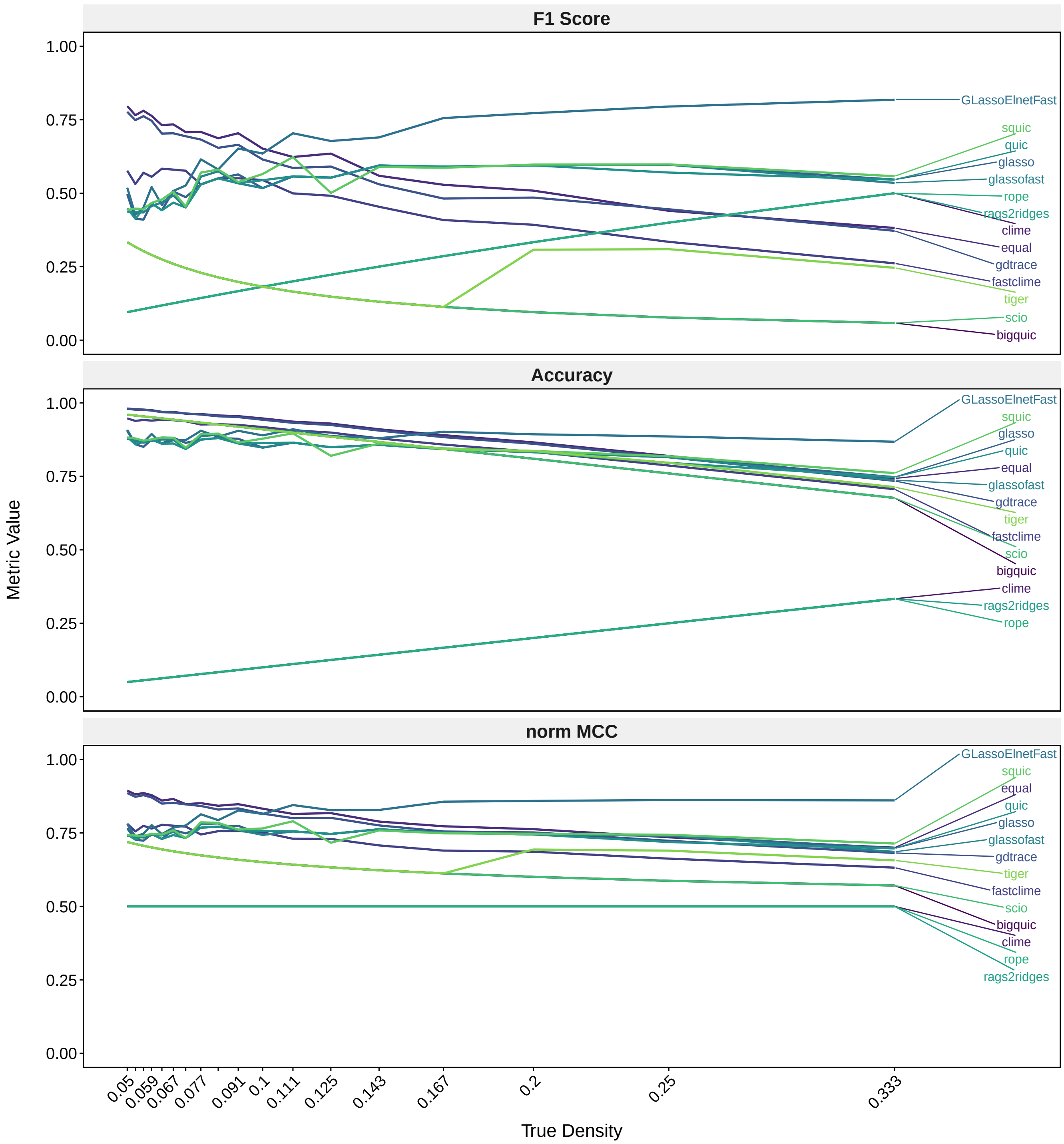

Supplementary Figure 19: Line plot illustrating the influence of different Θ_1 densities on the performances of precision matrix estimation methods, assessed across the binary classification metrics F1 score, accuracy, and normalized MCC. Densities from 0.05 to 0.33 were analysed. Σ_1 was generated using the covariance 1 method multiple block with $p = 100$. The different densities for Θ_1 were achieved by varying the number of non-zero blocks within Σ_1. The sample data set was created using the sampling method mvrnorm with $n = 50$.

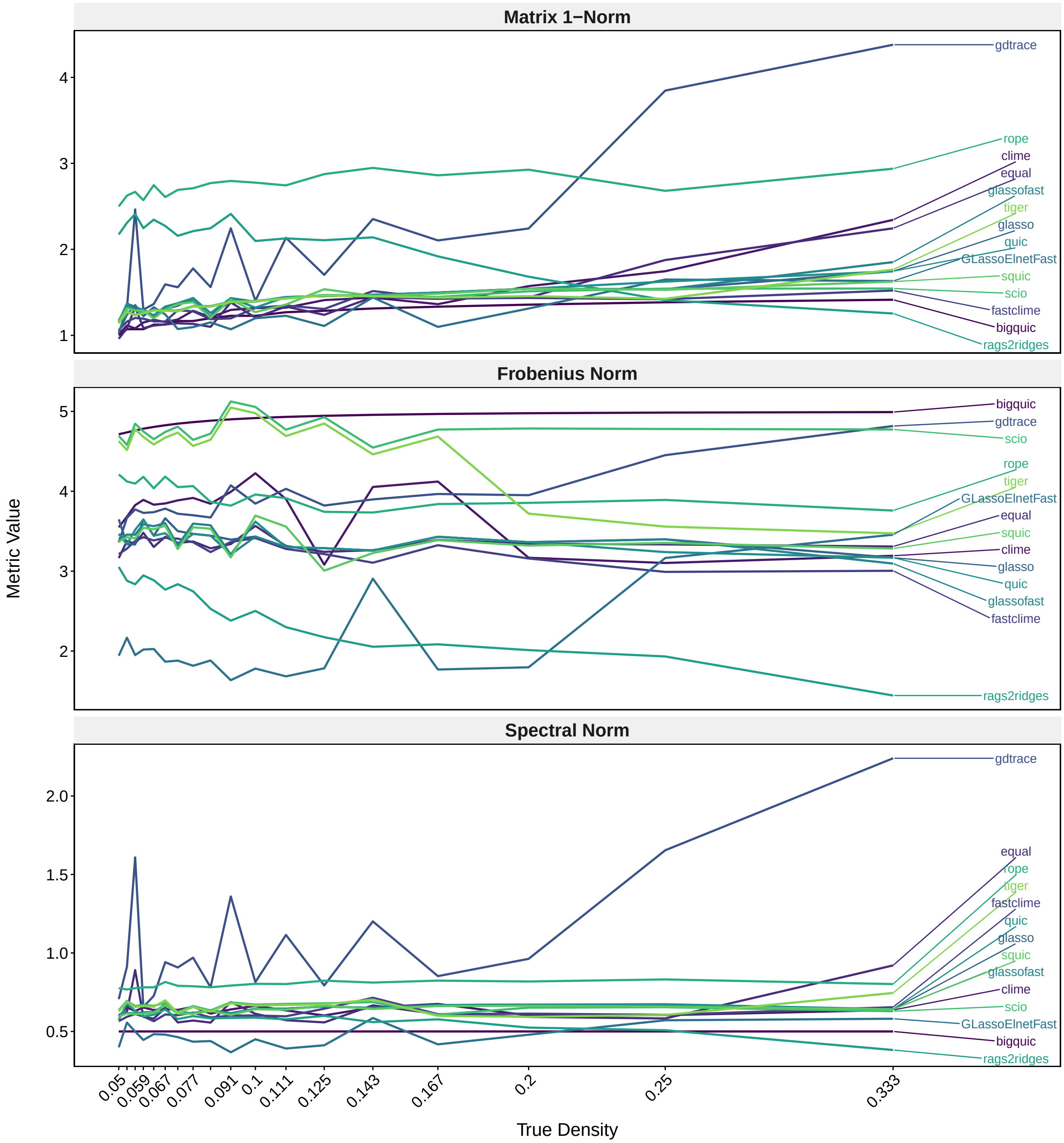

Supplementary Figure 20: Line plot illustrating the influence of different Θ_1 densities on the performances of precision matrix estimation methods, assessed across the matrix norms matrix 1-norm, Frobenius norm, and spectral norm. Densities from 0.05 to 0.33 were analysed. Σ_1 was generated using the covariance 1 method multiple block, using $p = 100$. The different densities for Θ_1 were achieved by varying the number of non-zero blocks within Σ_1. The sample data set was created using the sampling method mvrnorm with $n = 50$.

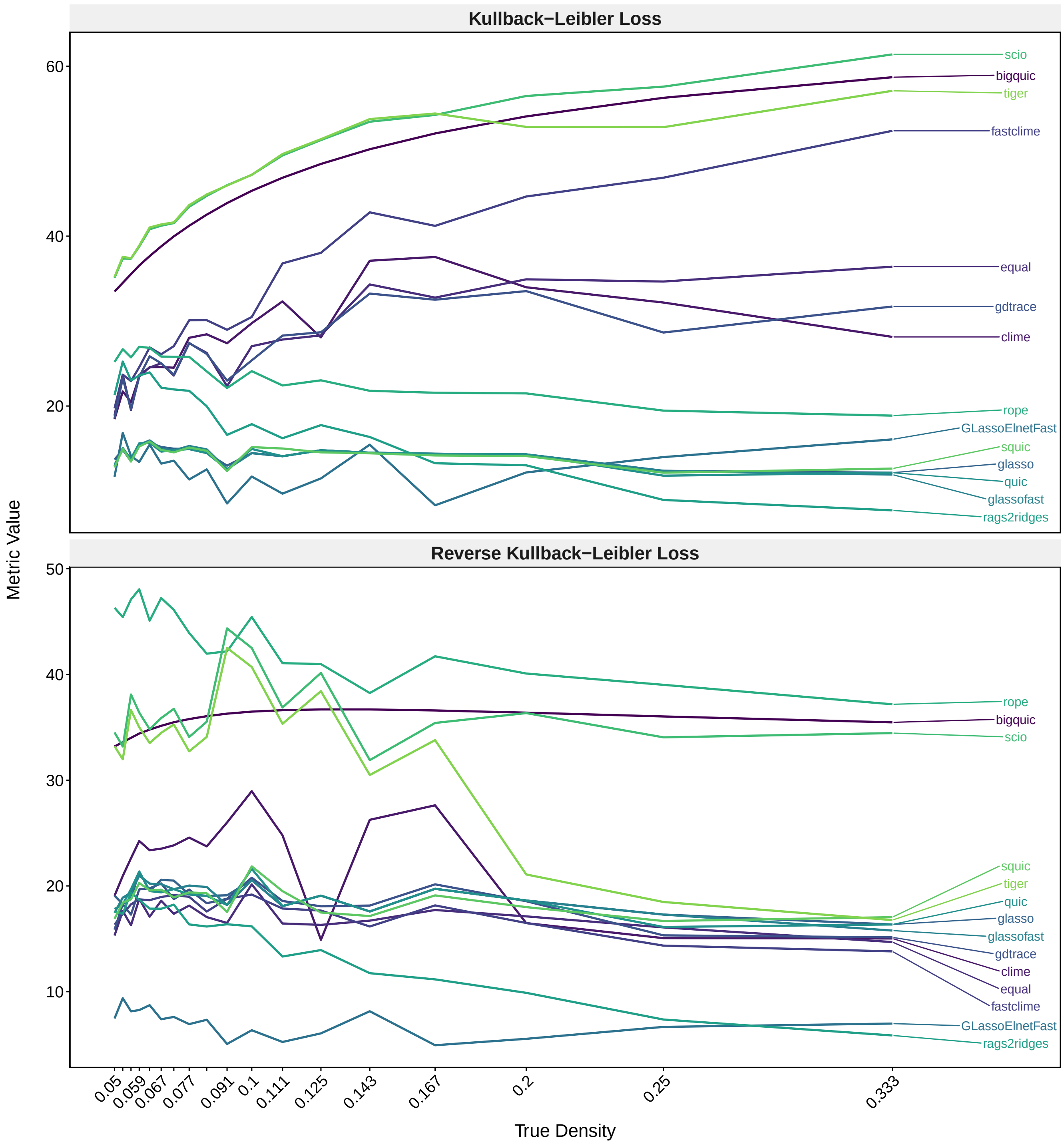

Supplementary Figure 21: Line plot illustrating the influence of different Θ_1 densities on the performances of precision matrix estimation methods, assessed across the Kullback-Leibler Loss and the reverse Kullback-Leibler Loss. Densities from 0.05 to 0.33 were analysed. Σ_1 was generated using the covariance 1 method multiple block, using $p = 100$. The different densities for Θ_1 were achieved by varying the number of non-zero blocks within Σ_1. The sample data set was created using the sampling method mvrnorm with $n = 50$.

5 Supplementary Information for Covariance 2 Methods

To assess how the method of covariance alteration influences the performance of the PMEMs, the second condition was simulated using either the knockout or mutate approach to generate Σ_2, from which the corresponding true precision matrix Θ_2 was derived (see Methods: Covariance Matrix Alteration). The estimated precision matrices $\widehat{\Theta}_2$ were then evaluated against Θ_2 (Supplementary Figures 22, 23, 24, and 25). *Bigquic*, *scio*, and *tiger* again estimated completely empty matrices for $\widehat{\Theta}_2$. *Bigquic* produced diagonal entries fixed at 0.5, while *scio* and *tiger* yielded diagonal values ranging from 0.136 to 1.277 and 0.139 to 1.303, respectively. As before, *clime*, *rags2ridges*, and *rope* returned dense matrices, limiting the interpretability of binary classification metrics without additional thresholding.

The binary classification metrics indicate reduced performance for all PMEMs in the mutate approaches (Figure S22). *Clime*, *rope*, and *rags2ridges* appear to perform better in terms of F1 score and accuracy. It is important to note that in these approaches, the knockout method generated Θ_2 with a density similar to Θ_1, consistently around 0.08, while the mutate method resulted in considerably higher and more variable densities, ranging from 0.30 to 1.00. While this benefits the PMEMs *clime*, *rags2ridges*, and *rope* in F1 and accuracy, normalized MCC remains low. Matrix norm values mostly increased from knockout to mutate (Figure S23), although the Frobenius norm occasionally decreased depending on the PMEM. Kullback-Leibler and reverse Kullback-Leibler losses also tended to increase under the mutate condition (Figure S24). Differential edge recovery results are shown in Supplementary Figure S25. The highest recovery rates were achieved by *GLassoElnetFast* and *rags2ridges* in the knockout approaches. While *GLassoElnetFast* showed reduced recovery in the mutate approaches, performing similarly to *glasso*, *glassofast*, *quic*, and *squic*. *Rags2ridges* maintained higher recovery and achieved the best results across all PMEMs. *Bigquic*, *scio*, and *tiger* did not recover any differential edges, consistent with their empty matrix estimates.

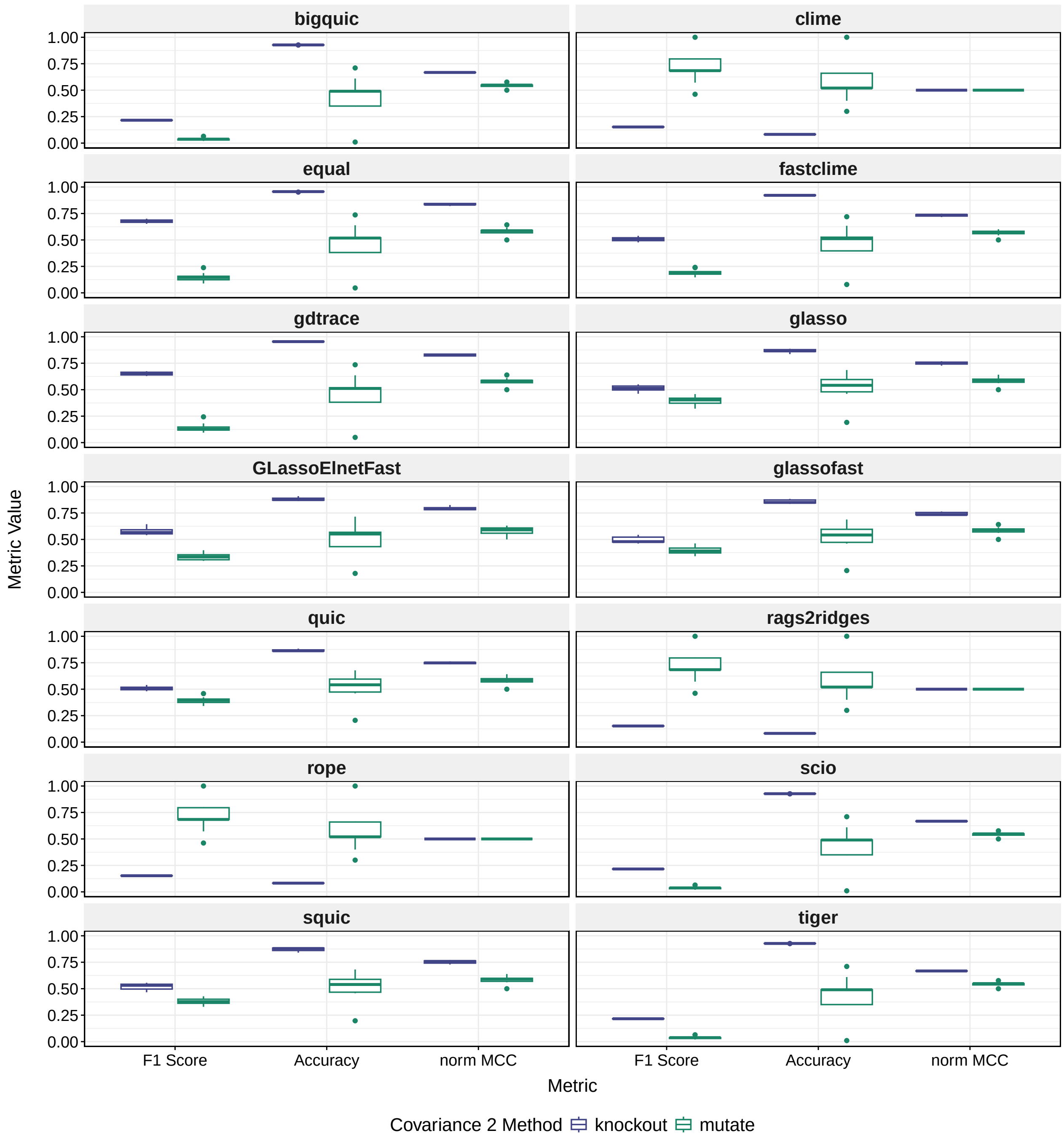

Supplementary Figure 22: Boxplot illustrating the influence of different covariance 2 methods on the performances of precision matrix estimation methods, assessed across the binary classification metrics F1 score, accuracy, and normalized MCC for $\widehat{\Theta}_2$. The performance of each method is represented by the distribution of the metric values over 10 replicates for each covariance 2 method. Σ_1 was generated using multiple block with $p = 100$. The sample data set was created using the mvrnorm sampling method with $n = 50$.

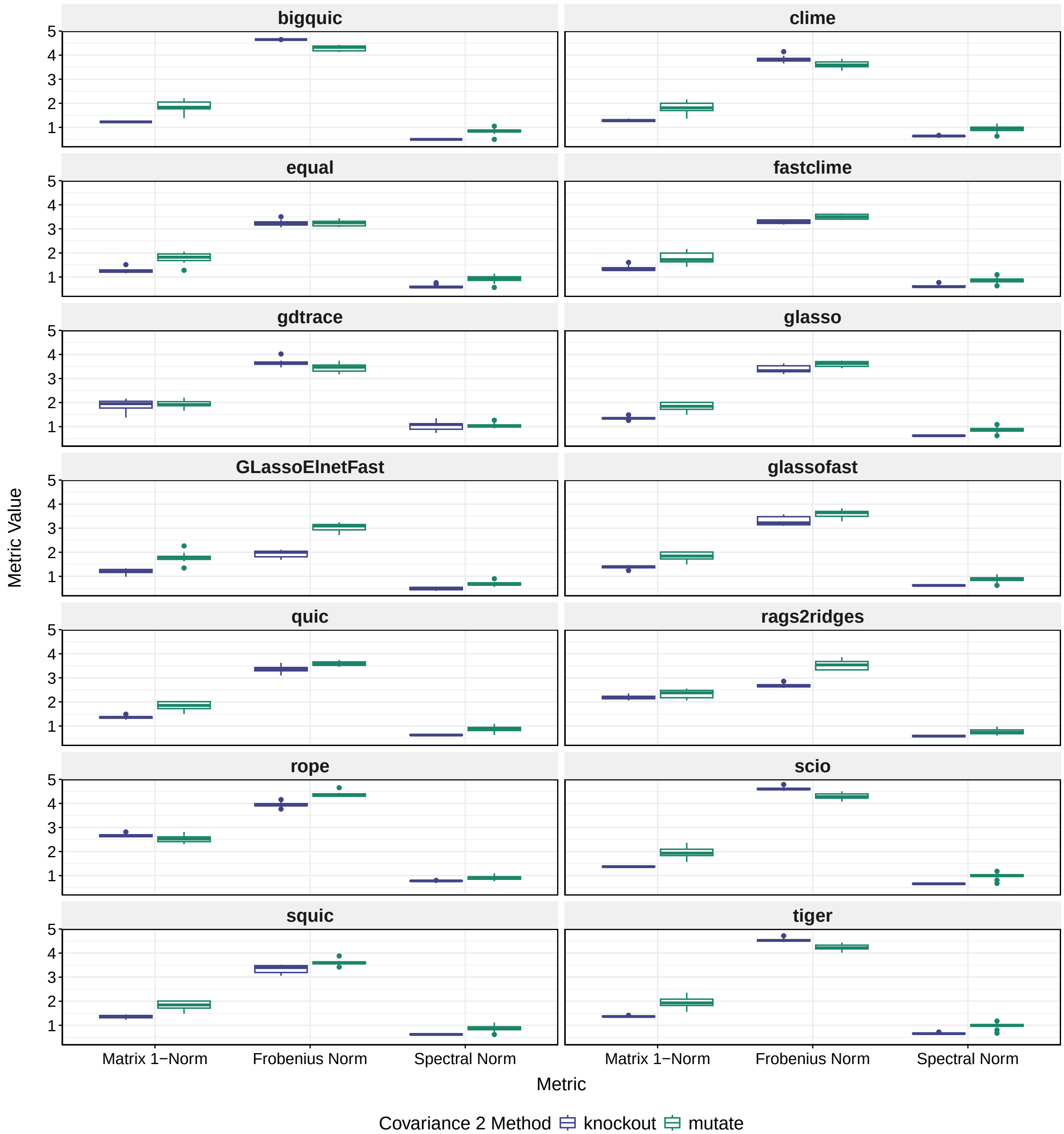

Supplementary Figure 23: Boxplot illustrating the influence of different covariance 2 methods on the performances of precision matrix estimation methods, assessed across the matrix norms matrix 1-norm, Frobenius norm, and spectral norm for $\widehat{\Theta}_2$. The performance of each method is represented by the distribution of the metric values over 10 replicates for each covariance 2 method. Σ_1 was generated using multiple block with $p = 100$. The sample data set was created using the mvrnorm sampling method with $n = 50$.

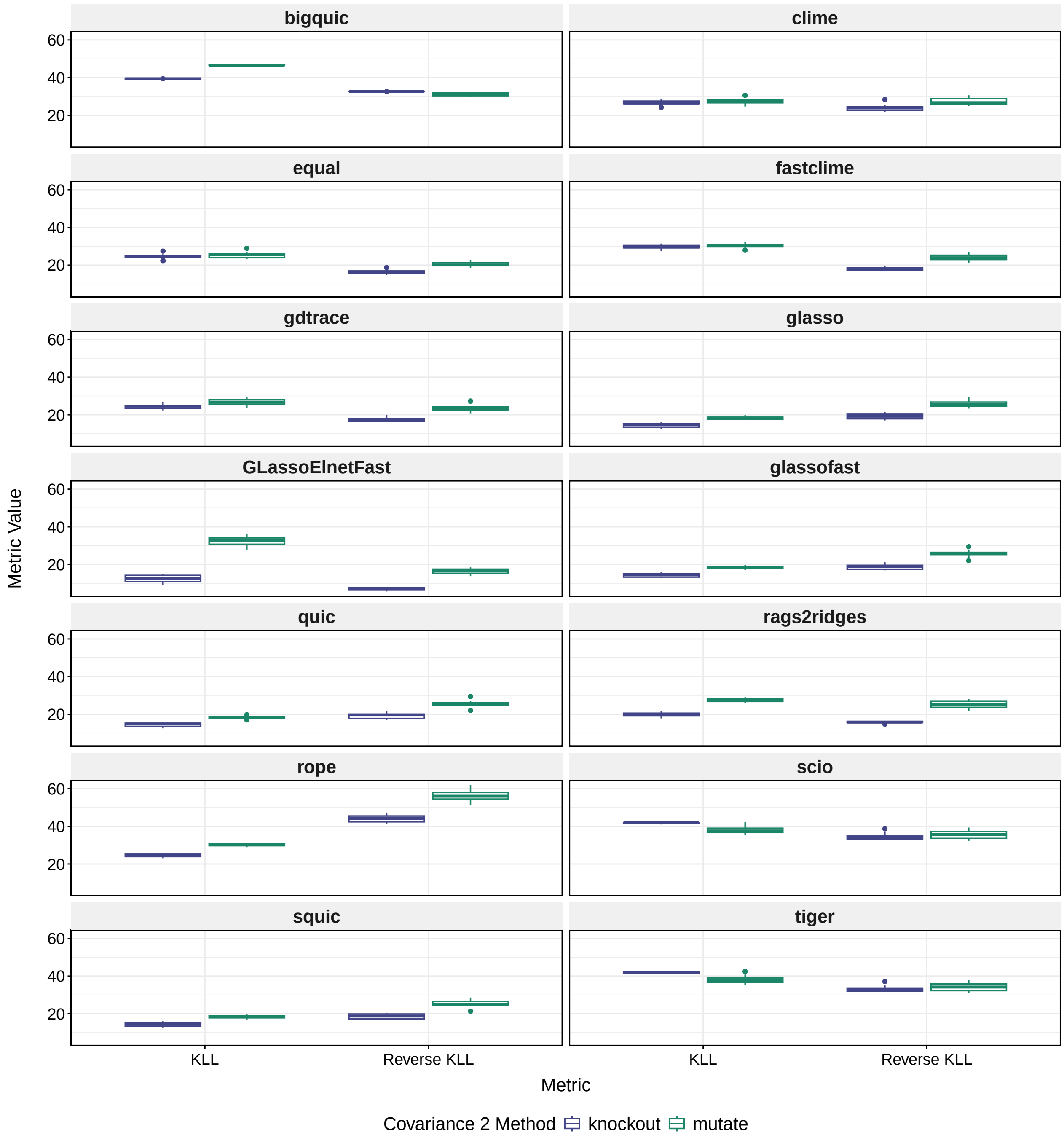

Supplementary Figure 24: Boxplot illustrating the influence of different covariance 2 methods on the performances of precision matrix estimation methods, assessed across the Kullback-Leibler Loss and the reverse Kullback-Leibler Loss for $\widehat{\Theta}_2$. The performance of each method is represented by the distribution of the metric values over 10 replicates for each covariance 2 method. Σ_1 was generated using multiple block with $p = 100$. The sample data set was created using the mvrnorm sampling method with $n = 50$.

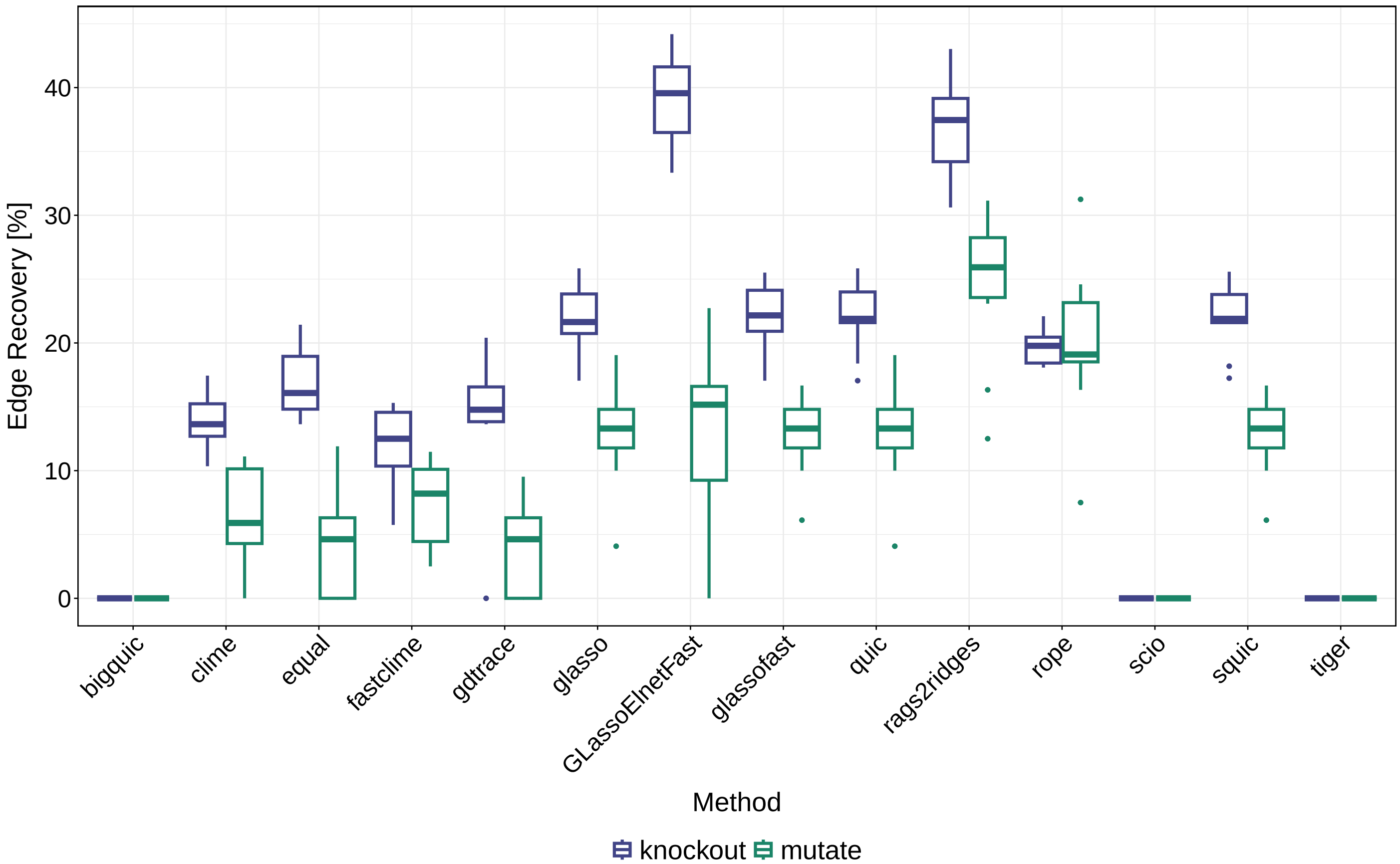

Supplementary Figure 25: Boxplot illustrating the influence of different covariance 2 methods on the performances of precision matrix estimation methods, assessed across by differential edge recovery rates. The performance of each method is represented by the distribution of the metric values over 10 replicates for each covariance 2 method. Σ_1 was generated using multiple block with $p = 100$. The sample data set was created using the mvrnorm sampling method with $n = 50$.

6 Supplementary Figures for Covariance Increase

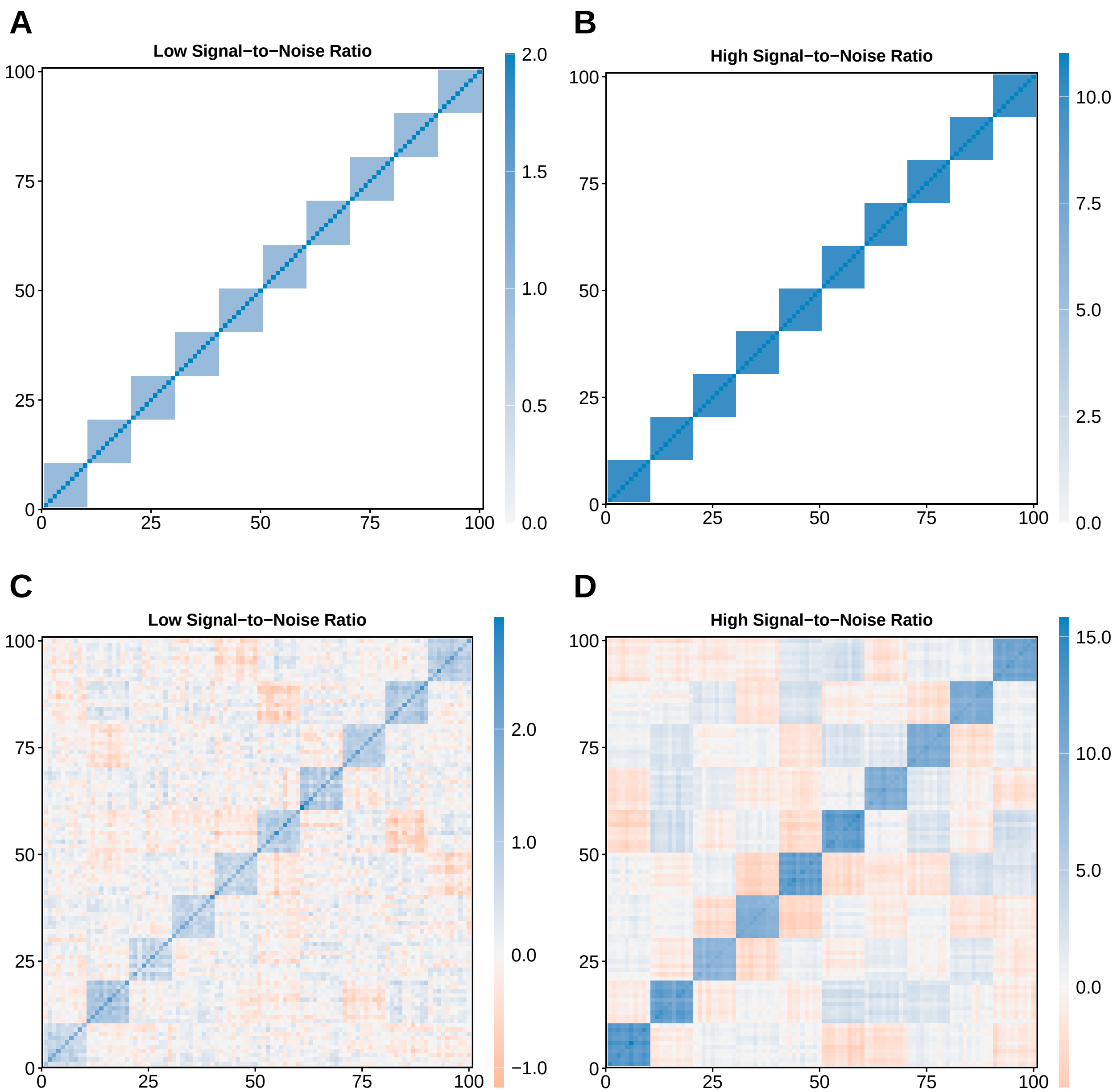

Supplementary Figure 26: A & B: Σ_1 generated with multiple block method using covariances of 1 or 10 and variances 2 or 11, respectively. C & D: S_1 generated with mvrnorm based on the Σ_1 in A & B.

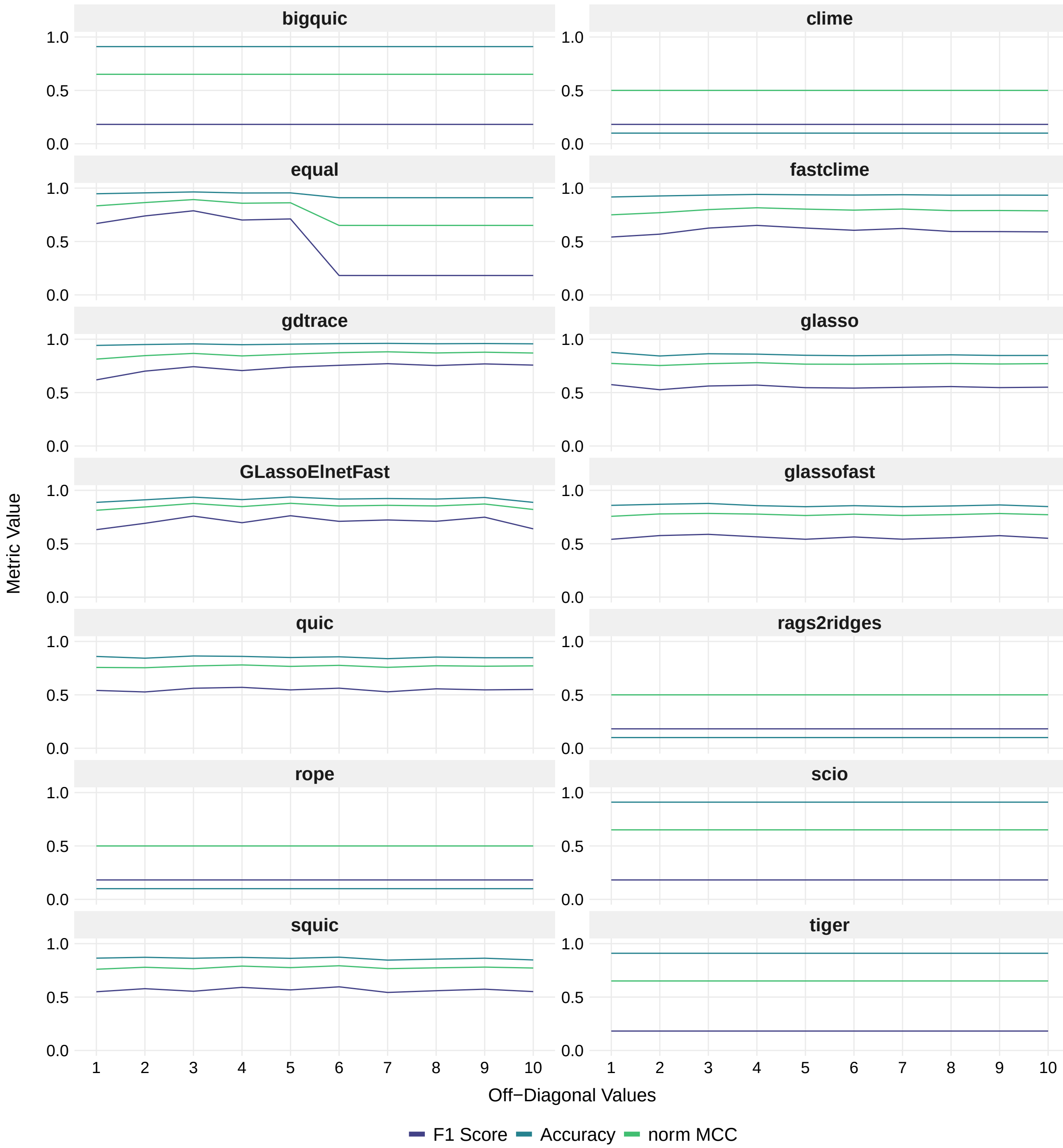

Supplementary Figure 27: Line plot illustrating the influence of different off-diagonal values (covariances) on the performances of precision matrix estimation methods, assessed across the binary classification metrics F1 score, accuracy, and normalized MCC. The covariances in Σ_1 and thus also Σ_2 were varied, ranging from 1 to 10. The diagonal values (variances) were set from 2 to 11, respectively. Σ_1 was generated using the covariance 1 method multiple block with $p = 100$. Σ_2 was generated based on Σ_1 using the covariance 2 method knockout. The simulated data sets were created with the sampling method mvrnorm with $n = 50$.

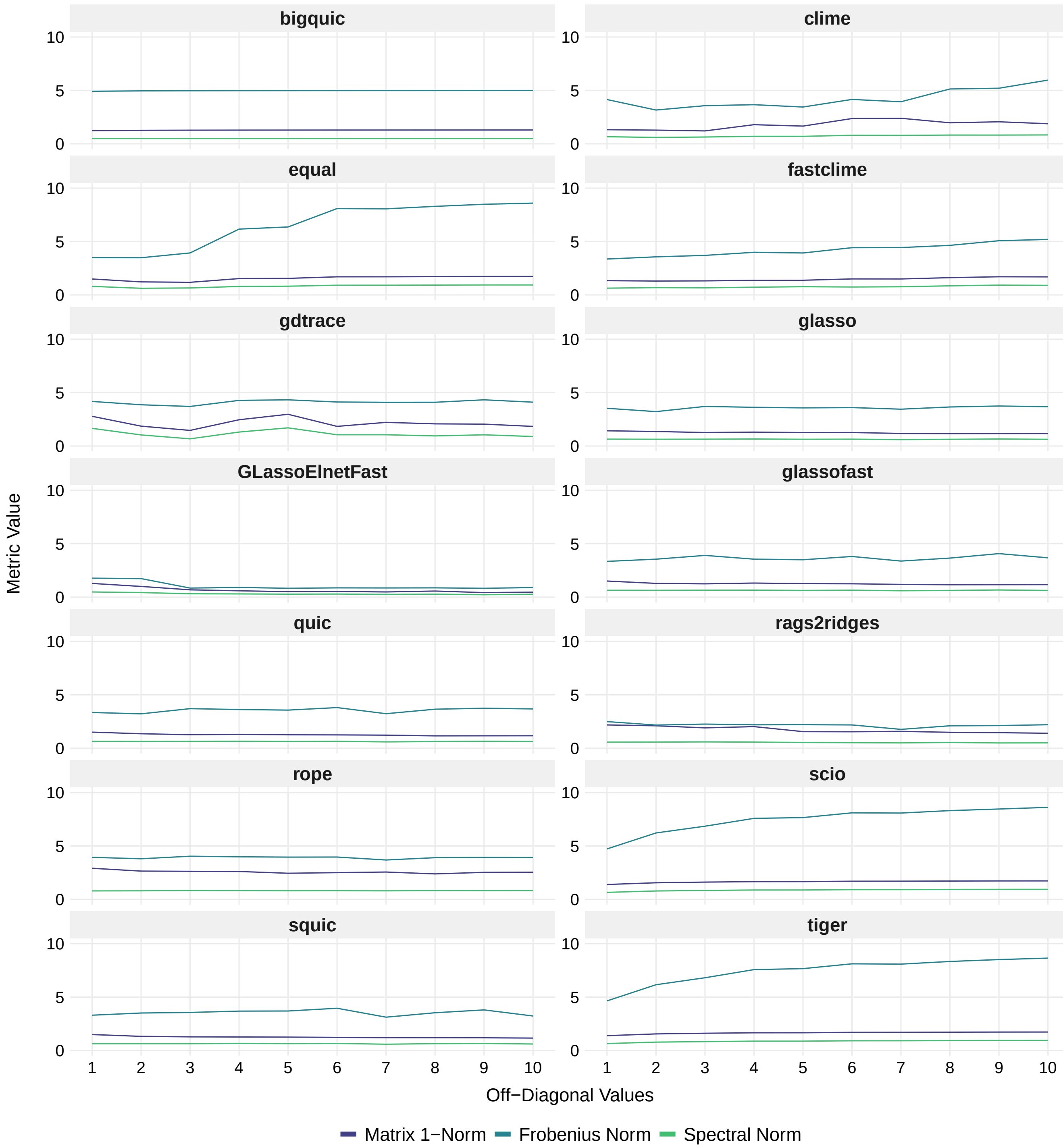

Supplementary Figure 28: Line plot illustrating the influence of different off-diagonal values (covariances) on the performances of precision matrix estimation methods, assessed across the matrix norms matrix 1-norm, Frobenius norm, and spectral norm. The covariances in Σ_1 and thus also Σ_2 were varied, ranging from 1 to 10. The diagonal values (variances) were set from 2 to 11, respectively. Σ_1 was generated using the covariance 1 method multiple block with $p = 100$. Σ_2 was generated based on Σ_1 using the covariance 2 method knockout. The simulated data sets were created with the sampling method mvrnorm with $n = 50$.

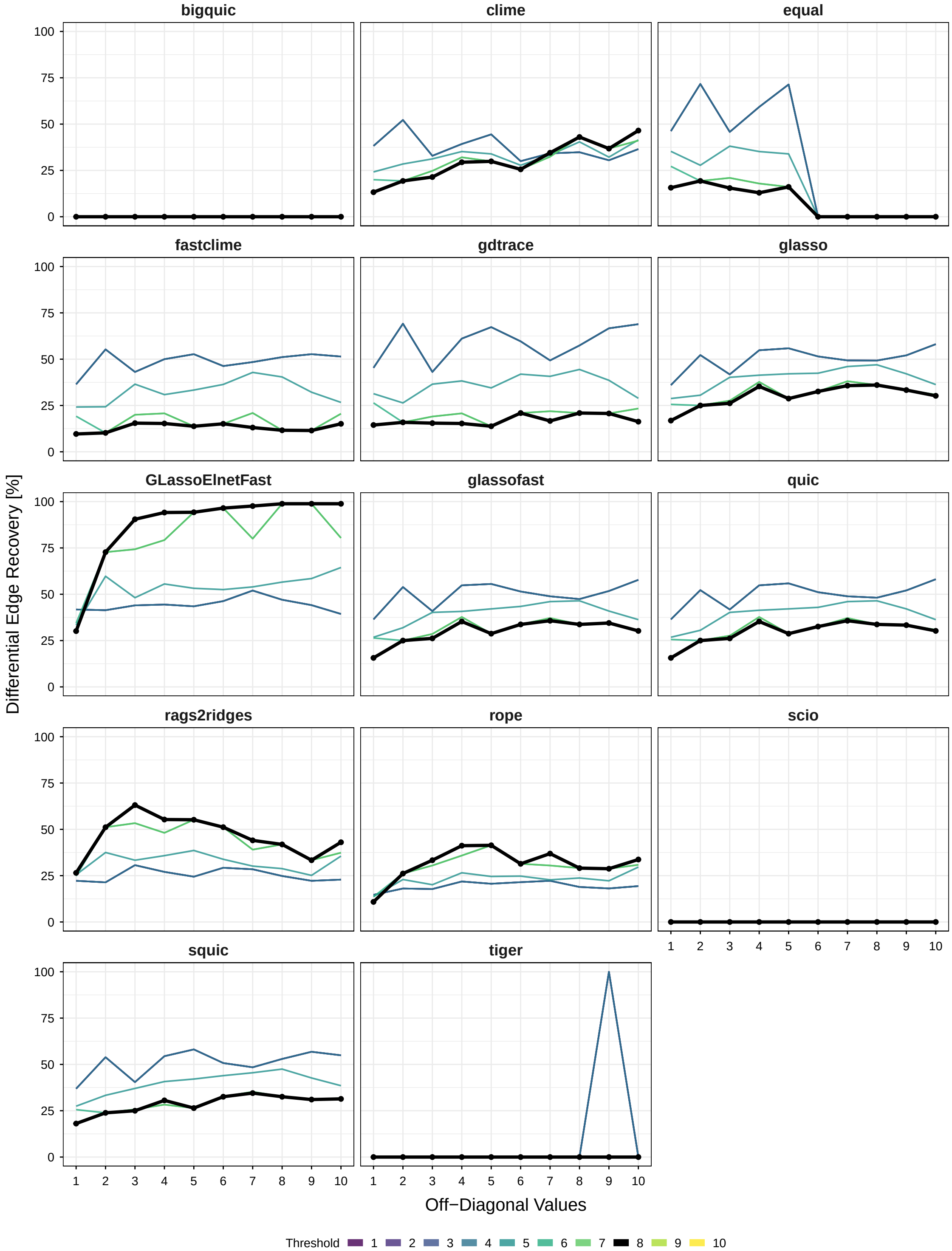

Supplementary Figure 29: Line plot illustrating the sensitivity of the Differential Edge Recovery (DER) score, computed on $\widehat{\Psi}_\Delta$, to the weight threshold τ across different off-diagonal values (covariances). Within each panel, DER is shown across off-diagonal values for each of the $m = 10$ threshold values $\tau_1, \ldots, \tau_{10}$ generated as described in the Method section. The threshold used for the corresponding main-text figure is highlighted in black. The covariances in Σ_1 and thus also Σ_2 were varied, ranging from 1 to 10. The diagonal values (variances) were set from 2 to 11, respectively. Σ_1 was generated using the covariance 1 method multiple block with $p = 100$. Σ_2 was generated based on Σ_1 using the covariance 2 method knockout. The simulated data sets were created with the sampling method mvrnorm with $n = 50$.

7 Supplementary Figures for Dimensions

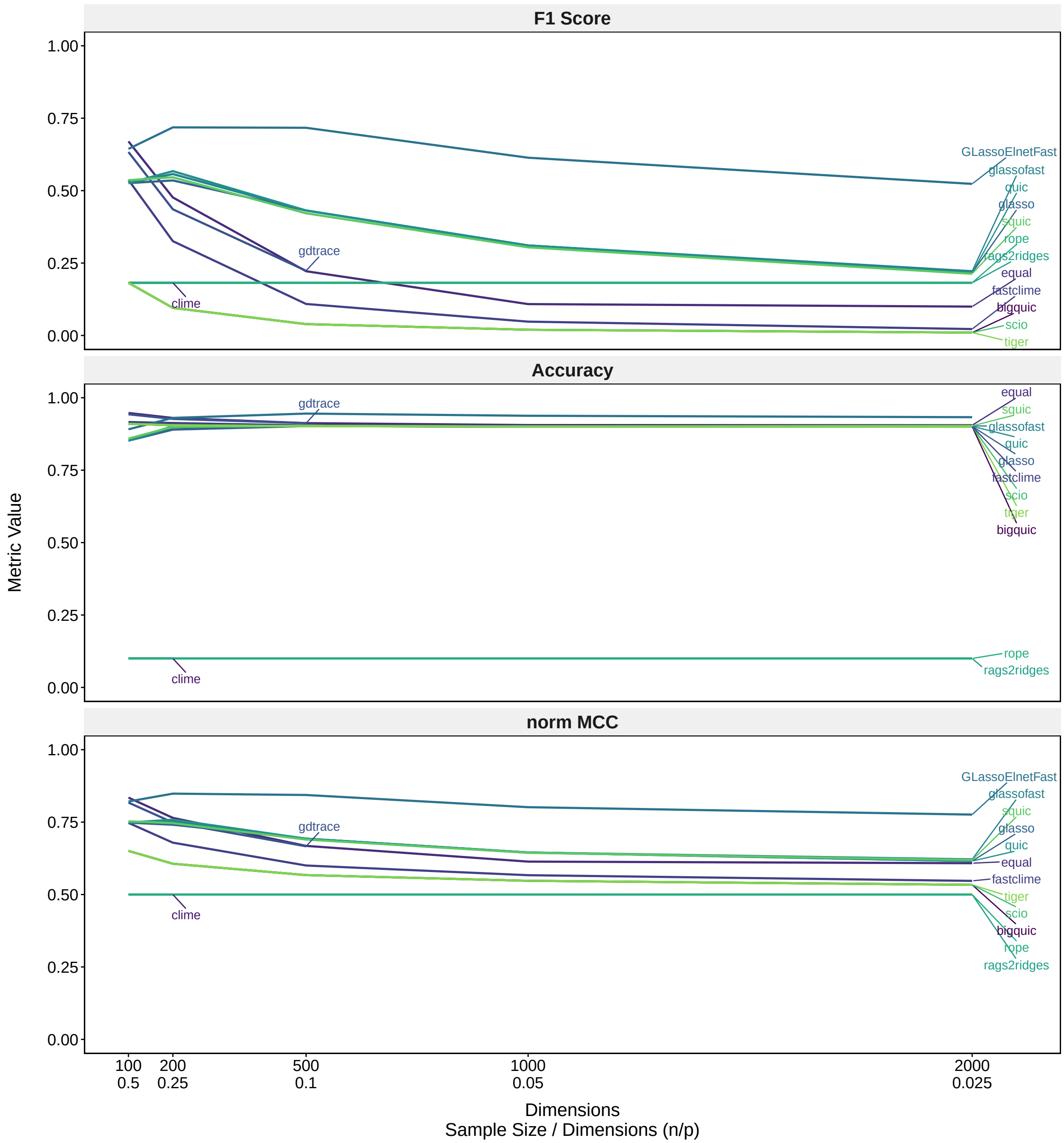

Supplementary Figure 30: Line plot illustrating the impact of increasing the dimensions on the performances of precision matrix estimation methods, assessed across the binary classification metrics F1 score, accuracy, and normalized MCC for $\widehat{\Theta}_1$. Dimensions from 100 to 2000 were analysed. Σ_1 was generated using the covariance 1 method multiple block, using $p = 100, 200, 500, 1000, 2000$. The sample data sets were created using the sampling method mvrnorm with $n = 50$. *Clime* and *gdtrace* were only analysed in the approaches with $p = 100$ and $p = 100, 200$, respectively.

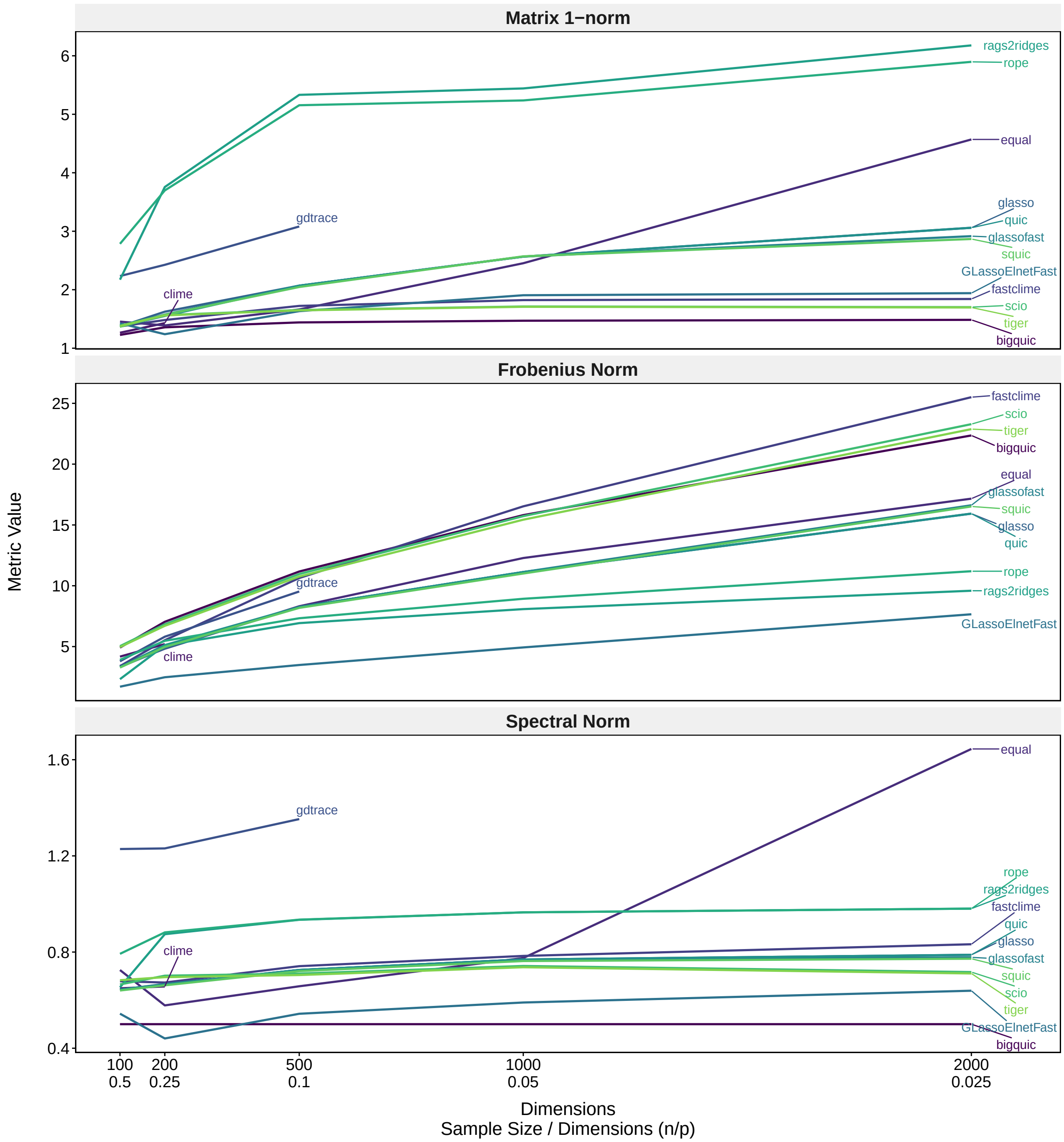

Supplementary Figure 31: Line plot illustrating the impact of increasing the dimensions on the performances of precision matrix estimation methods, assessed across the matrix norms matrix 1-norm, Frobenius norm, and spectral norm for $\widehat{\Theta}_1$. Dimensions from 100 to 2000 were analysed. Σ_1 was generated using the covariance 1 method multiple block, using $p = 100, 200, 500, 1000, 2000$. The sample data sets were created using the sampling method mvrnorm with $n = 50$. *Clime* and *gdtrace* were only analysed in the approaches with $p = 100$ and $p = 100, 200$, respectively.

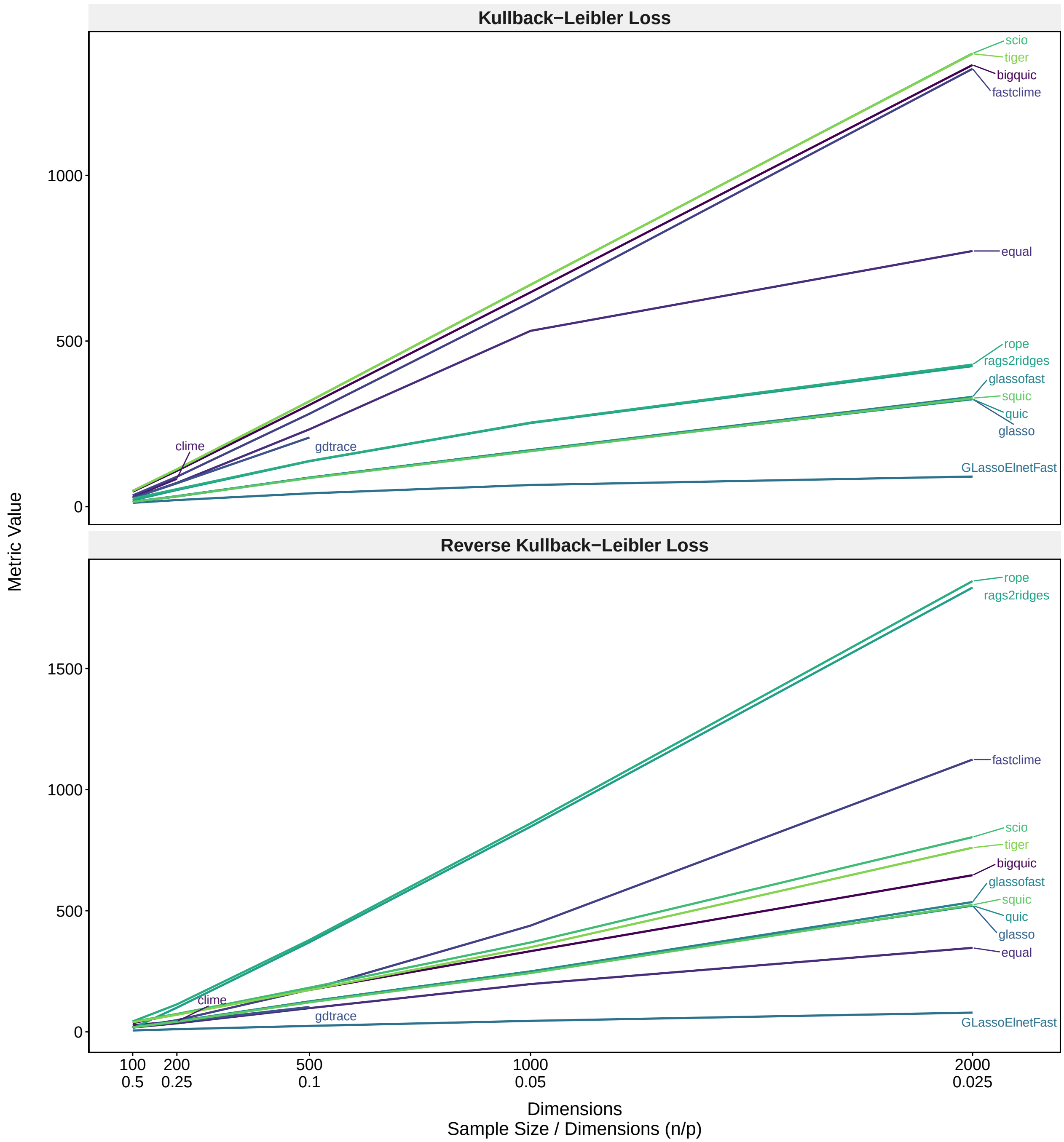

Supplementary Figure 32: Line plot illustrating the impact of increasing the dimensions on the performances of precision matrix estimation methods, assessed across the Kullback-Leibler Loss and the reverse Kullback-Leibler Loss for $\widehat{\Theta}_1$. Dimensions from 100 to 2000 were analysed. Σ_1 was generated using the covariance 1 method multiple block, using $p = 100, 200, 500, 1000, 2000$. The sample data sets were created using the sampling method mvrnorm with $n = 50$. *Clime* and *gdtrace* were only analysed in the approaches with $p = 100$ and $p = 100, 200$, respectively.

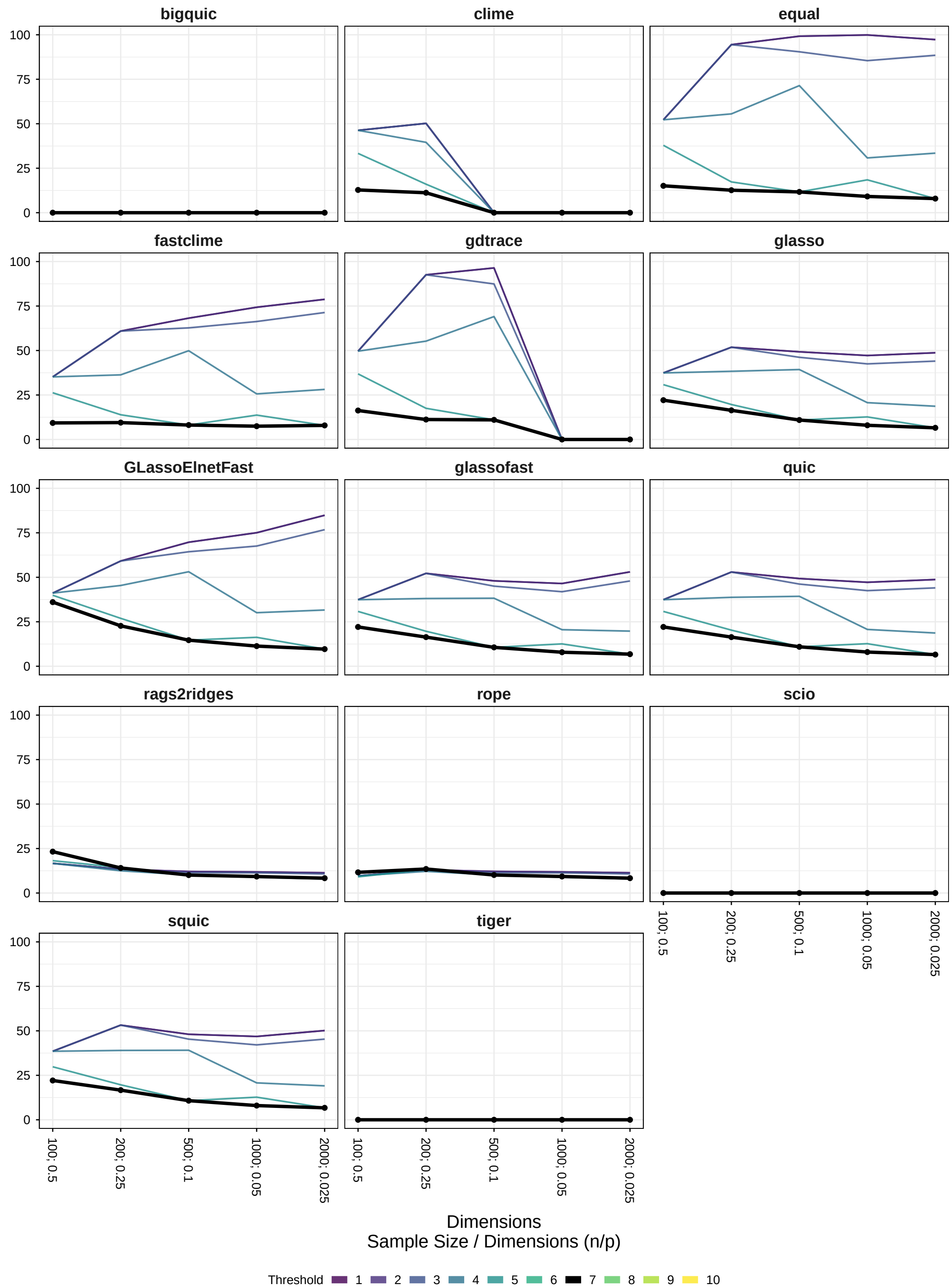

Supplementary Figure 33: Line plot illustrating the sensitivity of the Differential Edge Recovery (DER) score, computed on $\widehat{\Psi}_\Delta$, to the weight threshold τ across increasing dimensions. Within each panel, DER is shown across dimensions for each of the $m = 10$ threshold values $\tau_1, \ldots, \tau_{10}$ generated as described in the Method section. The threshold used for the corresponding main-text figure is highlighted in black. Dimensions from 100 to 2000 were analysed. Σ_1 was generated using the covariance 1 method multiple block, using $p = 100, 200, 500, 1000, 2000$. The sample data sets were created using the sampling method mvrnorm with $n = 50$. *Clime* and *gdtrace* were only analysed in the approaches with $p = 100$ and $p = 100, 200$, respectively.

8 Supplementary Information for Means

Next, the effect of shifting the mean values of the variable distributions in the simulated datasets was evaluated. While the covariance structure remained constant, the mean vectors were either set to zero, fixed at ten, or randomly sampled within the range of five to fifteen. Results are shown in Supplementary Figures 34, 35, and 36. Overall, no visible impact on performance was observed for any PMEM across the binary classification metrics, matrix norms, or Kullback-Leibler losses.

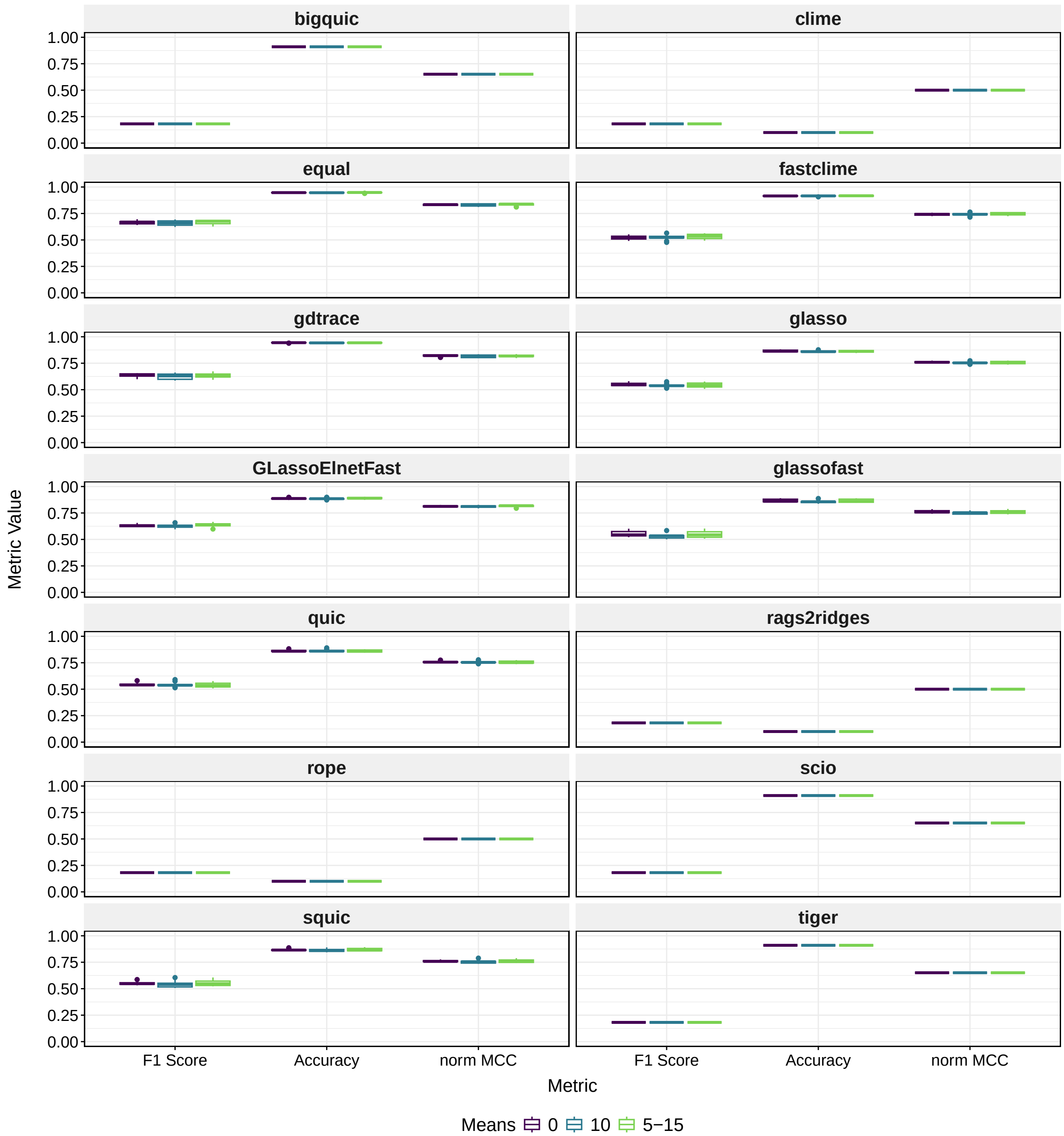

Supplementary Figure 34: Boxplot illustrating the effect of shifting means on the performance of different PMEMs in repeated simulations, assessed across the binary classification metrics F1 score, accuracy, and normalized MCC. The performance of each method is represented by the distribution of the metric values over 10 replicates for each mean. Σ_1 was generated using the covariance 1 method multiple block, using $p = 100$. The sample datasets were generated using the mvrnorm sampling method with $n = 50$, and the variable means were set to 0, to 10, or randomly drawn from the range 5 to 15.

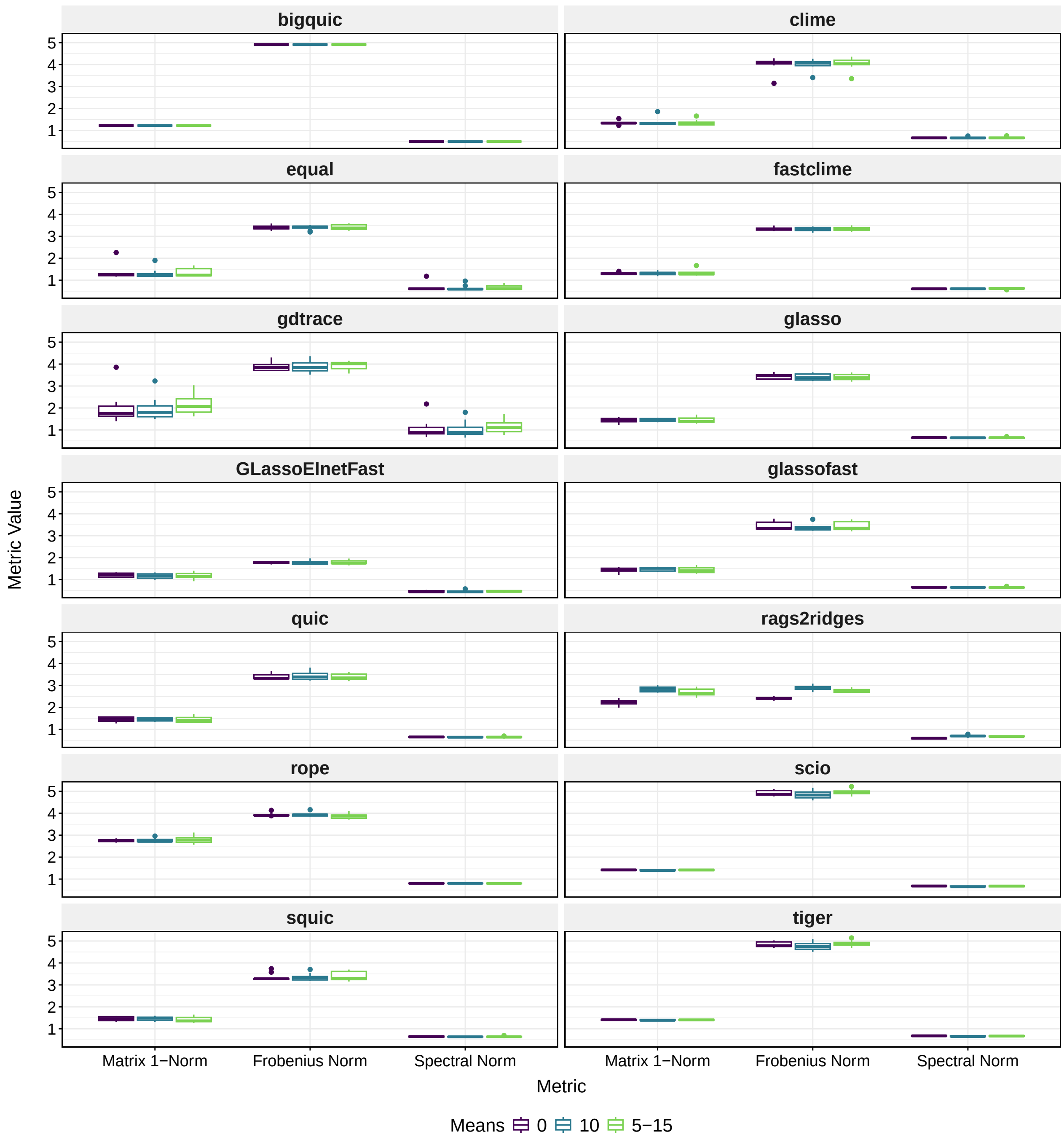

Supplementary Figure 35: Boxplot illustrating the effect of shifting means on the performance of different PMEMs in repeated simulations, assessed across the matrix norms matrix 1-norm, Frobenius norm, and spectral norm. The performance of each method is represented by the distribution of the metric values over 10 replicates for each mean. Σ_1 was generated using the covariance 1 method multiple block, using $p = 100$. The sample datasets were generated using the mvrnorm sampling method with $n = 50$, and the variable means were set to 0, to 10, or randomly drawn from the range 5 to 15.

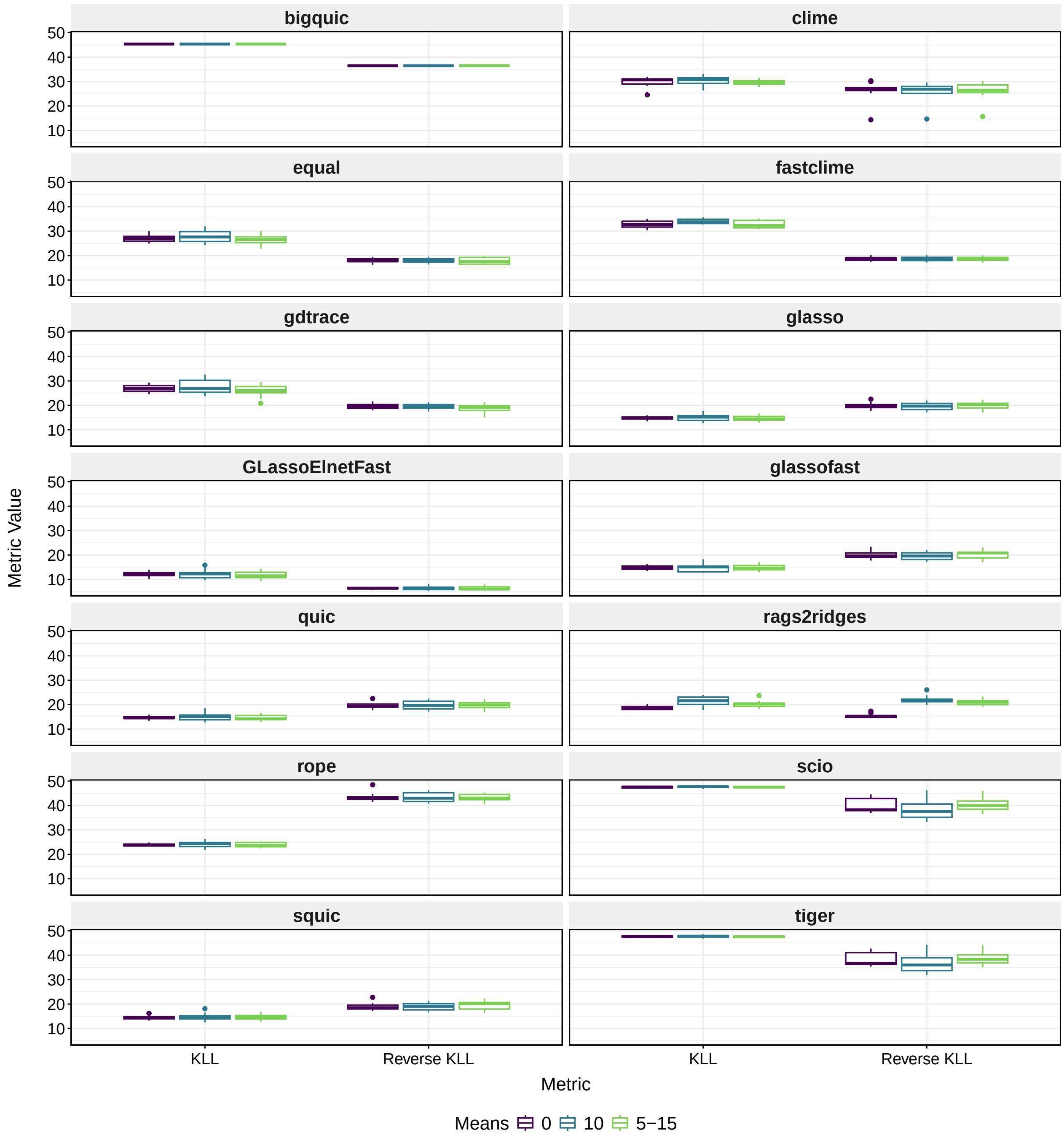

Supplementary Figure 36: Boxplot illustrating the effect of shifting means on the performance of different PMEMs in repeated simulations, assessed across the Kullback-Leibler Loss and the reverse Kullback-Leibler Loss. The performance of each method is represented by the distribution of the metric values over 10 replicates for each mean. Σ_1 was generated using the covariance 1 method multiple block, using $p = 100$. The sample datasets were generated using the mvrnorm sampling method with $n = 50$, and the variable means were set to 0, to 10, or randomly drawn from the range 5 to 15.

9 Supplementary Information for Normalization

In this approach, we assessed the impact of two normalization strategies using datasets, where variable means were randomly sampled in the range of five to fifteen, as in the previous analysis. Normalization strategy 1 standardizes the variable distributions to have both zero mean and unit variance. In the second normalization approach, variables are mean-centered only, retaining their original variances. The resulting performance differences are summarized in Supplementary Figures 37, 38, and 39. Binary classification metrics reveal a slight performance decrease for *GLassoElnetFast* when applying normalization strategy 1, while no notable differences are observed between the non-normalized data and normalization strategy 2 for any PMEM (Figure S37). The matrix norm values increase across nearly all PMEMs when applying normalization 1, indicating reduced performance. Only *scio* and *tiger* show decreased matrix norm values, suggesting improved estimates under this strategy. In contrast, normalization 2 has no visible impact on any of the matrix norms (Figure S38). Kullback-Leibler losses increase for all PMEMs under normalization 1, while reverse Kullback-Leibler losses tend to decrease. Again, normalization 2 does not influence performance (Figure S39).

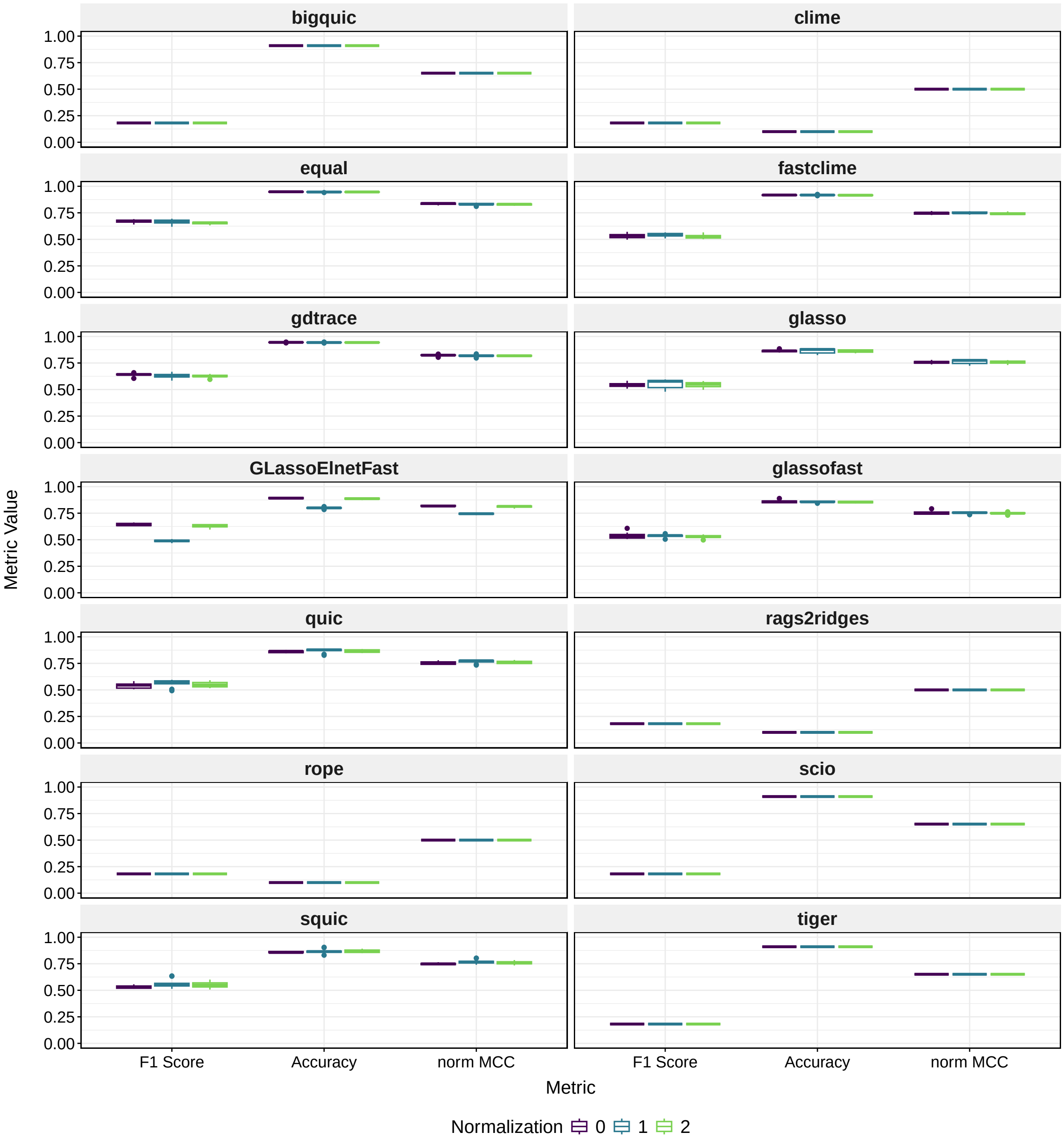

Supplementary Figure 37: Boxplot illustrating the effect of different normalization methods on the performance of different PMEMs in repeated simulations, assessed across the binary classification metrics F1 score, accuracy, and normalized MCC. The performance of each method is represented by the distribution of the metric values over 10 replicates for each mean. Σ_1 was generated using the covariance 1 method multiple block, using $p = 100$. The sample datasets were generated using the mvrnorm sampling method with $n = 50$, and the variable means were randomly drawn from the range 5 to 15. After sampling the following normalization strategies are applied, 0: no normalization, 1: normalizing means to 0 and variances to 1, 2: normalizing means to 0.

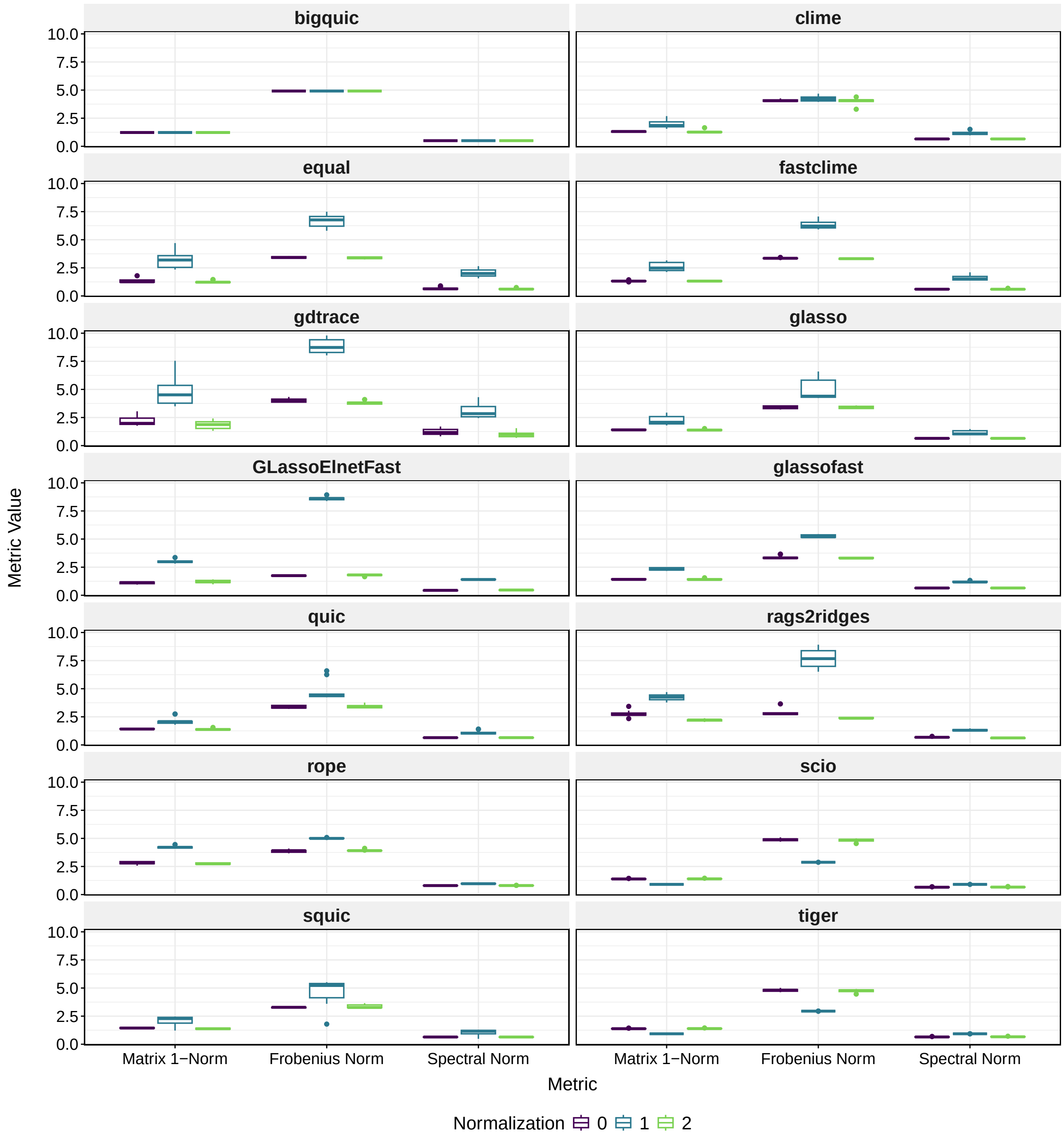

Supplementary Figure 38: Boxplot illustrating the effect of different normalization methods on the performance of different PMEMs in repeated simulations, assessed across the matrix norms matrix 1-norm, Frobenius norm, and spectral norm. The performance of each method is represented by the distribution of the metric values over 10 replicates for each mean. Σ_1 was generated using the covariance 1 method multiple block, using $p = 100$. The sample datasets were generated using the mvrnorm sampling method with $n = 50$, and the variable means were randomly drawn from the range 5 to 15. After sampling the following normalization strategies are applied, 0: no normalization, 1: normalizing means to 0 and variances to 1, 2: normalizing means to 0.

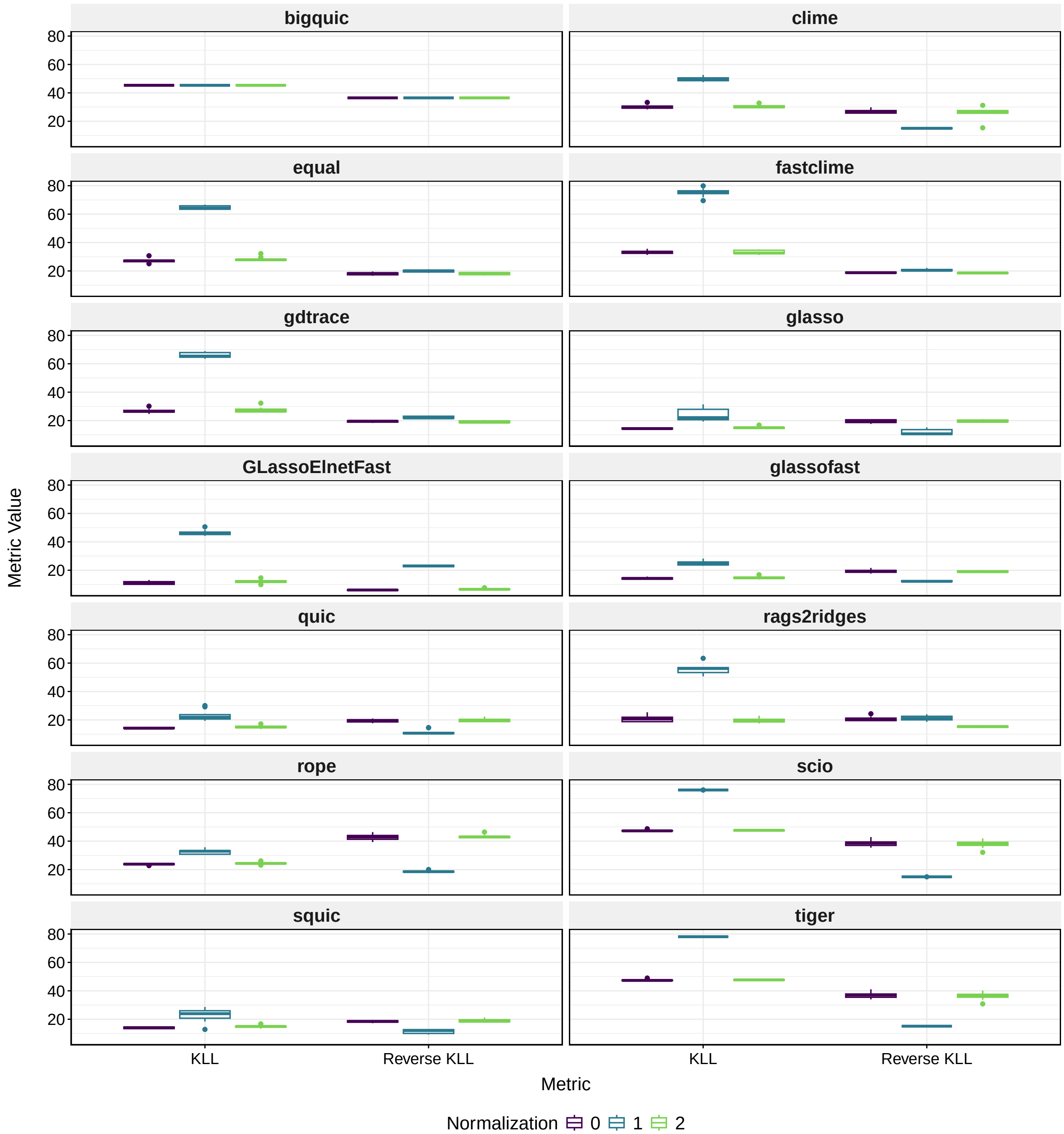

Supplementary Figure 39: Boxplot illustrating the effect of different normalization methods on the performance of different PMEMs in repeated simulations, assessed across the Kullback-Leibler Loss and the reverse Kullback-Leibler Loss. The performance of each method is represented by the distribution of the metric values over 10 replicates for each mean. Σ_1 was generated using the covariance 1 method multiple block, using $p = 100$. The sample datasets were generated using the mvrnorm sampling method with $n = 50$, and the variable means were randomly drawn from the range 5 to 15. After sampling the following normalization strategies are applied, 0: no normalization, 1: normalizing means to 0 and variances to 1, 2: normalizing means to 0.

10 Supplementary Figures for Sample Size

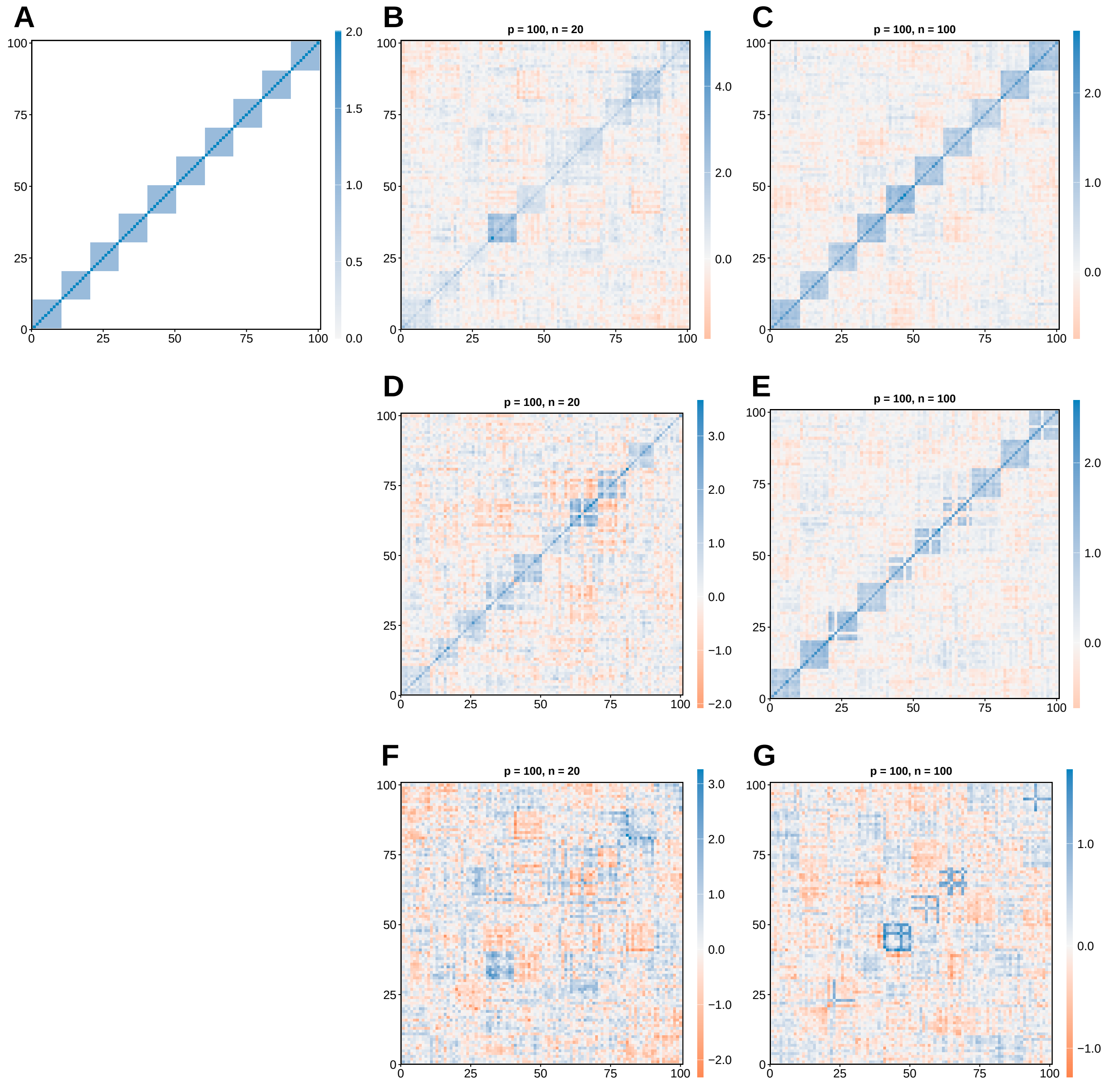

Supplementary Figure 40: A: Σ_1 generated with the multiple block method, using $p = 100$. B & C: S_1 generated with the mvrnorm method based on Σ_1, using $n = 20, 100$, respectively. D & E: S_2 generated with the mvrnorm method, using $n = 20, 100$, respectively. F & G: The respective differential sample covariance matrices S_Δ.

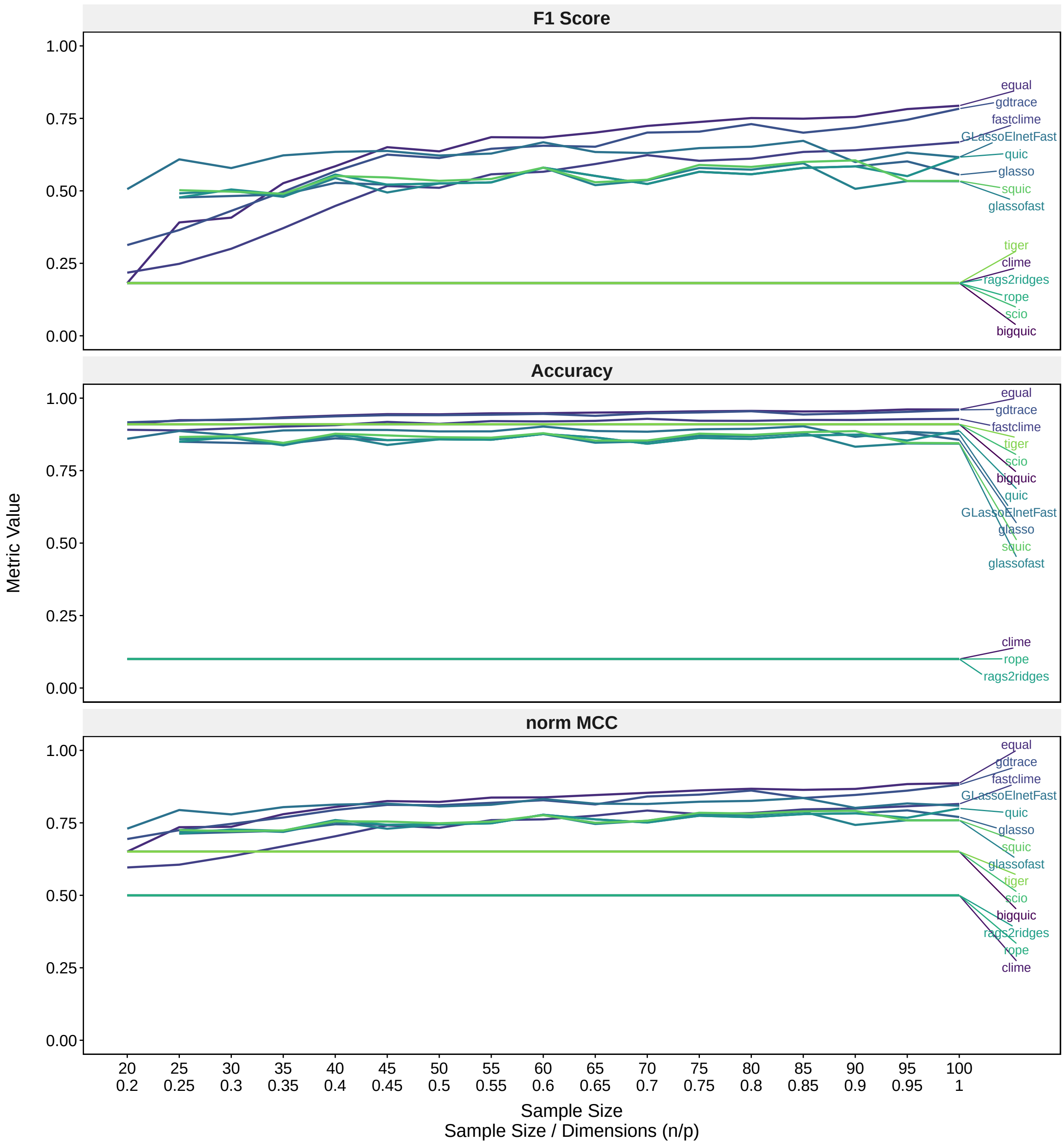

Supplementary Figure 41: Line plot illustrating the impact of increasing sample sizes on the performances of precision matrix estimation methods, assessed across the binary classification metrics F1 score, accuracy, and normalized MCC. Sample sizes from 20 to 100 were analysed. Σ_1 was generated using the covariance 1 method multiple block, with $p = 100$. The simulated data sets were created using the sampling method mvrnorm with $n = 20, 25, \ldots, 95, 100$.

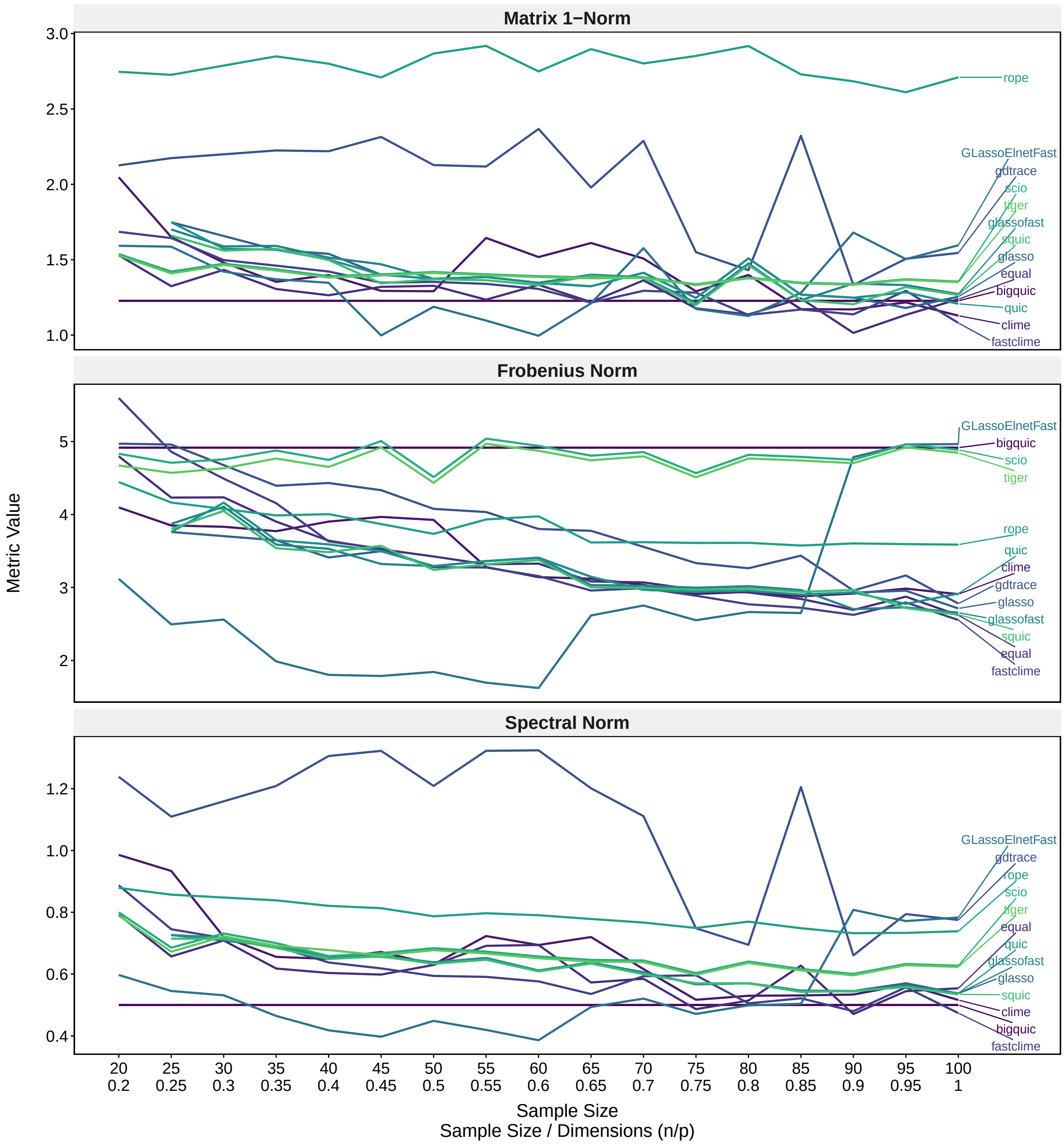

Supplementary Figure 42: Line plot illustrating the impact of increasing sample sizes on the performances of precision matrix estimation methods, assessed across the matrix norms matrix 1-norm, Frobenius norm, and spectral norm. Sample sizes from 20 to 100 were analysed. Σ_1 was generated using the covariance 1 method multiple block, with $p = 100$. The simulated data sets were created using the sampling method mvrnorm with $n = 20, 25, \ldots, 95, 100$. *Rags2ridges* is excluded from this graph as its exceptionally high norm values obscured the differences among the remaining methods. Figure S43 includes *rags2ridges*.

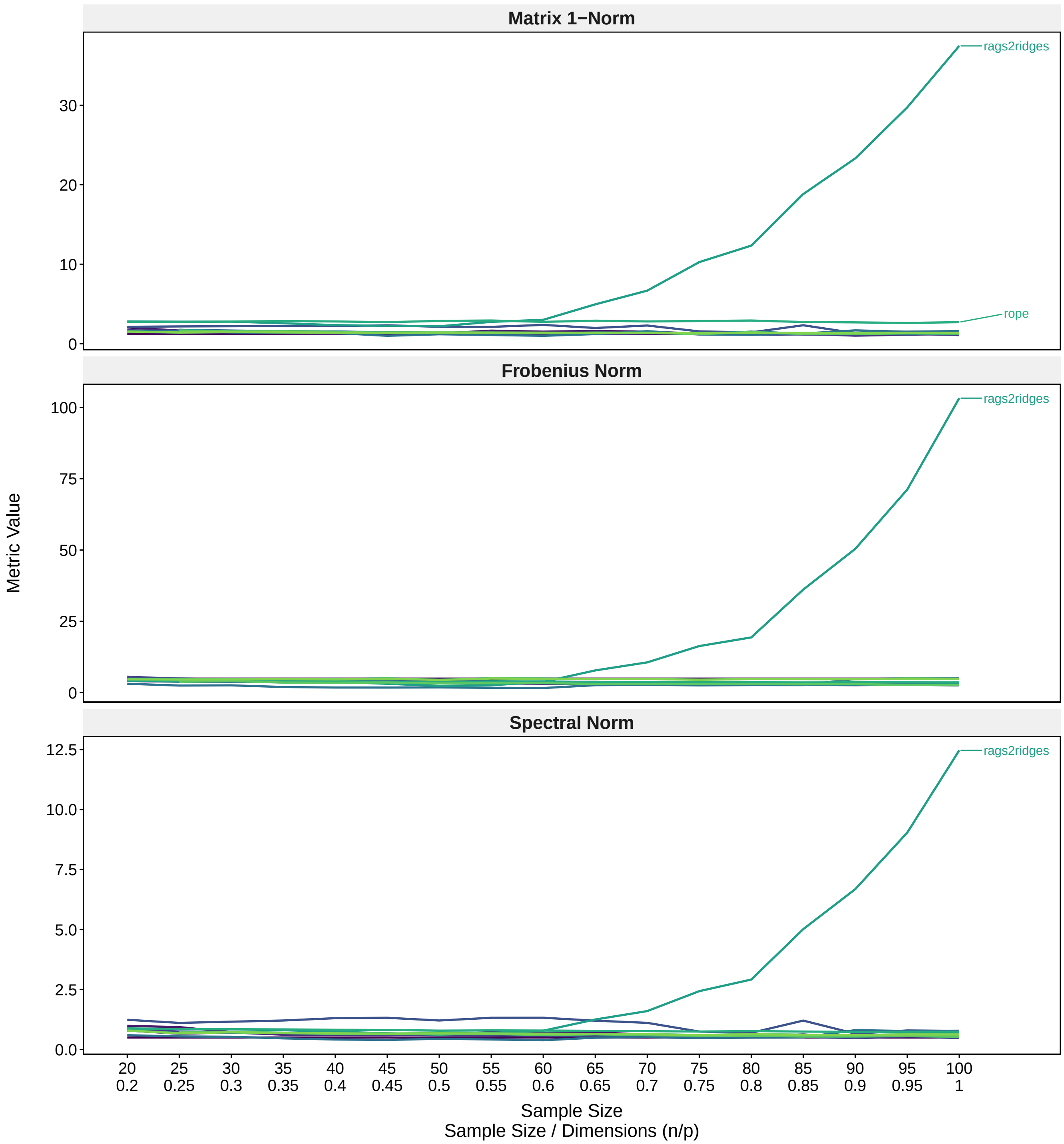

Supplementary Figure 43: Line plot illustrating the impact of increasing sample sizes on the performances of precision matrix estimation methods, assessed across the matrix norms matrix 1-norm, Frobenius norm, and spectral norm. Sample sizes from 20 to 100 were analysed. Σ_1 was generated using the covariance 1 method multiple block, with $p = 100$. The simulated data sets were created using the sampling method mvrnorm with $n = 20, 25, \ldots, 95, 100$.

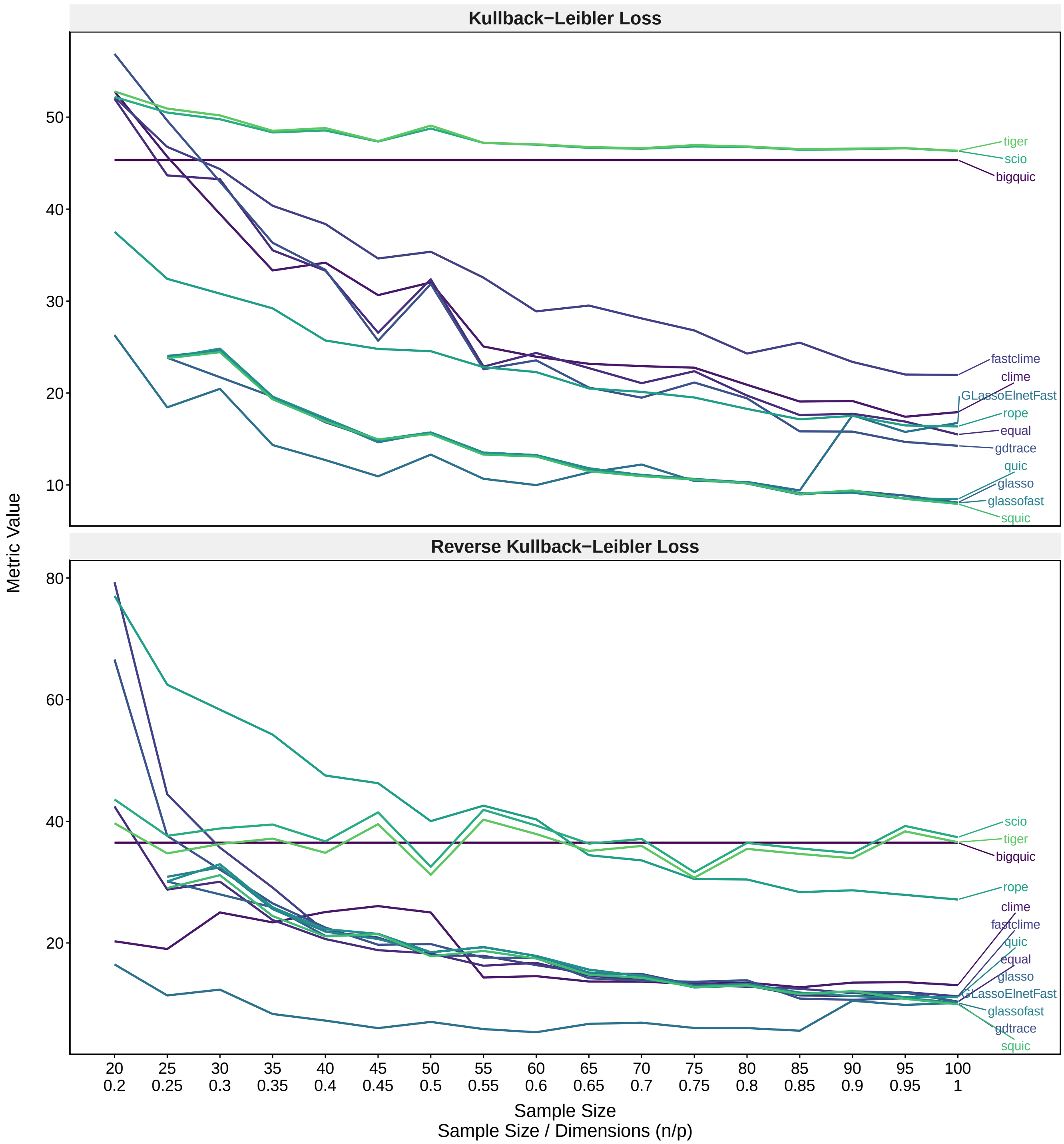

Supplementary Figure 44: Line plot illustrating the impact of increasing sample sizes on the performances of precision matrix estimation methods, assessed across across the Kullback-Leibler Loss and the reverse Kullback-Leibler Loss. Sample sizes from 20 to 100 were analysed. Σ_1 was generated using the covariance 1 method multiple block, with $p = 100$. The simulated data sets were created using the sampling method mvrnorm with $n = 20, 25, \ldots, 95, 100$. *Rags2ridges* is excluded from this graph as its exceptionally high loss values obscured the differences among the remaining methods. Figure S45 includes *rags2ridges*.

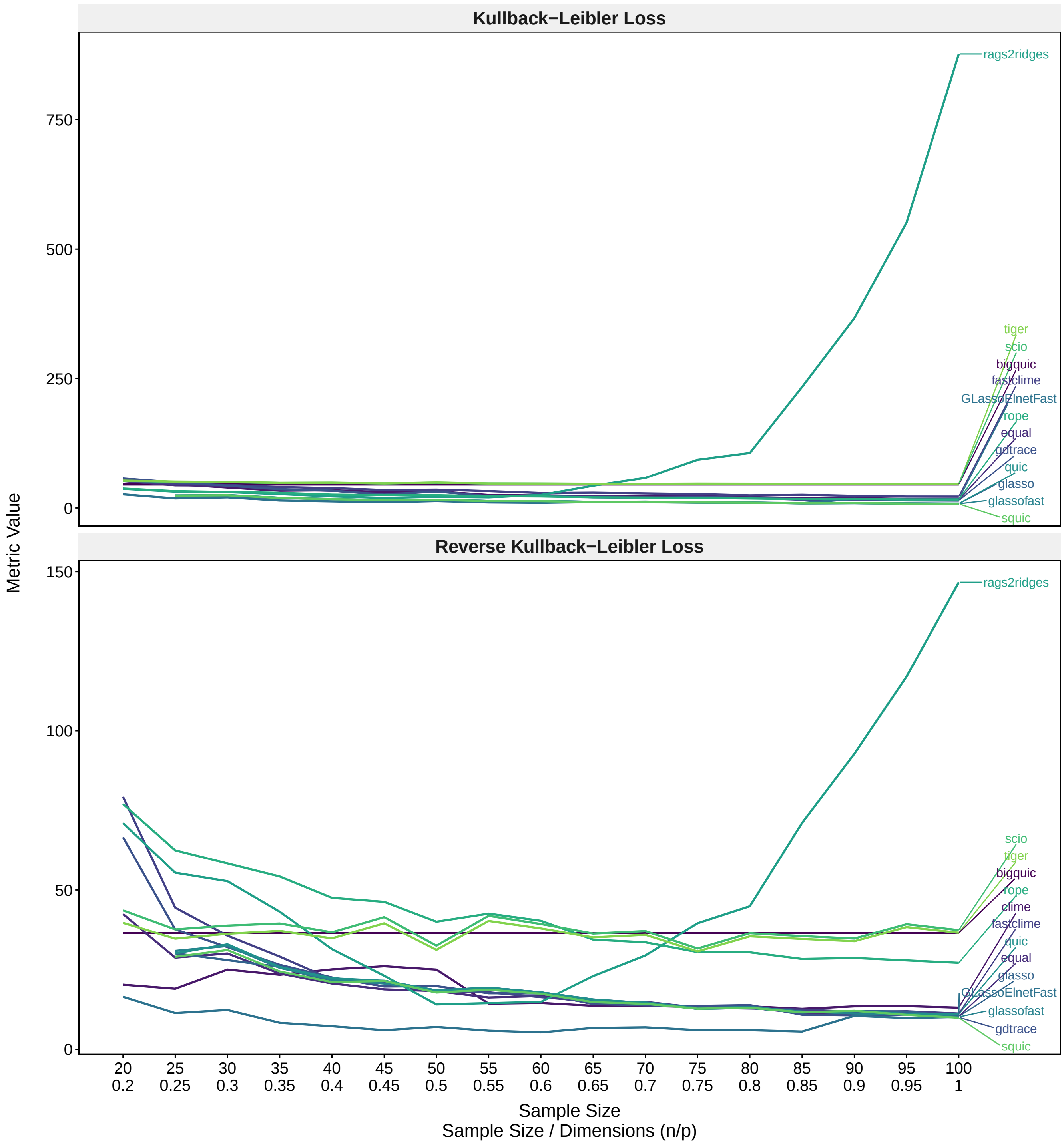

Supplementary Figure 45: Line plot illustrating the impact of increasing sample sizes on the performances of precision matrix estimation methods, assessed across across the Kullback-Leibler Loss and the reverse Kullback-Leibler Loss. Sample sizes from 20 to 100 were analysed. Σ_1 was generated using the covariance 1 method multiple block, with $p = 100$. The simulated data sets were created using the sampling method mvrnorm with $n = 20, 25, \ldots, 95, 100$.

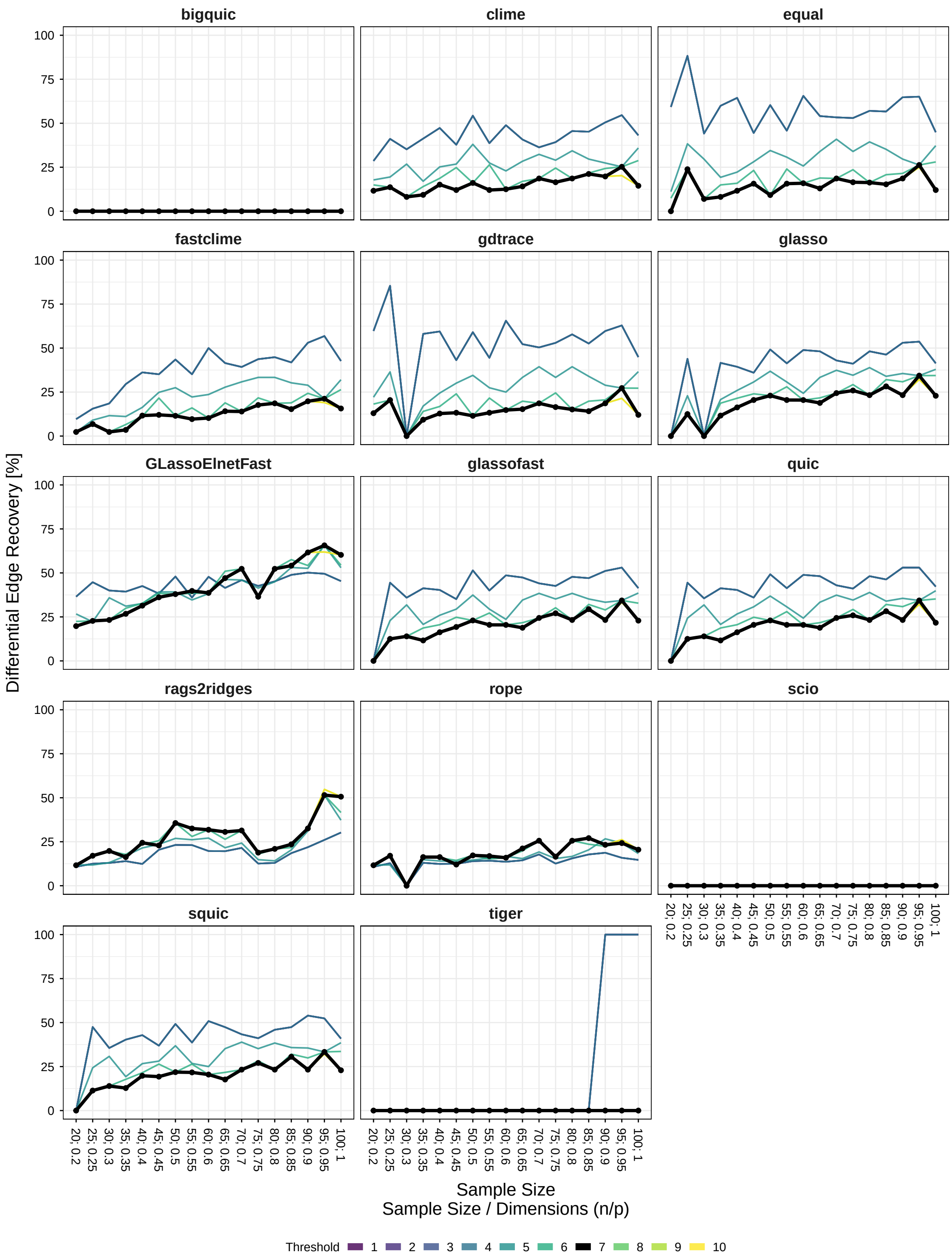

Supplementary Figure 46: Line plot illustrating the sensitivity of the Differential Edge Recovery (DER) score, computed on $\widehat{\Psi}_\Delta$, to the weight threshold τ across increasing sample sizes. Within each panel, DER is shown across sample sizes for each of the $m = 10$ threshold values $\tau_1, \ldots, \tau_{10}$ generated as described in the Method section. The threshold used for the corresponding main-text figure is highlighted in black. Sample sizes from 20 to 100 were analysed. Σ_1 was generated using the covariance 1 method multiple block, with $p = 100$. The simulated data sets were created using the sampling method mvrnorm with $n = 20, 25, \ldots, 95, 100$.

11 Supplementary Information for Sampling

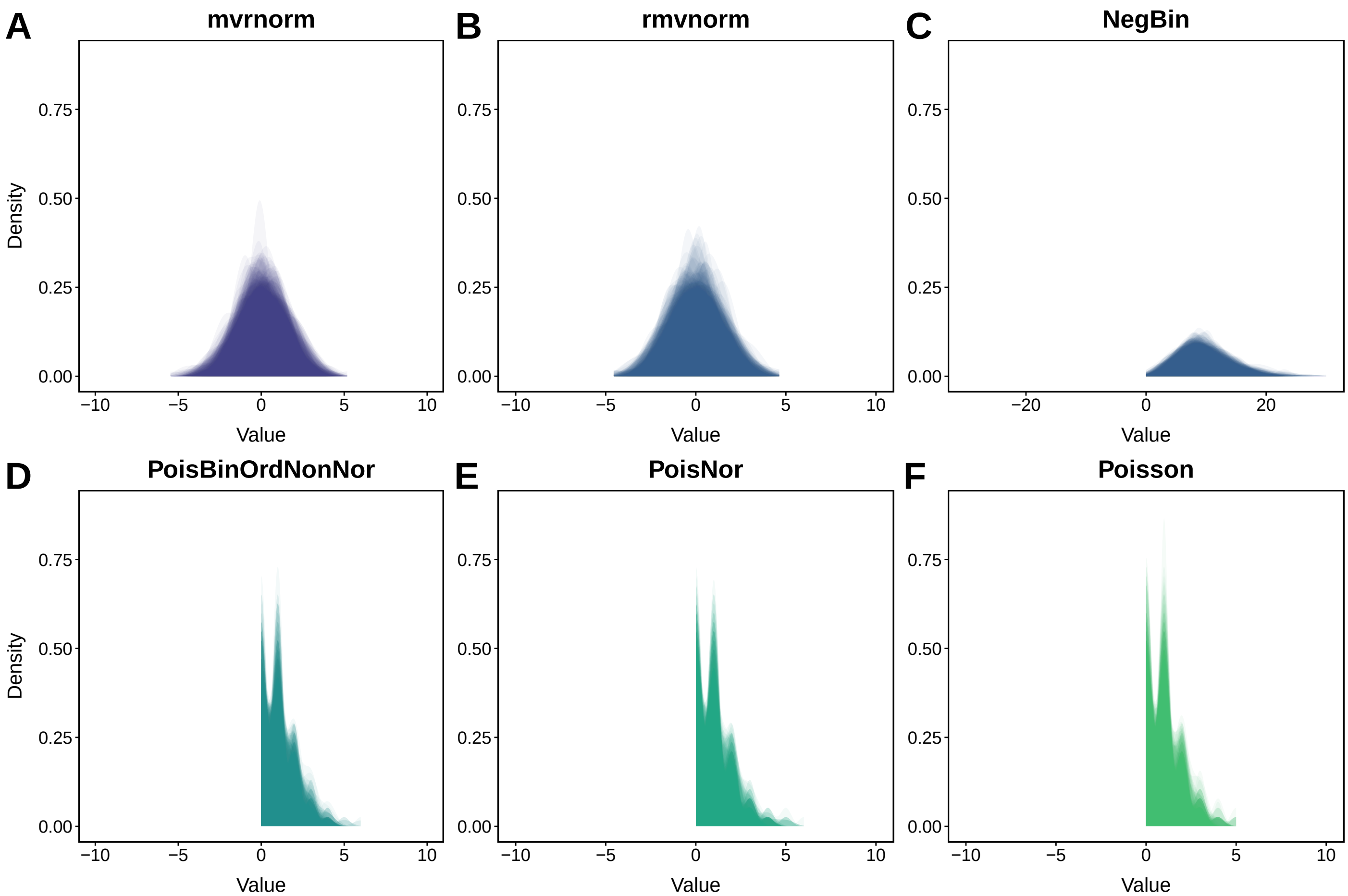

Supplementary Figure 47: Superimposed distributions of 100 variables across 50 samples from the simulated data sets for condition 1, generated by the sampling methods mvrnorm, rmvnorm, NegBin, PoisNor, PoisBinOrdNonNor, and Poisson.

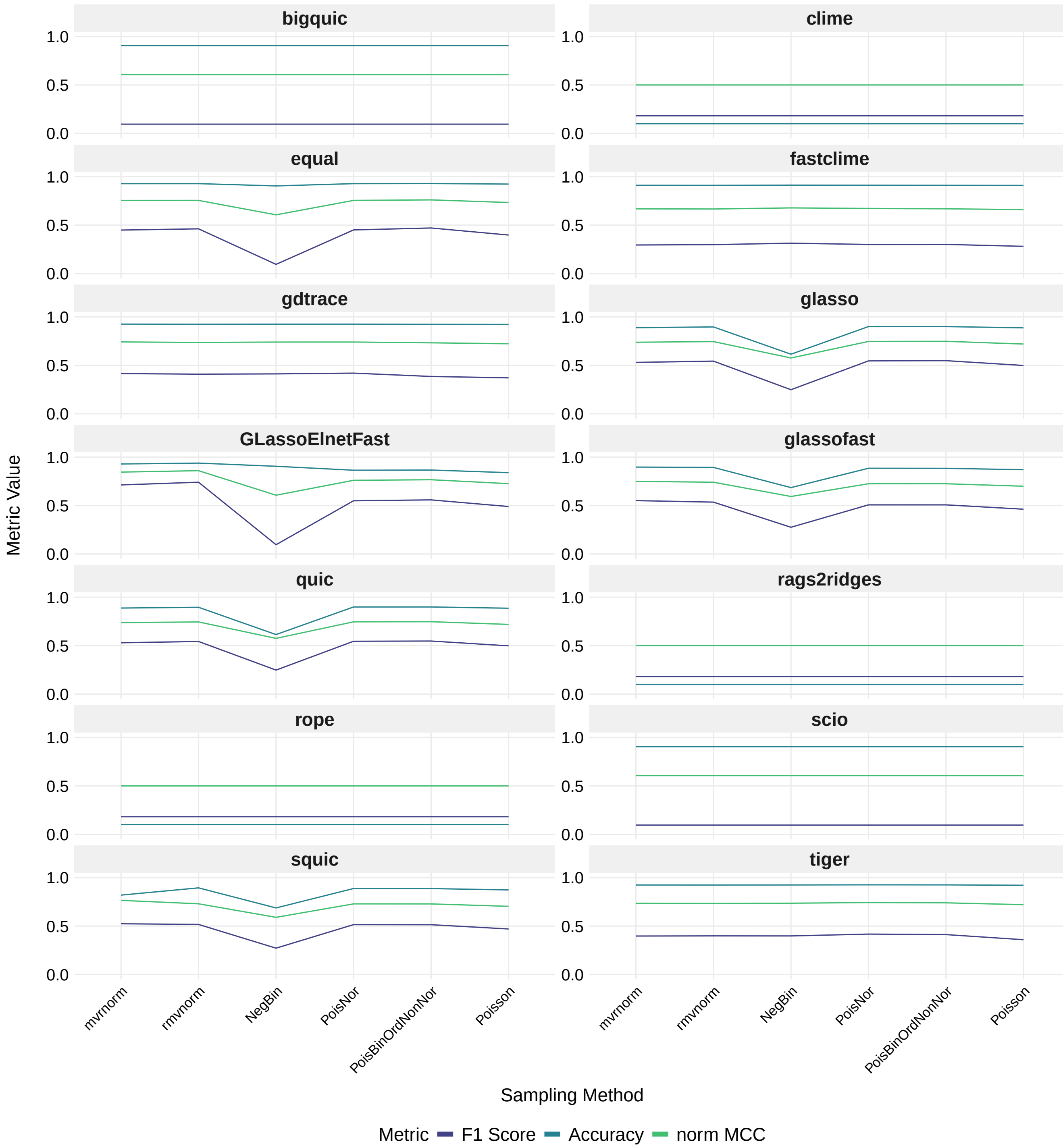

Supplementary Figure 48: Line plot illustrating the influence of different sampling methods on the performances of precision matrix estimation methods, assessed across the binary classification metrics F1 score, accuracy, and normalized MCC. Σ_1 was generated using the covariance 1 method multiple block with $p = 200$. The sample data set was created using the respective sampling method with $n = 50$.

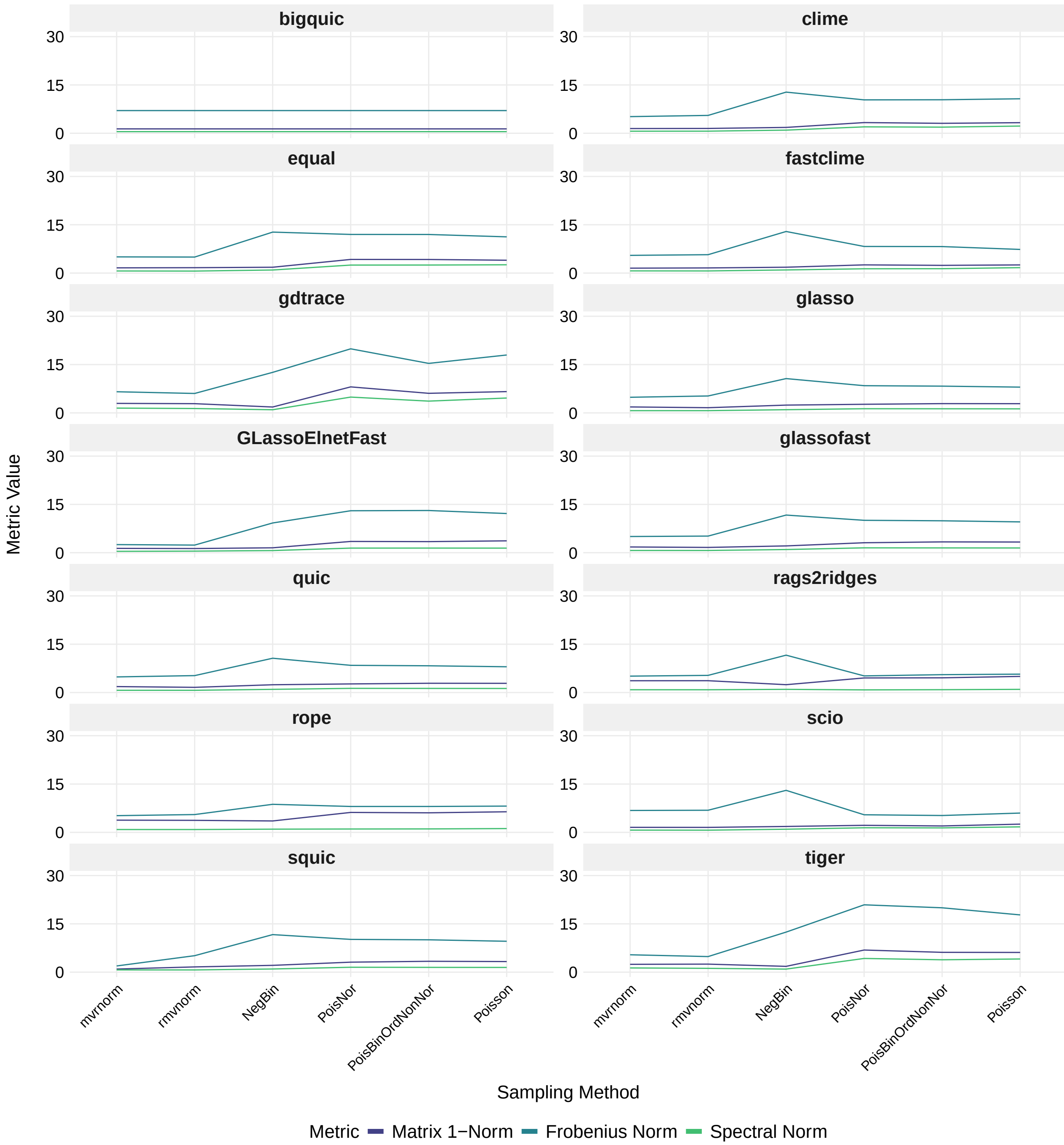

Supplementary Figure 49: Line plot illustrating the influence of different sampling methods on the performances of precision matrix estimation methods, assessed across the matrix norms matrix 1-norm, Frobenius norm, and spectral norm. Σ_1 was generated using the covariance 1 method multiple block with $p = 200$. The sample data set was created using the respective sampling method with $n = 50$.

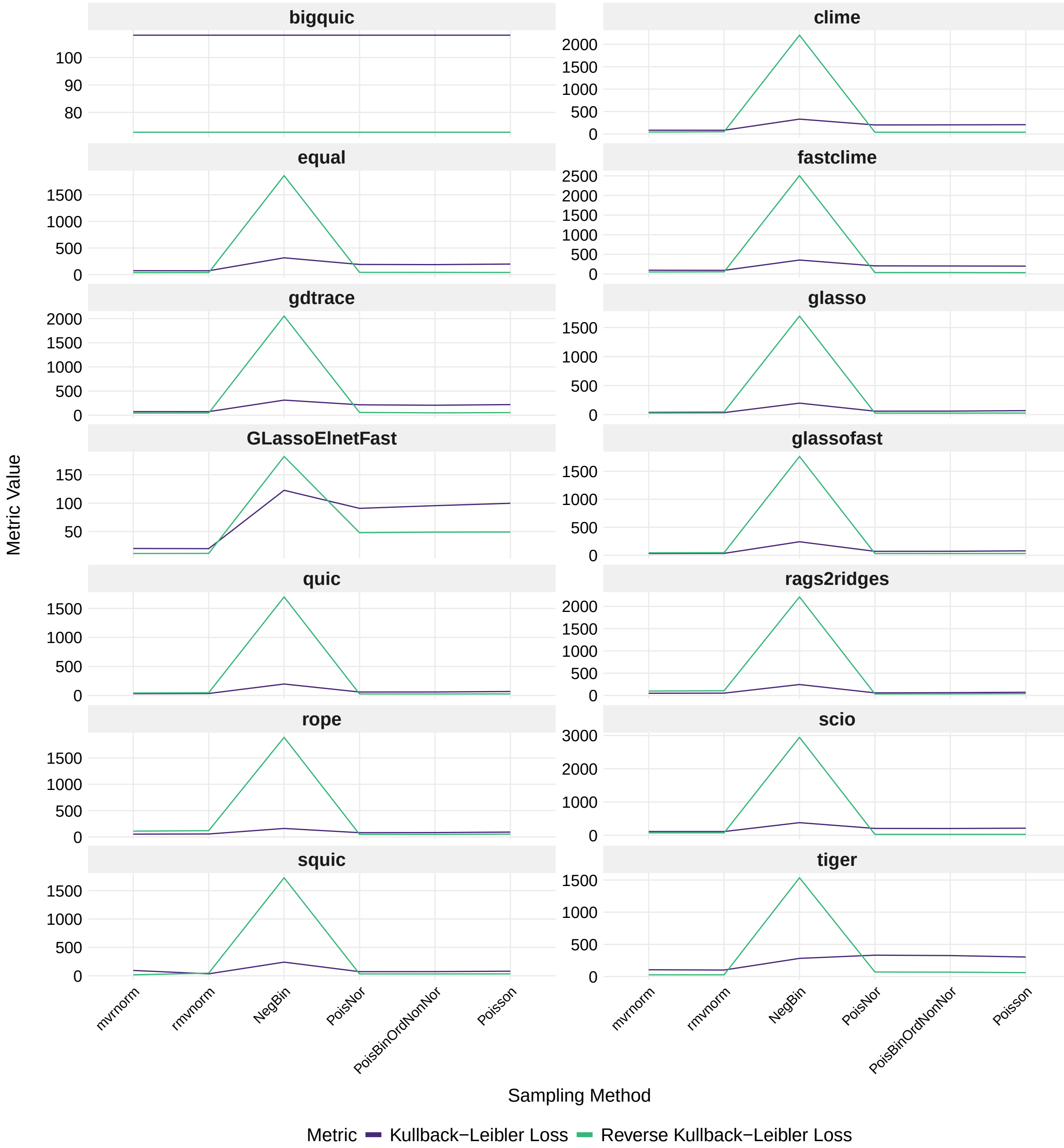

Supplementary Figure 50: Line plot illustrating the influence of different sampling methods on the performances of precision matrix estimation methods, assessed across the Kullback-Leibler Loss and the reverse Kullback-Leibler Loss. Σ_1 was generated using the covariance 1 method multiple block with $p = 200$. The sample data set was created using the respective sampling method with $n = 50$.

Yahav and Shmueli (2008) [3] proposed an approach for the generation of a p-dimensional Poisson vector with covariance matrix Σ_{Pois} and rate $\vec{\Lambda}$. Specifically, the method involves generating a multivariate normal vector and then transforming it into Poisson-distributed values using the cumulative distribution functions (CDFs) of both the normal and Poisson distributions. The CDF of the Gaussian and Poisson distribution are shown in Equation 5 and 6, respectively.

$$\Phi(x) = \int_{-\infty}^{x} \frac{1}{\sqrt{2\sigma^2}} e^{-\frac{u^2}{2\sigma^2}} du \tag{5}$$

$$\Xi(x) = \sum_{i=0}^{x} \frac{e^{-\lambda}\lambda^i}{i!} \tag{6}$$

Equation (5) transforms values from a normal distribution into cumulative probabilities, while Equation (6) transforms those cumulative probabilities into Poisson-distributed values, which allows the generation of Poisson random variables from normally distributed data. The algorithm proceeds as follows:

(a) Generate a p-dimensional normal vector $\vec{X}^N$ with mean $\vec{\mu} = 0$, variance $\vec{\sigma} = 1$, and a correlation matrix R^N.

(b) For each value X_i^N in the multivariate normal vector $\vec{X}^N$, compute the Normal CDF, resulting in values that follow a uniform distribution between 0 and 1:

$$\Phi(X_i^N)$$

(c) Transform the uniform values $\Phi(X_i^N)$ obtained in the previous step into Poisson-distributed values by applying the inverse of the Poisson CDF with the desired rate parameter λ_i:

$$X_i^{Pois} = \Xi^{-1}(\Phi(X_i^N))$$

The vector $\vec{X}^{\text{Pois}}$ is then a p-dimensional Poisson vector with correlation matrix R^{Pois} and rates $\vec{\Lambda}$.

[3] Yahav, I., & Shmueli, G. (2007). An elegant method for generating multivariate Poisson random variable. doi: 10.48550/ARXIV.0710.5670

Building on the same normal-to-anything (NORTA) construction described by Cario and Nelson (1997) [4] and applied above for the Poisson case, a p-dimensional negative binomial vector with covariance matrix Σ_{NB}, means $\vec{\mu}$, and dispersion parameters $\vec{r}$ (referred to as *size* in the implementation) was generated. As with the Poisson method, the approach involves generating a multivariate normal vector and then transforming it into negative binomial-distributed values using the CDFs of the normal and negative binomial distributions. The CDF of the Gaussian distribution is given in Equation 5; the CDF of the negative binomial distribution, parameterized by mean μ and dispersion r, is shown in Equation 7.

$$\Omega(x) = \sum_{i=0}^{x} \frac{\Gamma(i+r)}{\Gamma(r)\, i!} \left(\frac{r}{r+\mu}\right)^{r} \left(\frac{\mu}{r+\mu}\right)^{i} \tag{7}$$

Equation (7) transforms cumulative probabilities into negative binomial-distributed values, analogous to Equation (6) for the Poisson distribution. As $r \to \infty$, Equation (7) converges to Equation (6), recovering the Poisson distribution as a special case. The algorithm proceeds as follows:

(a) Generate a p-dimensional normal vector $\vec{X}^N$ with mean $\vec{\mu} = 0$, variance $\vec{\sigma} = 1$, and a correlation matrix R^N.

(b) For each value X_i^N in the multivariate normal vector $\vec{X}^N$, compute the Normal CDF, resulting in values that follow a uniform distribution between 0 and 1:

$$\Phi(X_i^N)$$

(c) Transform the uniform values $\Phi(X_i^N)$ obtained in the previous step into negative binomial-distributed values by applying the inverse of the negative binomial CDF with the desired mean μ_i and dispersion r_i:

$$X_i^{NB} = \Omega^{-1}(\Phi(X_i^N))$$

The vector $\vec{X}^{\text{NB}}$ is then a p-dimensional negative binomial vector with correlation matrix R^{NB}, means $\vec{\mu}$, and dispersion parameters $\vec{r}$.

[4] Cario, M. C., & Nelson, B. L. (1997). Modeling and generating random vectors with arbitrary marginal distributions and correlation matrix. Technical Report, Department of Industrial Engineering and Management Sciences, Northwestern University, Evanston, IL.

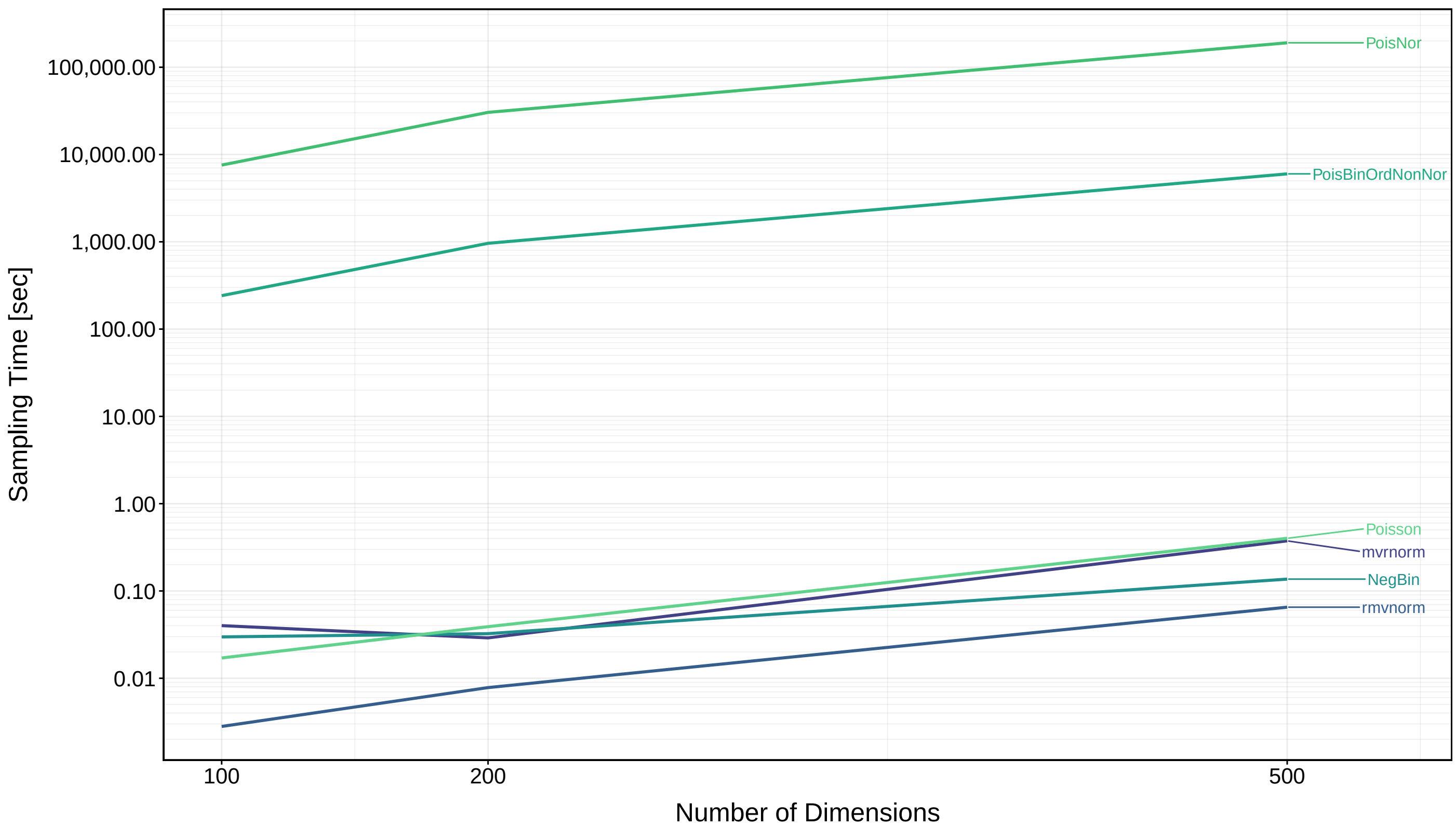

Supplementary Figure 51: Runtimes for the sampling methods mvrnorm and rmvnorm, NegBin, PoisNor, PoisBinOrdNonNor, and Poisson in relation to the amount of variables (dimensions). Σ_1 was created by the multiple block method, with a size of 100×100, 200×200 or 500×500. Σ_2 was generated using the knockout method. The respective sampling methods generated the corresponding simulated data sets for both condition using a sample size of 50. The runtime includes the generation of both data sets as well as the calculation of the sample covariance matrices S_1 and S_2.

12 Supplementary Figures for Runtimes

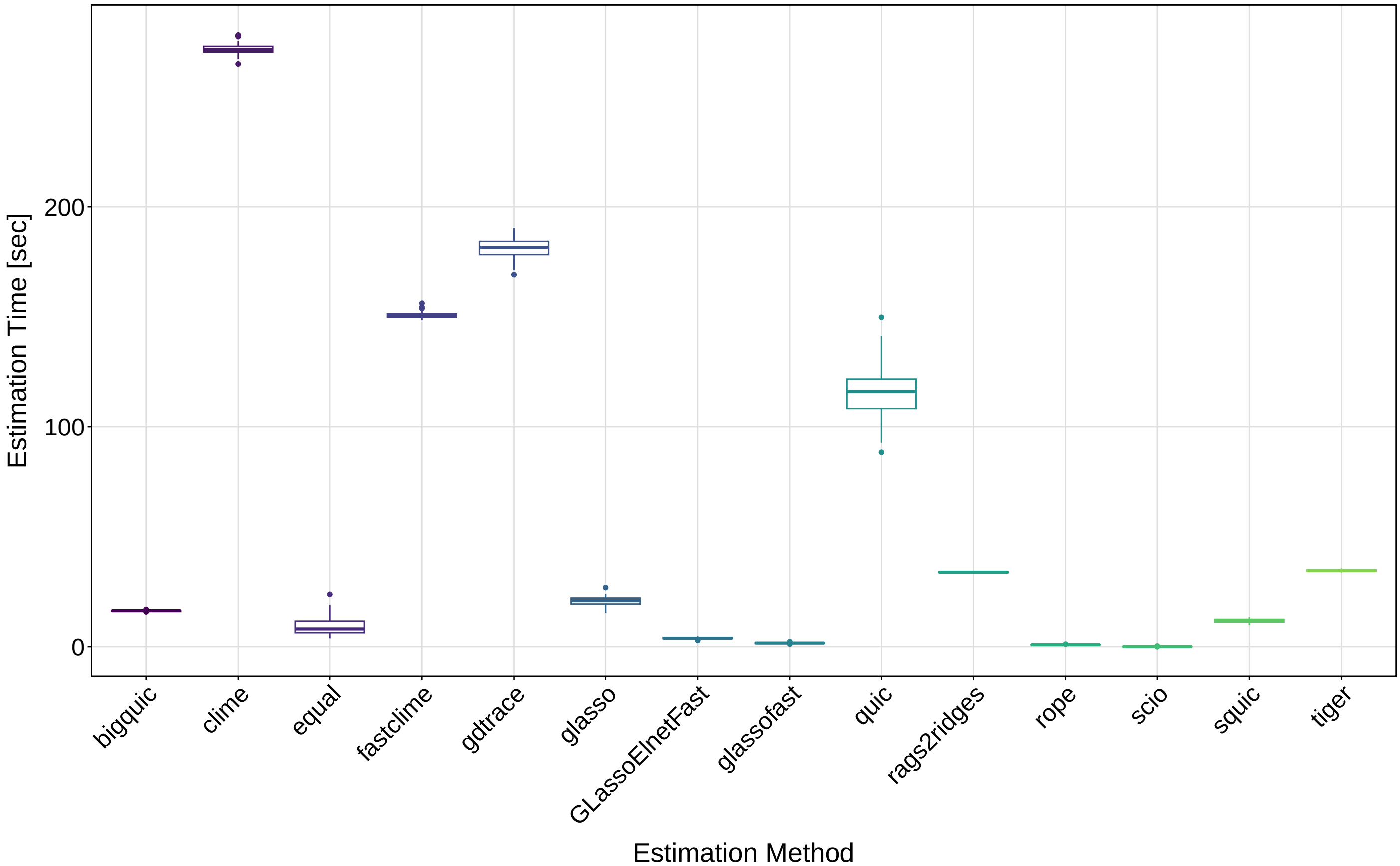

Supplementary Figure 52: Boxplot illustrating the variation in estimation times of different PMEMs over 50 replicates. Σ_1 was generated with the single block method, using $p = 100$. The sample data set was created using the mvrnorm sampling method with $n = 50$.

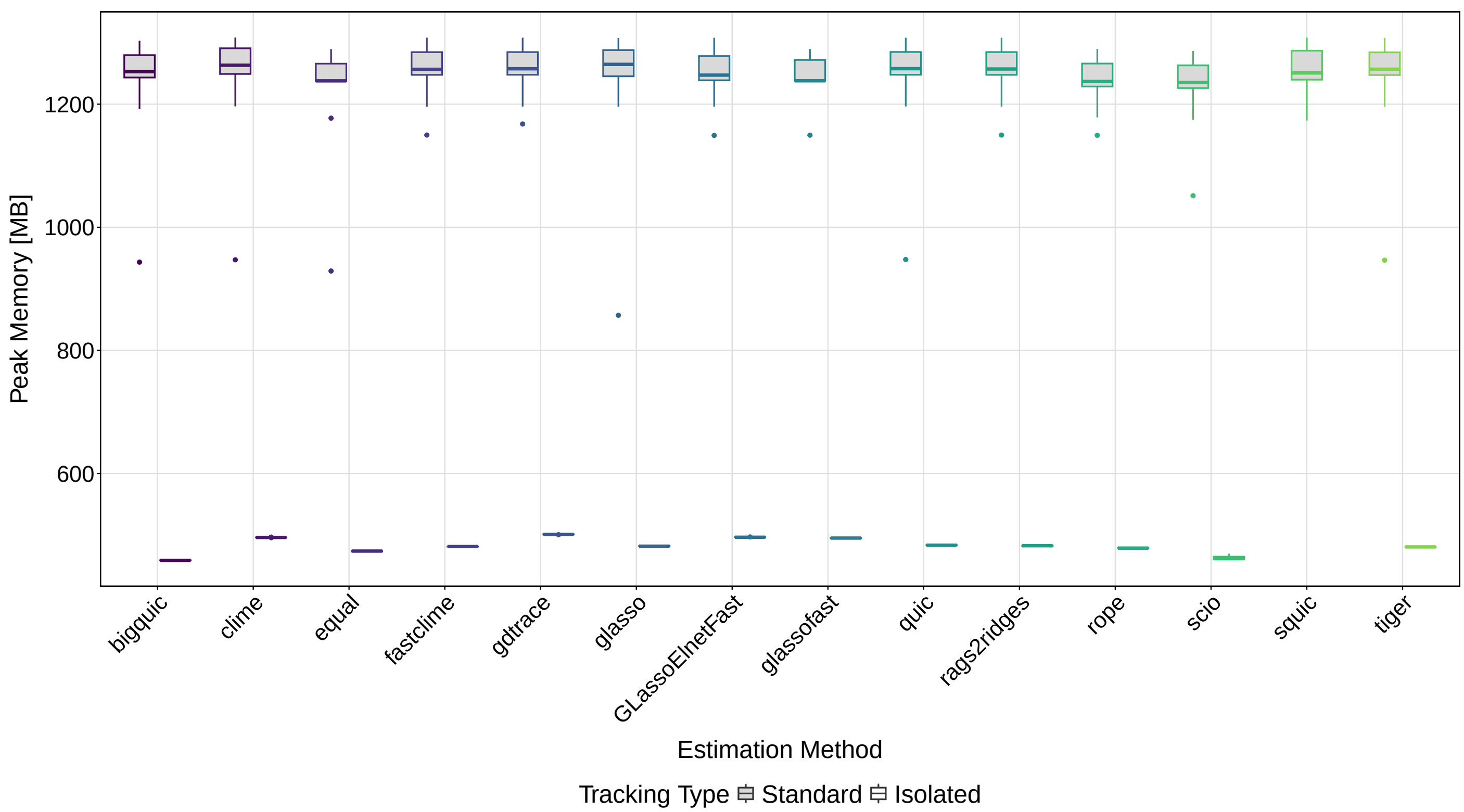

Supplementary Figure 53: Boxplot illustrating peak memory usage of different PMEMs over 10 replicates, comparing standard and isolated memory tracking. Σ_1 was generated with the single block method, using $p = 100$. The sample data set was created using the mvrnorm sampling method with $n = 50$. *squic* is shown for standard tracking only, as isolated tracking resulted in excessively long runtimes for this method.

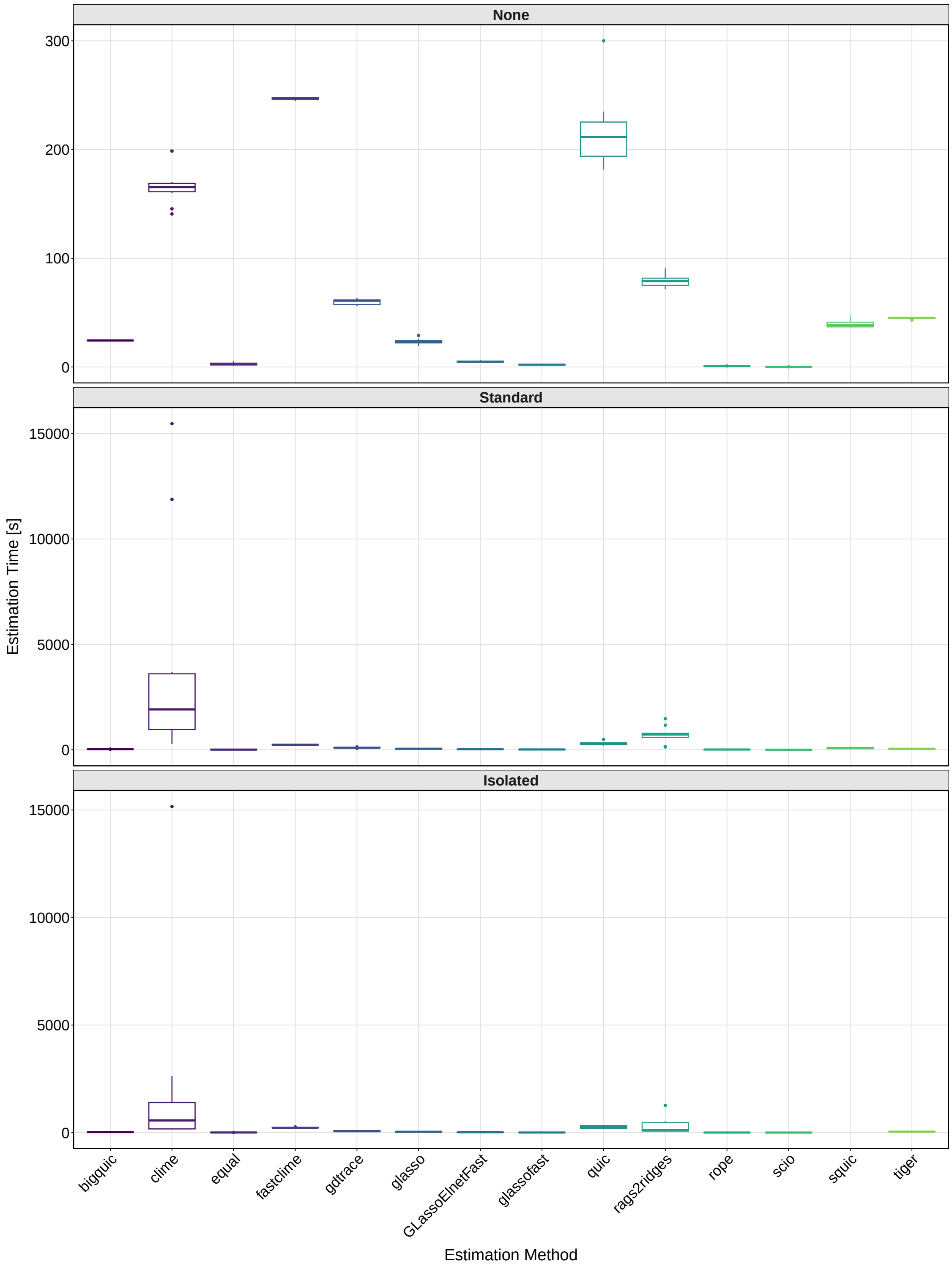

Supplementary Figure 54: Boxplot illustrating estimation runtimes of different PMEMs over 10 replicates under no, standard, and isolated memory tracking. Σ_1 was generated with the single block method, using $p = 100$. The sample data set was created using the mvrnorm sampling method with $n = 50$. *squic* is shown for the none and standard tracking modes only, as isolated tracking resulted in excessively long runtimes for this method.

13 Supplementary Figures for Real-World Analysis

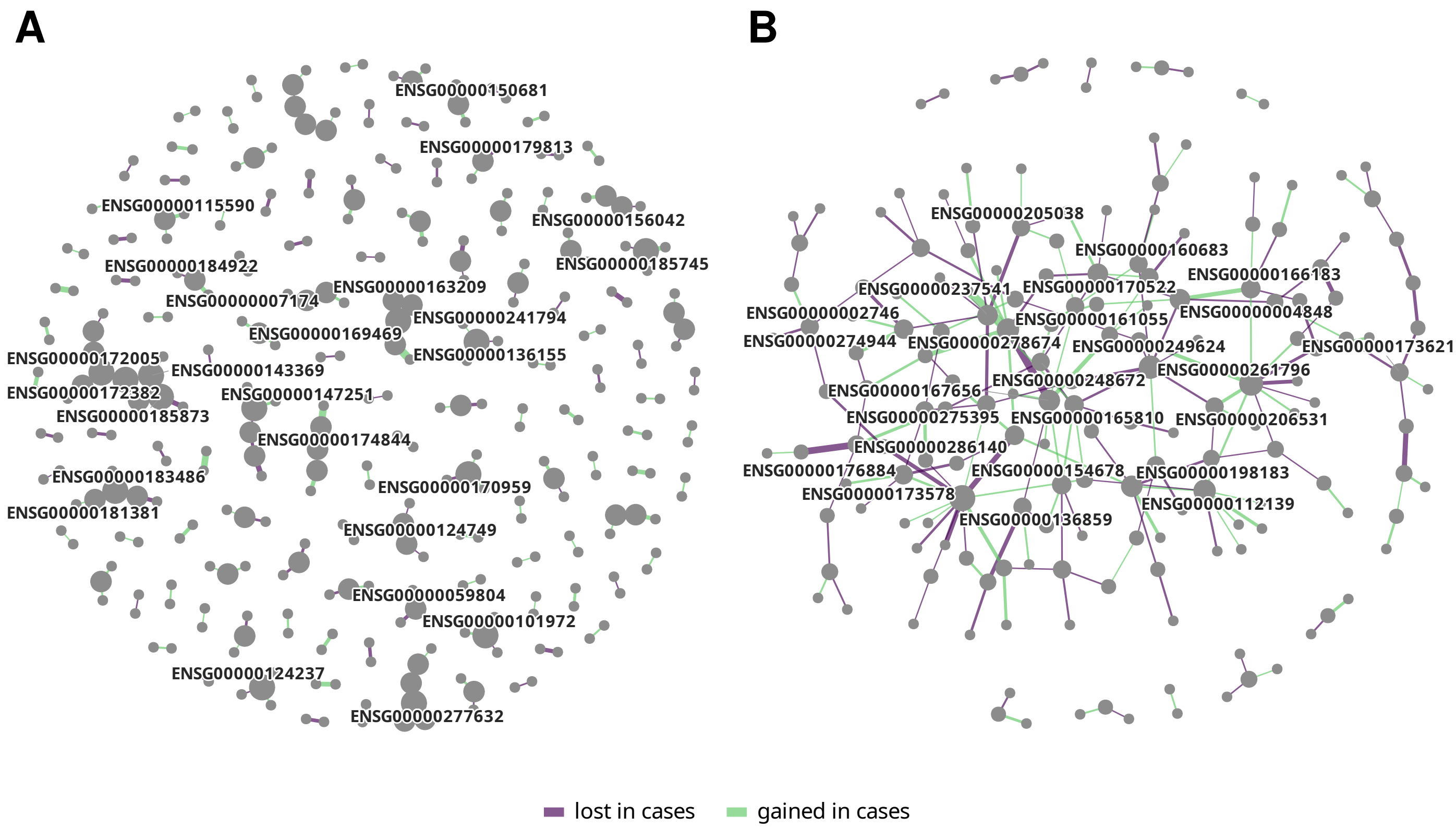

Supplementary Figure 55: Differential partial correlation networks for GSE156063 with COVID-19 (case) vs. non-viral ARI (control), estimated using *equal* (A) and *rags2ridges* (B).
